# Proteomic investigation of human skeletal muscle before and after 70 days of head down bed rest with or without exercise and testosterone countermeasures

**DOI:** 10.1371/journal.pone.0217690

**Published:** 2019-06-13

**Authors:** E. Lichar Dillon, Kizhake V. Soman, John E. Wiktorowicz, Ria Sur, Daniel Jupiter, Christopher P. Danesi, Kathleen M. Randolph, Charles R. Gilkison, William J. Durham, Randall J. Urban, Melinda Sheffield-Moore

**Affiliations:** 1 Department of Internal Medicine, The University of Texas Medical Branch, Galveston, TX, United States of America; 2 Department of Biochemistry and Molecular Biology, The University of Texas Medical Branch, Galveston, TX, United States of America; 3 Department of Preventive Medicine and Community Health, The University of Texas Medical Branch, Galveston, TX, United States of America; 4 Department of Health and Kinesiology, Texas A&M University, College Station, TX, United States of America; Universite de Caen Normandie, FRANCE

## Abstract

**Introduction:**

Long-term head-down bed rest (HDBR) results in musculoskeletal losses similar to those observed during long-term space flight. Agents such as testosterone, in addition to regular exercise, are effective countermeasures for reducing loss of skeletal muscle mass and function.

**Objective:**

We investigated the skeletal muscle proteome of healthy men in response to long term HDBR alone (CON) and to HDBR with exercise (PEX) or exercise plus testosterone (TEX) countermeasures.

**Method:**

Biopsies were performed on the *vastus lateralis* before (pre) HDBR and on HDBR days 32 (mid) and 64 (post). Extracted proteins from these skeletal muscle biopsies were subjected to 2-dimensional gel electrophoresis (2DE), stained for phosphoproteins (Pro-Q Diamond dye) and total proteins (Sypro Ruby dye). Proteins showing significant fold differences (t-test p ≤ 0.05) in abundance or phosphorylation state at mid or post were identified by mass spectroscopy (MS).

**Results:**

From a total of 932 protein spots, 130 spots were identified as potentially altered in terms of total protein or phosphoprotein levels due to HDBR and/or countermeasures, and 59 unique molecules emerged from MS analysis. Top canonical pathways identified through IPA included calcium signaling, actin cytoskeleton signaling, integrin linked kinase (ILK) signaling, and epithelial adherens junction signaling. Data from the pre-HDBR proteome supported the potential for predicting physiological post-HDBR responses such as the individual’s potential for loss vs. maintenance of muscle mass and strength.

**Conclusions:**

HDBR resulted in alterations to skeletal muscle abundances and phosphorylation of several structural and metabolic proteins. Inclusion of exercise alone or in combination with testosterone treatment modulated the proteomic responses towards cellular reorganization and hypertrophy, respectively. Finally, the baseline proteome may aid in the development of personalized countermeasures to mitigate health risks in astronauts as related to loss of muscle mass and function.

## Introduction

Skeletal muscle protein turnover is regulated through an intricate process involving biochemical and mechanical signals. Skeletal muscle size and composition is maintained when protein synthesis and breakdown is balanced while disruption of this balance can result in net gains or losses in muscle size and/or strength. Space flight related losses in muscle mass and strength are among the prime concerns for long duration space exploration missions and involve alterations in myofibrillar protein content and metabolism [1]. Skeletal muscle losses during space flight are largely attributed to absence of axial loading on weight bearing muscles, an environmental condition that can be mimicked adequately by bed rest studies on Earth. As such, the replacement of mechanical forces, via exercise, is among the primary operational countermeasures to mitigate muscle loss during space flight. While effective, exercise in space is time-consuming and only partially replaces the mechanical loading needed to completely prevent muscle atrophy and loss of function, particularly in highly susceptible muscle groups such as in the calf. Thus, additional interventions that complement inflight exercise countermeasures are sought. Testosterone has been considered as a potential countermeasure to be investigated due to its anabolic potential and known synergism with exercise. While exercise and testosterone are independently known to induce skeletal muscle protein synthesis, much is unknown regarding the differences and redundancies between the signals provided by the respective mechanical and biochemical stimuli.

The recent 70-day NASA-funded CFT70 bed rest campaign investigated the effects of strict, diet controlled, head down bed rest (HDBR) on lean body mass and muscle strength of healthy males, and the influence of a moderate to high-intensity exercise protocol (Sprint protocol), with or without testosterone supplementation, on mitigating these changes [2–4]. This NASA-led study was strictly monitored for all aspects known to affect skeletal muscle gain and loss (nutrition, exercise, axial loading, body movement etc.) and therefore provided a tremendously unique opportunity to investigate the effects of extended inactivity and unloading (with or without the inclusion of countermeasures) on changes in abundance and phosphorylation of skeletal muscle proteins in humans. We hypothesized that confinement to HDBR would alter the skeletal muscle proteome and that the inclusion of exercise alone or exercise with testosterone supplementation would each result in unique modifications of proteomic responses during HDBR. Furthermore, this unique opportunity afforded us the ability to perform post-hoc regression analyses to determine whether baseline proteomic data could be predictive of HDBR- or countermeasure-induced responses in muscle mass or strength.

## Methods

### Ethics

Subjects were recruited through the National Aeronautics and Space Administration (NASA) Human Research Program (HRP) testing facility at the Johnson Space Center (JSC) in Houston, TX. Screening, including the JSC Human Test Subject Facility physical examination and psychological evaluations were completed at NASA JSC. The study complied with the Declaration of Helsinki and was approved by The University of Texas Medical Branch (UTMB) Institutional Review Board (IRB) and by the NASA Committee for the Protection of Human Subjects (CPHS). Written informed consent was obtained from all subjects, and subjects were studied at the NASA Flight Analogs Research Unit (FARU) at UTMB. This research was conducted as part of a larger integrated NASA bed rest study campaign registered with ClinicalTrials.gov (NCT00891449).

### Subjects

**Fig 1:**
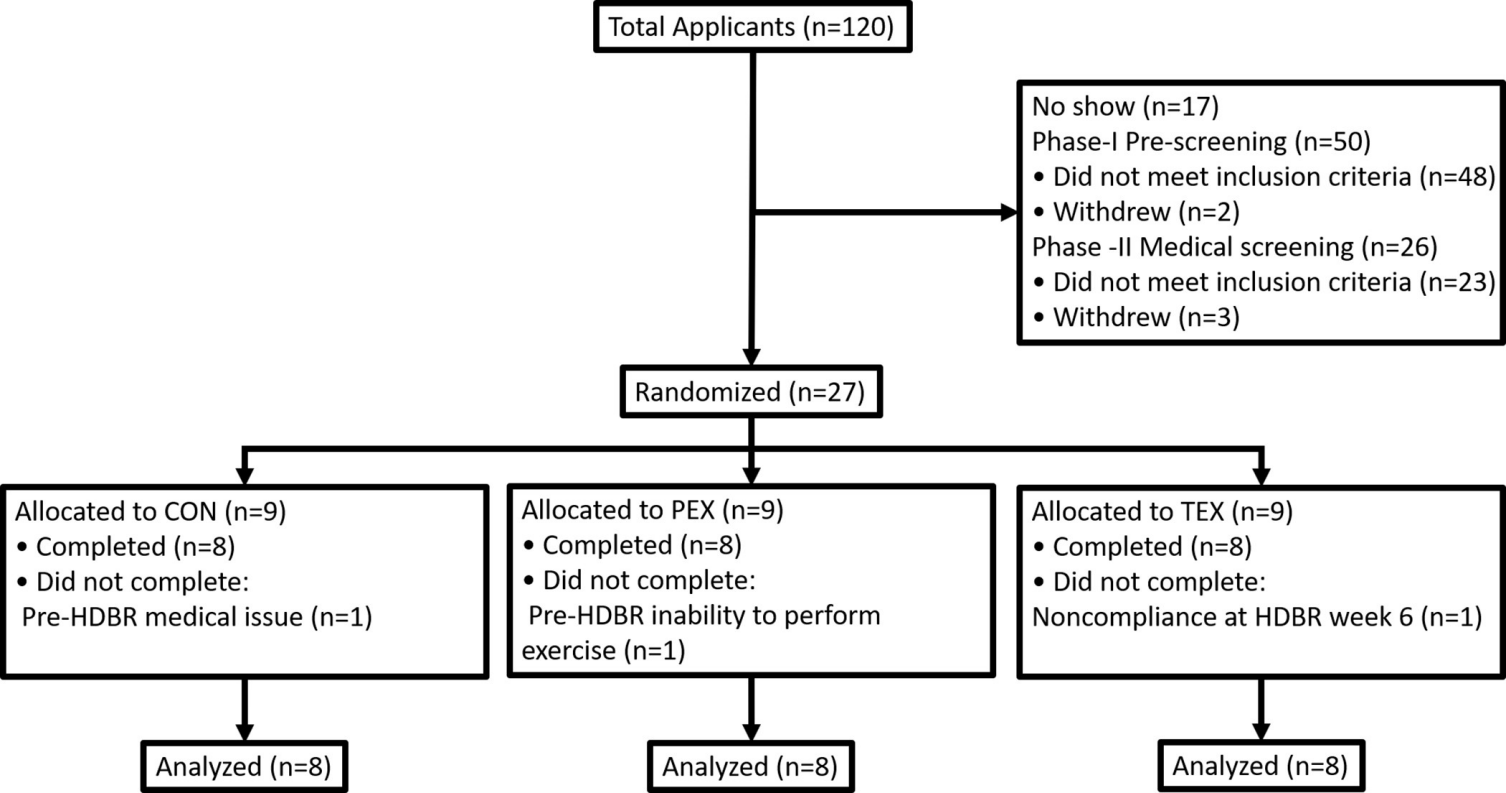
Subject flow diagram. This research was part of an integrated study registered with ClinicalTrials.gov (NCT00891449). Sample sizes were determined based on the primary outcomes from several independently funded investigations involved in the bed rest study campaign conducted between 2010 and 2014. A total of 24 subjects randomized to control (CON, n = 8), exercise plus placebo (PEX, n = 8) or exercise plus testosterone countermeasures (TEX, n = 8) completed this protocol during 70 days of head down bed rest (HDBR). Because of overlap in start-time between funded investigations, subject numbers may differ between reports that emanated from this bed rest campaign.

The bed rest study protocol and subject characteristics have been detailed in our previous report [3]. Study advertisement, recruitment, and randomization was conducted through the Human Test Subjects Facility at the NASA Johnson Space Center in Houston, TX. Healthy male volunteers (35 ± 8 years) were randomized (blocks of six) to one of 3 bed rest groups: placebo + non-exercise control (CON, n = 8), placebo + exercise (PEX, n = 8), and testosterone + exercise (TEX, n = 8) (**Fig 1**). Placebo vs. testosterone treatment assignments were blinded (CON) or double-blinded (PEX vs. TEX). The study was conducted at the Flight Analogs Research Unit (FARU) at UTMB in Galveston, TX. Testing was conducted at UTMB and NASA/JSC. PEX and TEX subjects followed a moderately intense exercise schedule throughout the HDBR phase [4]. Briefly, all PEX and TEX subjects followed an exercise protocol that included 6 days of high-intensity aerobic training, combined with 3 days of resistive strength exercise. Resistance exercise sessions were on the same day as the continuous aerobic exercise, separated by 4–6 h. Supine aerobic exercise was performed using the Standalone Zero Gravity Locomotion Simulator vertical treadmill and a supine cycle ergometer, and resistance exercise was performed on a horizontal squat device, a horizontal leg press (for leg press and calf raise exercise), and a prone leg curl machine. High-intensity interval aerobic exercise and continuous aerobic exercise were performed on alternating days. Starting one day before bed rest (BR-1), placebo (saline) or testosterone enanthate injections (100 mg, intramuscular) were administered in 2-week intervals (weekly testosterone enanthate for two weeks, followed by two weeks off, etc.) for the duration of the 70-day bed rest period. Thus, injections occurred immediately before bedrest (BR-1), and during bedrest (BR7, BR28, BR35, BR56, and BR63). Licensed nurses administered the IM injections in the *gluteus maximus*, alternating between sides of the body throughout the study. Clinical outcomes from this investigation were published previously and there were no adverse events in response or testosterone treatment [5].

### Muscle biopsy procedure

Muscle tissue was collected on BR-1 (pre), BR36 (mid), and BR64 (post). All biopsies were performed on the left leg of each subject. Each subject underwent the procedure three times during the study resulting in three biopsies forming a triangle between sites. The first site was approximately 10 cm proximal from the patella. The second site was approximately 4 cm proximal to the first site. The third site was between and approximately 3 cm lateral from the previous sites. Muscle biopsy procedures were performed as described elsewhere [6, 7]. Briefly, a site was marked on the *vastus lateralis* and cleaned with Betadine. Lidocaine (1%) was administered to the skin and deep muscle. An approximately 5 mm incision was made through the skin and fascia and a 5 mm Bergström needle was advanced into the muscle. While suction was applied, 100–200 mg of skeletal muscle tissue was collected by opening and closing the cutting window of the biopsy needle. The incision was sutured and covered with Bacitracin and steri-strips. Ice was applied to the site and ibuprofen was provided to the subject to alleviate soreness.

### Skeletal muscle proteomics

Proteomic analyses were performed by the UTMB Biomolecular Resource Facility (BRF). Protein abundances were determined in fractionated muscle extracts using a Biofluids Analytical Platform (BAP) [8–10]. These analyses were completed in one continuous effort once all the muscle samples had been collected. The BAP fractionation component combines Superdex S-75 size-exclusion chromatography (SEC) of biofluids with electronically triggered fraction collection to create protein and peptide pools for subsequent separation and analysis. Fractionated samples were subjected to 2D gel electrophoresis (2DE) and stained for phosphoproteins (Pro-Q Diamond dye, ThermoFisher Scientific) or total proteins (Sypro Ruby dye, ThermoFisher Scientific). Pro-Q Diamond selectively stains phosphoproteins in gels and thus provides a convenient method for determining relative phosphorylation of proteins between samples–which is our purpose here—though not for pinpointing the sites (residues) of phosphorylation. The gels were imaged, and then analyzed using SameSpots software (TotalLab, Newcastle upon Tyne, UK), first aligning the images to a selected reference image, and quantitatively comparing log-transformed spot intensities between the groups (CON, PEX, TEX; pre, mid, post). Proteins showing significant p-value (≤ 0.05 in t-tests) and |fold differences| (≥1.50) between the groups (CON, PEX, TEX) or time points (pre, mid, post) were identified by matrix-assisted laser desorption/ionization (MALDI) time-of-flight (TOF/TOF) mass spectroscopy (MS). This is a method used commonly for protein identification following 2DE analysis [11]. Lists of MS identified proteoforms were subjected to Ingenuity Pathway Analysis (IPA) to identify cell-signaling networks that responded to HDBR and/or countermeasures. Principal components analysis (PCA) was performed to characterize clustering in the three groups based on protein abundance and protein phosphorylation.

### Data and statistical analyses

Data analyses included components of treatment (CON, PEX, TEX) and time (pre, post). The mid timepoint was included during the initial spot selection for MS identification and the data are presented in **Tables 1–4**. However, the discussion will focus on pre-post changes to facilitate interpretation of the findings.

**Table 1:** Changes in protein abundances. Within-group changes of all identified spots. Ordering within the table is based on proteoform interpretation (i.e. intact, aggregate, fragment) and p-values (2-tailed, paired t-tests) of the pre to post changes in CON. P-values < 0.05 are shaded in yellow. Differences (%) within each comparison are shaded to indicate higher (red) or lower (blue) values relative to pre.

									CON: Mid vs. Pre		CON: Post vs. Pre		EX: Mid vs. Pre		EX: Post vs. Pre		TEX: Mid vs. Pre		TEX: Post vs. Pre	
TABLE 1	Protein Name	Symbol	Accession Number	pI	MW (kD)	UNIPROT MW (kd)	MS Protein Score	Proteoform Interpretation	P-Value	Diff. (%)	P-Value	Diff. (%)	P-Value	Diff. (%)	P-Value	Diff. (%)	P-Value	Diff. (%)	P-Value	Diff. (%)
metabolic	Adenylate kinase isoenzyme 1	AK1	P00568	9.61	24	22	178	INTACT	0.001	59.974	0.004	145.03	0.727	10.229	0.399	47.385	0.163	-26.902	0.130	68.469
metabolic	Heat shock protein beta-7	HSPB7	Q9UBY9	5.97	18	19	265	INTACT	0.020	-7.022	0.004	-21.175	0.571	4.724	0.047	9.858	0.995	-0.416	0.036	-20.085
transport	Carbonic anhydrase 3	CA3	P07451	5.27	28	30	38	INTACT	0.093	17.191	0.006	64.159	0.253	-14.058	0.238	-11.829	0.497	-11.237	0.482	8.895
Ca	Bestrophin-3	BEST3	F8VVX2	10.2	18	17	36	INTACT	0.918	1.814	0.007	71.635	0.344	-10.005	0.230	-35.237	0.530	23.813	0.829	-7.558
Ca contractile	Tropomyosin beta chain	TPM2	P07951	5.26	34	33	592	INTACT	0.277	13.529	0.010	55.081	0.963	-0.473	0.131	-24.622	0.322	-13.284	0.800	4.170
contractile	Myosin-2	MYH2	Q9UKX2	9.67	195	223	451	INTACT	0.557	-7.499	0.011	-67.284	0.501	15.787	0.556	10.373	0.430	-15.782	0.395	-20.017
Ca	Bestrophin-3	BEST3	F8VVX2	4.51	15	17	36	INTACT	0.063	34.890	0.011	62.836	0.784	12.622	0.843	1.338	0.882	-16.726	0.439	-37.644
structural	Ankyrin repeat domain-containing protein 2	ANKRD2	Q9GZV1	5.57	37	40	325	INTACT	0.047	-39.684	0.018	-56.656	0.477	8.655	0.081	32.353	0.134	18.456	0.078	25.168
transcription	Elongation factor 1-alpha 1	EEF1A1	P68104	9.56	53	50	58	INTACT	0.559	8.409	0.019	87.905	0.613	4.918	0.663	11.792	0.285	-22.551	0.305	23.348
transport	Hemoglobin subunit alpha	HBA1	P69905	9.71	17	15	282	INTACT	0.343	24.089	0.019	184.10	0.838	0.765	0.566	-14.978	0.952	-7.445	0.012	123.57
metabolic	Heat shock protein beta-1	HSPB1	P04792	5.34	26	23	270	INTACT	0.951	1.147	0.021	91.377	0.266	-21.389	0.923	5.150	0.482	-16.309	0.306	21.900
degradation	Tripartite motif-containing protein 72	TRIM72	Q6ZMU5	6.15	49	53	343	INTACT	0.425	-2.615	0.023	-13.380	0.213	19.753	0.153	11.280	0.388	7.455	0.791	-0.799
transport	Hemoglobin subunit beta	HBB	P68871	6.30	14	16	468	INTACT	0.667	10.124	0.031	112.43	0.749	6.978	0.716	-0.824	0.114	-40.203	0.298	41.607
contractile	Myosin-2	MYH2	Q9UKX2	9.59	200	223	417	INTACT	0.648	-4.298	0.035	-46.738	0.930	1.842	0.582	-4.187	0.881	-11.862	0.635	-23.619
contractile	Isoform 2 of Actin, gamma-enteric smooth muscle	ACTG2	P63267-2	6.12	38	42	181	INTACT	0.589	-5.966	0.036	-22.725	0.213	-13.162	0.349	-7.231	0.181	-11.152	0.841	2.550
metabolic	Dihydrolipoyl dehydrogenase, mitochondrial	DLD	E9PEX6	6.82	54	52	271	INTACT	0.695	-2.649	0.039	-27.562	0.638	4.489	0.322	-11.232	0.013	11.906	0.565	5.057
structural	Actinin, alpha 2, isoform CRA_b	ACTN2	B2RCS5	4.91	100	104	62	INTACT	0.632	-14.040	0.042	-94.054	0.872	-2.195	0.745	-8.746	0.114	34.274	0.792	-0.894
contractile	Actin, alpha skeletal muscle	ACTA1	P68133	4.95	42	42	642	INTACT	0.839	-4.678	0.074	-30.931	0.003	53.973	0.163	22.737	0.275	12.075	0.573	-6.547
transport	Hemoglobin subunit delta	HBD	P02042	8.76	14	16	297	INTACT	0.494	-3.928	0.077	88.845	0.932	-0.590	0.880	-0.367	0.401	-11.742	0.208	26.399
transport	Hemoglobin subunit alpha	HBA1	P69905	9.73	14	15	455	INTACT	0.316	13.294	0.108	54.162	0.544	-17.414	0.737	-1.063	0.542	-6.699	0.097	20.322
structural	Ankyrin repeat domain-containing protein 2	ANKRD2	Q9GZV1	5.46	37	40	288	INTACT	0.207	-18.536	0.109	-30.051	0.015	20.824	0.108	19.527	0.265	-13.319	0.164	13.295
Ca	Bestrophin-3	BEST3	F8VVX2	8.68	19	17	46	INTACT	0.579	20.153	0.142	56.028	0.028	62.469	0.528	11.082	0.044	-59.236	0.997	-7.796
structural	Isoform 5 of Myosin-binding protein C, slow-type	MYBPC1	Q00872-5	5.94	131	128	733	INTACT	0.186	17.231	0.146	-25.507	0.843	0.791	0.329	-13.199	0.042	30.194	0.323	18.923
contractile	Actin, alpha skeletal muscle	ACTA1	Q5T8M7	5.51	44	38	416	INTACT	0.952	0.179	0.243	-10.434	0.098	-16.613	0.029	-19.957	0.114	-8.800	0.188	-4.148
structural	Isoform 5 of Radixin	RDX	P35241-5	6.16	74	69	65	INTACT	0.840	-4.186	0.275	39.290	0.043	19.269	0.008	25.274	0.454	30.954	0.008	50.948
transport	Hemoglobin subunit beta	HBB	P68871	6.27	14	16	321	INTACT	0.535	-3.584	0.283	43.307	0.712	-14.745	0.534	-18.281	0.243	-19.456	0.275	37.422
Ca	Protein S100-A13	S100A13	Q99584	5.55	13	11	72	INTACT	0.353	54.063	0.308	30.116	0.422	44.903	0.141	55.994	0.471	-11.489	0.008	87.546
contractile	Myosin light chain 1/3, skeletal muscle isoform	MYL1	P05976	5.24	21	21	673	INTACT	0.809	-1.707	0.319	-13.318	0.305	7.972	0.409	4.897	0.341	-5.513	0.106	-9.445
structural	Ankyrin repeat domain-containing protein 2	ANKRD2	Q9GZV1	5.50	37	40	143	INTACT	0.325	-7.626	0.321	-12.151	0.296	10.300	0.250	20.423	0.012	29.500	0.099	21.342
Ca	Bestrophin-3	BEST3	F8VVX2	6.26	18	17	40	INTACT	0.767	-5.559	0.340	27.256	0.099	-16.789	0.368	-14.984	0.631	1.243	0.090	-34.953
metabolic	Creatine kinase M-type	CKM	P06732	6.95	41	43	983	INTACT	0.882	0.440	0.361	-7.476	0.241	-9.951	0.077	-9.531	0.700	1.895	0.885	-1.366
metabolic	Creatine kinase M-type	CKM	P06732	7.43	42	43	717	INTACT	0.644	-3.791	0.387	-11.375	0.670	-2.217	0.325	-5.465	0.746	5.274	0.937	-0.089
transport	Serum albumin	ALB	P02768	5.76	81	69	167	INTACT	0.918	-1.211	0.400	35.737	0.003	-20.865	0.102	-13.992	0.624	8.361	0.052	24.045
transport	Myoglobin	MB	P02144	5.51	17	17	57	INTACT	0.978	5.363	0.474	-9.961	0.744	-16.770	0.500	-17.267	0.005	-40.099	0.004	-53.808
contractile	Isoform MLC3 of Myosin light chain 1/3, skeletal muscle isoform	MYL1	P05976-2	4.92	22	21	98	INTACT	0.218	16.147	0.528	29.802	0.933	-0.148	0.186	-37.780	0.562	-15.066	0.049	-92.457
contractile	Myosin-1	MYH1	P12882	9.48	200	223	551	INTACT	0.952	-0.057	0.540	-6.049	0.997	5.274	0.523	-15.468	0.272	-17.639	0.708	-15.474
contractile	Actin, alpha skeletal muscle	ACTA1	P68133	5.42	37	42	525	INTACT	0.522	-7.899	0.548	-6.458	0.918	0.813	0.208	20.450	0.074	30.566	0.372	10.649
Ca contractile	Tropomyosin beta chain	TPM2	P07951	5.20	35	33	120	INTACT	0.599	5.012	0.591	-5.877	0.111	-40.878	0.015	-50.238	0.399	-13.347	0.032	-26.507
Ca contractile	Troponin C type 2 (Fast), isoform CRA_a	TNNC2	C9J7T9	4.59	18	16	328	INTACT	0.642	14.527	0.594	15.640	0.868	-1.177	0.473	-17.612	0.824	0.013	0.273	-18.883
metabolic	Succinate dehydrogenase [ubiquinone] flavoprotein subunit, mitochondrial	SDHA	D6RFM5	6.22	68	64	155	INTACT	0.785	5.171	0.600	-9.833	0.783	1.307	0.345	-12.345	0.567	18.575	0.349	9.239
Ca	Bestrophin-3	BEST3	F8VVX2	6.67	18	17	36	INTACT	0.246	-17.864	0.643	19.374	0.109	-41.598	0.685	-9.005	0.712	-5.156	0.178	-28.094
contractile	Actin, alpha skeletal muscle	ACTA1	Q5T8M7	5.84	42	38	362	INTACT	0.939	1.110	0.661	-1.566	0.029	-29.561	0.361	-8.812	0.326	-12.461	0.861	0.764
contractile	Myosin light chain 1/3, skeletal muscle isoform	MYL1	P05976	5.93	23	21	360	INTACT	0.362	-6.866	0.713	8.042	0.065	-34.360	0.549	-2.921	0.234	-10.932	0.615	-2.652
contractile	Isoform MLC3 of Myosin light chain 1/3, skeletal muscle isoform	MYL1	P05976-2	5.17	20	21	199	INTACT	0.893	2.231	0.734	-1.826	0.395	7.284	0.856	4.598	0.351	-9.690	0.153	-9.954
structural	Alpha-actinin-2	ACTN2	P35609	10.2	104	104	162	INTACT	0.862	0.766	0.738	8.605	0.658	-3.903	0.786	8.146	0.500	0.141	0.189	-33.998
Ca contractile	Isoform 4 of Tropomyosin alpha-1 chain	TPM1	P09493-4	5.71	32	33	572	INTACT	0.685	-4.241	0.758	22.837	0.353	-18.897	0.841	-2.851	0.462	-14.688	0.395	-22.715
metabolic	Isoform 2 of Very long-chain specific acyl-CoA dehydrogenase, mitochondrial	ACADVL	P49748-2	8.74	68	70	96	INTACT	0.426	37.394	0.789	30.595	0.420	38.918	0.003	43.275	0.573	-15.173	0.056	-43.753
glycolysis	Fructose-bisphosphate aldolase	ALDOA	H3BQN4	9.33	38	39	470	INTACT	0.056	16.485	0.798	2.631	0.407	-5.423	0.100	9.765	0.708	-1.715	0.536	6.644
metabolic	Creatine kinase M-type	CKM	P06732	7.13	40	43	1010	INTACT	0.087	12.563	0.807	-0.736	0.168	-9.625	0.282	-6.291	0.001	13.993	0.713	1.124
metabolic	Fructose-bisphosphate aldolase A	ALDOA	P04075	7.44	38	39	380	INTACT	0.841	-1.072	0.822	1.448	0.546	8.271	0.382	-18.878	0.785	2.113	0.112	-42.546
structural	Desmin	DES	P17661	5.37	53	54	1170	INTACT	0.564	7.166	0.826	2.144	0.686	-12.459	0.203	28.061	0.183	35.983	0.808	-4.190
structural	Desmin	DES	P17661	5.32	50	54	509	INTACT	0.869	-10.445	0.842	-0.409	0.880	6.754	0.035	56.153	0.229	20.552	0.241	18.764
structural	Isoform 2 of Myosin-binding protein C, slow-type	MYBPC1	Q00872-2	5.70	141	128	625	INTACT	0.854	-0.973	0.868	-7.483	0.259	-16.945	0.079	-17.626	0.547	-46.173	0.462	-66.339
contractile	Actin, alpha skeletal muscle	ACTA1	P68133	6.00	41	42	454	INTACT	0.800	3.267	0.920	1.965	0.025	-37.937	0.417	-7.988	0.169	-13.643	0.021	13.234
contractile	Myosin-7	MYH7	P12883	7.09	205	223	381	INTACT	0.126	17.051	0.937	6.753	0.264	10.577	0.623	5.165	0.875	1.846	0.019	-52.137
Ca	Bestrophin-3	BEST3	F8VVX2	4.93	100	17	39	AGG	0.106	-100.35	0.003	-155.76	0.629	-10.803	0.734	-13.913	0.008	47.153	0.491	-7.530
transport	Hemoglobin subunit alpha	HBA1	P69905	9.71	18	15	262	AGG	0.157	63.270	0.009	415.75	0.441	2.707	0.343	-23.911	0.739	-38.058	0.053	139.57
Ca	Bestrophin-3	BEST3	F8VVX2	8.35	98	17	39	AGG	0.382	20.881	0.012	79.331	0.257	36.695	0.186	15.099	0.001	-40.615	0.857	-3.641
transport	Hemoglobin subunit alpha	HBA1	P69905	9.60	28	15	314	AGG	0.010	30.814	0.040	54.520	0.821	-2.394	0.569	-7.323	0.659	-3.149	0.008	51.527
transport	Hemoglobin subunit alpha	HBA1	P69905	9.27	99	15	111	AGG	0.277	15.679	0.042	44.222	0.186	23.029	0.994	3.615	0.454	-18.138	0.301	23.053
Ca	Bestrophin-3	BEST3	F8VVX2	6.35	173	17	53	AGG	0.134	-22.543	0.062	-33.595	0.103	-23.564	0.195	-17.598	0.025	31.607	0.366	18.612
Ca	Bestrophin-3	BEST3	F8VVX2	4.73	20	17	34	AGG	0.266	28.664	0.068	54.273	0.680	8.872	0.705	-7.459	0.090	27.672	0.675	3.845
degradation	E3 ubiquitin-protein ligase listerin	LTN1	H7BYG8	5.14	126	91	44	AGG	0.833	-0.946	0.077	67.094	0.267	5.934	0.770	-0.629	0.841	6.157	0.074	22.611
unkknown	Putative BCoR-like protein 2	BCORP1	Q8N888	8.96	98	16	38	AGG	0.935	-2.249	0.086	26.192	0.085	29.614	0.782	9.203	0.290	-21.309	0.412	4.834
metabolic	Cytochrome b-c1 complex subunit Rieske, mitochondrial	UQCRFS1	P47985	6.40	53	30	114	AGG	0.553	-2.568	0.087	-11.196	0.687	2.829	0.479	5.080	0.092	5.508	0.200	4.522
transport	Hemoglobin subunit beta	HBB	P68871	6.19	28	16	157	AGG	0.680	-2.005	0.109	60.473	0.684	-7.793	0.473	-13.581	0.511	-8.955	0.261	33.918
contractile	Isoform 2 of Actin, gamma-enteric smooth muscle	ACTG2	P63267-2	4.75	65	42	112	AGG	0.362	10.744	0.215	55.922	0.006	-88.787	0.308	-35.754	0.622	5.977	0.273	-41.640
transcription	Ataxin-3	ATXN3	G3V3T0	8.62	19	11	37	AGG	0.971	7.140	0.221	68.754	0.054	56.045	0.893	-1.350	0.002	-65.779	0.346	-15.426
Ca contractile	Tropomyosin beta chain	TPM2	P07951	5.01	159	33	769	AGG	0.044	-35.390	0.254	-17.162	0.710	2.746	0.015	46.717	0.964	1.442	0.509	-4.394
contractile	Isoform 2 of Actin, gamma-enteric smooth muscle	ACTG2	P63267-2	4.92	141	42	78	AGG	0.845	-20.853	0.269	-74.239	0.912	-6.392	0.085	57.682	0.038	72.087	0.119	43.965
transport	Hemoglobin subunit beta	HBB	P68871	5.86	28	16	169	AGG	0.510	-51.417	0.442	25.138	0.944	-6.076	0.937	-3.527	0.058	25.631	0.280	######
Ca contractile	TNNT1 protein	TNNT1	Q3B759	5.31	28	23	113	AGG	0.335	-25.058	0.466	20.421	0.066	-33.933	0.153	-8.681	0.680	-6.696	0.166	20.004
contractile	Actin, alpha skeletal muscle	ACTA1	P68133	5.32	123	42	626	AGG	0.532	-16.642	0.495	-9.226	0.414	-25.874	0.067	-42.148	0.307	33.196	0.035	20.538
Ca contractile	Troponin T, fast skeletal muscle	TNNT3	H9KVA2	6.21	35	28	234	AGG	0.551	3.151	0.537	-5.074	0.014	-59.731	0.029	-14.901	0.471	-3.728	0.520	-4.376
metabolic	Isoform 2 of Glycogen phosphorylase, muscle form	PYGM	P11217-2	6.78	250	97	266	AGG	0.625	10.307	0.579	8.766	0.035	36.550	0.384	14.501	0.983	9.053	0.003	-48.413
Ca	Bestrophin-3	BEST3	F8VVX2	4.68	31	17	42	AGG	0.911	1.010	0.639	15.589	0.096	-42.988	0.125	-53.243	0.809	-0.123	0.971	-1.098
contractile	Myosin light chain 1/3, skeletal muscle isoform	MYL1	P05976	5.94	38	21	415	AGG	0.301	7.757	0.646	-5.955	0.065	-22.812	0.560	-6.973	0.484	8.737	0.923	-2.902
transcription	Ataxin-3	ATXN3	G3V3T0	7.14	18	11	41	AGG	0.353	-19.374	0.656	-1.152	0.154	55.507	0.004	55.089	0.896	0.544	0.610	-13.225
contractile	Isoform 2 of Actin, gamma-enteric smooth muscle	ACTG2	P63267-2	4.69	195	42	93	AGG	0.885	-3.023	0.846	1.722	0.974	-5.658	0.822	-1.582	0.640	19.791	0.358	31.037
Ca contractile	Isoform 4 of Tropomyosin alpha-1 chain	TPM1	P09493-4	4.98	127	33	562	AGG	0.890	-3.529	0.886	3.224	0.003	74.828	0.003	64.046	0.583	5.678	0.466	-9.164
metabolic	Mitochondrial inner membrane protein	IMMT	C9J406	5.75	87	73	148	AGG	0.353	-6.863	0.896	-0.931	0.792	-2.951	0.429	-4.939	0.023	-12.604	0.853	1.353
transport	Carbonic anhydrase 3	CA3	P07451	7.16	111	30	204	AGG	0.967	-7.339	0.931	-10.820	0.091	49.858	0.410	17.701	0.992	-7.544	0.857	-3.378
contractile	Isoform 2 of Actin, gamma-enteric smooth muscle	ACTG2	P63267-2	4.78	100	42	65	AGG	0.407	5.839	0.946	-3.859	0.061	-32.013	0.774	-6.043	0.187	38.621	0.406	24.586
contractile	Actin, alpha skeletal muscle	ACTA1	P68133	5.32	97	42	632	AGG	0.847	-2.549	0.969	5.319	0.121	-59.774	0.007	-61.271	0.525	17.744	0.011	24.509
structural	Keratin, type II cytoskeletal 2 epidermal	KRT2	P35908	5.90	29	65	235	FRAG	0.105	-33.779	0.010	-68.189	0.068	25.534	0.401	61.275	0.014	72.284	0.601	-11.301
metabolic	Short-chain specific acyl-CoA dehydrogenase, mitochondrial	ACADS	P16219	6.30	38	44	95	FRAG	0.119	-20.021	0.011	-47.653	0.624	-4.929	0.590	2.441	0.353	10.737	0.211	21.749
transport	Hemoglobin subunit alpha	HBA1	P69905	5.24	13	15	152	FRAG	0.836	4.416	0.011	40.645	0.243	-23.019	0.236	-22.989	0.315	-13.330	0.481	10.203
transport	Myosin-7	MYH7	P12883	9.27	99	223	119	FRAG	0.277	15.679	0.042	44.222	0.186	23.029	0.994	3.615	0.454	-18.138	0.301	23.053
metabolic	Calsequestrin-1	CASQ1	P31415	4.75	34	45	117	FRAG	0.134	18.064	0.047	57.142	0.430	19.514	0.930	6.253	0.057	-30.206	0.293	-15.611
structural	Nebulin	NEB	F8WCL5	9.35	113	773	112	FRAG	0.285	29.891	0.049	37.168	0.268	38.281	0.869	4.306	0.593	13.530	0.159	32.437
structural	Desmin	DES	P17661	5.13	37	54	254	FRAG	0.640	10.914	0.061	33.296	0.173	-28.680	0.980	2.587	0.238	-18.324	0.626	5.982
glycolysis	Enolase (Fragment)	ENO3	E5RGZ4	7.90	111	30	104	FRAG	0.887	-6.345	0.076	-37.105	0.112	31.686	0.670	-14.817	0.330	26.891	0.329	-31.928
transport	Hemoglobin subunit beta	HBB	P68871	6.28	13	16	374	FRAG	0.886	6.702	0.077	69.345	0.483	-19.381	0.771	2.695	0.138	-29.700	0.578	26.524
contractile	Myosin regulatory light chain 2, skeletal muscle isoform (Fragment)	MYLPF	H3BML9	6.86	18	13	54	FRAG	0.968	5.957	0.084	23.218	0.766	-4.935	0.635	7.572	0.311	-17.507	0.103	-27.369
structural	Unconventional myosin-XIX (Fragment)	MYO19	K7EMZ0	4.83	13	8	33	FRAG	0.453	11.383	0.087	46.201	0.488	-8.860	0.666	-2.953	0.983	6.363	0.508	14.896
transport	Hemoglobin subunit beta	HBB	P68871	4.13	13	16	423	FRAG	0.890	-7.386	0.094	110.30	0.240	-37.858	0.399	-15.232	0.716	27.964	0.803	16.778
contractile	Actin, alpha cardiac muscle 1	ACTC1	P68032	5.14	31	42	402	FRAG	0.894	19.072	0.107	66.675	0.419	-11.198	0.712	20.182	0.004	-78.646	0.717	-3.449
transcription	Keratin, type I cytoskeletal 10	KRT10	P13645	6.77	18	59	69	FRAG	0.466	-5.725	0.109	45.959	0.544	-15.433	0.597	-5.846	0.653	-9.658	0.098	-15.187
metabolic	Acyl-coenzyme A synthetase ACSM2B, mitochondrial (Fragment)	ACSM2B	H3BQ84	4.93	29	12	42	FRAG	0.044	-24.131	0.131	-31.352	0.965	-3.970	0.159	-37.024	0.185	30.108	0.773	8.325
transport	Hemoglobin subunit beta	HBB	P68871	4.16	13	16	261	FRAG	0.850	-0.625	0.141	73.879	0.106	-42.471	0.153	-21.963	0.925	15.889	0.495	19.139
contractile	Actin, alpha cardiac muscle 1	ACTC1	P68032	5.35	34	42	334	FRAG	0.104	32.048	0.182	34.174	0.593	-15.202	0.147	-27.973	0.545	-4.691	0.002	-32.503
transport	Myoglobin (Fragment)	MB	B0QYF8	9.37	16	16	164	FRAG	0.011	123.22	0.183	19.268	0.529	9.365	0.832	-8.550	0.629	-18.888	0.248	-31.431
unknown	Microtubule-actin cross-linking factor 1, isoforms 1/2/3/5 (Fragment)	MACF1	H0Y390	9.41	137	506	40	FRAG	0.117	19.416	0.184	29.488	0.052	20.698	0.272	14.966	0.178	-14.149	0.002	48.663
contractile	Myosin regulatory light chain 2, skeletal muscle isoform (Fragment)	MYLPF	H3BML9	5.17	16	13	621	FRAG	0.758	2.304	0.191	-14.022	0.364	9.847	0.880	0.257	0.597	-0.315	0.852	0.309
metabolic	Acyl-coenzyme A synthetase ACSM2B, mitochondrial (Fragment)	ACSM2B	H3BQ84	5.31	18	12	47	FRAG	0.811	-14.188	0.205	-20.103	0.366	-11.135	0.000	-39.328	0.313	-6.814	0.262	10.793
metabolic	Isoform 2 of Glycogen phosphorylase, muscle form	PYGM	P11217-2	5.98	28	97	155	FRAG	0.175	23.378	0.210	17.216	0.655	5.662	0.592	8.229	0.007	-69.859	0.506	-12.780
contractile	Myosin-1	MYH1	P12882	9.92	191	223	358	FRAG	0.221	-11.060	0.214	-19.219	0.496	5.161	0.743	-10.136	0.356	2.791	0.875	-4.025
contractile	Myosin regulatory light chain 2, skeletal muscle isoform (Fragment)	MYLPF	H3BN54	5.20	16	15	797	FRAG	0.943	-1.750	0.289	-9.329	0.236	26.980	0.850	-0.055	0.223	11.650	0.976	-2.807
structural	Keratin, type I cytoskeletal 10	KRT10	P13645	5.63	21	59	200	FRAG	0.886	-4.688	0.300	26.584	0.668	-17.821	0.172	-33.551	0.509	-6.357	0.671	6.536
contractile	Myosin regulatory light chain 2, skeletal muscle isoform (Fragment)	MYLPF	H3BML9	6.33	18	13	129	FRAG	0.265	22.324	0.300	12.639	0.515	-10.523	0.593	11.125	0.253	-15.755	0.277	-13.101
transport	Myoglobin (Fragment)	MB	F2Z337	4.14	16	9	107	FRAG	0.507	-7.698	0.301	-15.272	0.866	-2.988	0.688	-8.587	0.189	33.805	0.886	1.382
structural	Keratin, type I cytoskeletal 10	KRT10	P13645	5.22	14	59	250	FRAG	0.197	-14.400	0.326	-12.827	0.412	-4.456	0.833	4.158	0.142	-7.949	0.121	-9.987
contractile	Isoform 2 of Actin, gamma-enteric smooth muscle	ACTG2	P63267-2	5.38	36	42	171	FRAG	0.594	9.301	0.327	-12.032	0.217	-14.127	0.068	-27.710	0.279	-16.151	0.014	-32.629
structural	Keratin, type II cytoskeletal 6A	KRT6A	P02538	6.13	33	60	435	FRAG	0.484	-8.428	0.334	-12.101	0.949	0.855	0.232	11.988	0.790	-0.641	0.415	-12.316
metabolic	Pyruvate kinase (Fragment)	PKM	H3BTN5	8.16	57	53	528	FRAG	0.314	5.868	0.335	-12.819	0.019	16.637	0.088	41.052	0.046	17.824	0.072	-18.709
structural	Cofilin-1 (Fragment)	CFL1	E9PLJ3	6.04	17	9	66	FRAG	0.924	0.646	0.362	-7.083	0.745	6.323	0.094	22.069	0.862	0.001	0.085	-17.436
contractile	Myosin regulatory light chain 2, skeletal muscle isoform (Fragment)	MYLPF	H3BPK4	6.22	17	22	90	FRAG	0.536	6.965	0.389	13.416	0.561	-10.970	0.483	7.869	0.181	-24.561	0.678	-5.782
contractile	Myosin-2	MYH2	Q9UKX2	5.60	143	223	384	FRAG	0.213	16.716	0.448	14.754	0.706	-5.083	0.228	-21.723	0.387	13.554	0.286	20.638
structural	Keratin, type I cytoskeletal 9	KRT9	P35527	7.05	18	62	173	FRAG	0.270	-25.589	0.459	-30.517	0.249	38.229	0.005	62.927	0.346	-5.986	0.726	16.306
transport	Myoglobin (Fragment)	MB	F2Z337	4.22	31	9	74	FRAG	0.878	1.741	0.491	25.302	0.061	-44.487	0.620	-2.151	0.293	33.642	0.617	15.100
transport	Myoglobin (Fragment)	MB	F2Z337	4.75	16	9	115	FRAG	0.633	3.271	0.504	0.139	0.073	51.451	0.507	12.151	0.061	27.606	0.829	2.775
contractile	Myosin regulatory light chain 2, skeletal muscle isoform (Fragment)	MYLPF	H3BML9	6.36	18	13	280	FRAG	0.542	-4.968	0.526	16.596	0.454	-22.196	0.695	5.523	0.069	-27.513	0.508	-4.763
contractile	Myosin regulatory light chain 2, skeletal muscle isoform (Fragment)	MYLPF	H3BN54	5.17	37	15	662	FRAG	0.305	18.757	0.601	-12.798	0.195	26.187	0.854	4.732	0.433	-4.446	0.248	5.422
unknown	Serine/threonine-protein phosphatase 4 regulatory subunit 4	PPP4R4	Q6NUP7	7.00	18	99	32	FRAG	0.422	-12.066	0.603	31.295	0.731	19.620	0.281	38.654	0.210	-59.963	0.295	-44.620
transcription	Histidine protein methyltransferase 1 homolog	METTL18	O95568	4.89	20	42	38	FRAG	0.129	-55.293	0.641	-5.231	0.730	-14.733	0.885	-1.598	0.045	46.197	0.346	9.834
transport	Myoglobin (Fragment)	MB	F2Z337	4.89	17	9	112	FRAG	0.935	7.103	0.641	0.917	0.009	36.433	0.951	-3.017	0.148	12.042	0.999	-8.156
contractile	Myosin regulatory light chain 2, skeletal muscle isoform (Fragment)	MYLPF	H3BML9	6.10	16	13	64	FRAG	0.965	1.738	0.936	3.122	0.743	-6.538	0.718	10.729	0.473	-12.942	0.792	-0.488
transport	Fatty acid-binding protein, heart (Fragment)	FABP3	S4R371	5.99	14	15	574	FRAG	0.198	-22.190	0.953	3.971	0.174	8.128	0.420	-11.975	0.078	-13.499	0.465	-7.175
structural	Nebulin	NEB	F8WCL5	8.96	109	773	67	FRAG	0.791	-5.050	0.985	8.001	0.326	58.027	0.743	15.439	0.167	-19.911	0.895	-2.046
structural	Keratin, type II cytoskeletal 1	KRT1	P04264	4.87	31	66	41	FRAG	0.259	-17.766	0.985	5.204	0.081	30.037	0.491	15.385	0.920	-3.073	0.420	-14.843
unknown	#N/A	#N/A	#N/A	10.2	19	#N/A	#N/A	#N/A	0.588	-9.415	0.633	17.080	0.025	-62.364	0.039	-39.293	0.601	0.763	0.215	-28.756
									**CON**		**CON**		**EX**		**EX**		**TEX**		**TEX**	
								**Total**	**Mid vs. Pre**		**Post vs. Pre**		**Mid vs. Pre**		**Post vs. Pre**		**Mid vs. Pre**		**Post vs. Pre**	
							**INTACT**	55		3		17		7		6		6		9
							**AGG**	29		2		5		4		5		6		4
							**FRAG**	46		2		6		2		2		5		3
							**TOTAL**	130		7		28		13		13		17		16

**Table 2:** Changes in protein phosphorylation. Within-group changes of all identified spots. Ordering within the table is based on proteoform interpretation (i.e. intact, aggregate, fragment) and p-values (2-tailed, paired t-tests) of the pre to post changes in CON. P-values < 0.05 are shaded in yellow. Differences (%) within each comparison are shaded to indicate higher (red) or lower (blue) values relative to pre.

									CON: Mid vs. Pre		CON: Post vs. Pre		EX: Mid vs. Pre		EX: Post vs. Pre		TEX: Mid vs. Pre		TEX: Post vs. Pre	
TABLE 2	Protein Name	Symbol	Accession Number	pI	MW (kD)	UNIPROT MW (kd)	MS Protein Score	Proteoform Interpretation	P-Value	Diff. (%)	P-Value	Diff. (%)	P-Value	Diff. (%)	P-Value	Diff. (%)	P-Value	Diff. (%)	P-Value	Diff. (%)
transport	Serum albumin	ALB	P02768	5.76	81	69	167	INTACT	0.088	-16.377	0.002	-31.046	0.021	25.426	0.022	18.927	0.473	8.244	0.221	6.072
structural	Ankyrin repeat domain-containing protein 2	ANKRD2	Q9GZV1	5.57	37	40	325	INTACT	0.172	-18.960	0.003	-36.411	0.445	9.137	0.191	21.639	0.702	6.374	0.674	-0.568
structural	Isoform 2 of Myosin-binding protein C, slow-type	MYBPC1	Q00872-2	5.70	141	128	625	INTACT	0.232	-21.211	0.004	-65.499	0.778	2.954	0.399	-17.345	0.885	-0.849	0.795	-14.097
metabolic	Dihydrolipoyl dehydrogenase, mitochondrial	DLD	E9PEX6	6.82	54	52	271	INTACT	0.153	52.829	0.005	218.78	0.008	-71.616	0.089	-33.710	0.814	5.129	0.006	43.588
contractile	Actin, alpha skeletal muscle	ACTA1	Q5T8M7	5.84	42	38	362	INTACT	0.674	-1.895	0.008	-29.860	0.007	24.080	0.218	13.959	0.385	6.230	0.896	-2.664
Ca contractile	Isoform 4 of Tropomyosin alpha-1 chain	TPM1	P09493-4	5.71	32	33	572	INTACT	0.183	25.928	0.010	67.594	0.373	-7.904	0.262	-8.037	0.731	-25.334	0.342	15.594
transport	Carbonic anhydrase 3	CA3	P07451	5.27	28	30	38	INTACT	0.196	18.463	0.016	20.965	0.863	4.034	0.288	9.667	0.185	6.057	0.041	29.330
metabolic	Isoform 2 of Very long-chain specific acyl-CoA dehydrogenase, mitochondrial	ACADVL	P49748-2	8.74	68	70	96	INTACT	0.353	21.067	0.021	73.591	0.813	7.247	0.250	33.994	0.723	10.603	0.905	7.753
transport	Hemoglobin subunit alpha	HBA1	P69905	9.73	14	15	455	INTACT	0.838	1.024	0.021	72.832	0.475	-11.995	0.555	3.463	0.833	2.112	0.136	18.360
transport	Hemoglobin subunit beta	HBB	P68871	6.30	14	16	468	INTACT	0.109	21.358	0.028	123.45	0.484	-13.910	0.252	-17.658	0.102	-19.114	0.596	41.000
structural	Ankyrin repeat domain-containing protein 2	ANKRD2	Q9GZV1	5.50	37	40	143	INTACT	0.372	-9.830	0.051	-26.410	0.334	11.352	0.138	24.249	0.227	16.195	0.787	2.972
contractile	Actin, alpha skeletal muscle	ACTA1	P68133	6.00	41	42	454	INTACT	0.604	3.814	0.055	-21.468	0.265	10.738	0.226	13.879	0.858	0.702	0.188	-11.268
contractile	Myosin light chain 1/3, skeletal muscle isoform	MYL1	P05976	5.93	23	21	360	INTACT	0.230	14.509	0.058	43.123	0.022	-30.028	0.197	-53.165	0.125	-11.542	0.487	-7.254
transport	Hemoglobin subunit beta	HBB	P68871	6.27	14	16	321	INTACT	0.717	8.427	0.059	75.777	0.909	-6.750	0.320	8.646	0.490	-7.064	0.229	43.790
contractile	Actin, alpha skeletal muscle	ACTA1	P68133	5.42	37	42	525	INTACT	0.380	-15.755	0.062	-47.995	0.272	13.704	0.216	25.413	0.120	17.955	0.545	-2.598
structural	Ankyrin repeat domain-containing protein 2	ANKRD2	Q9GZV1	5.46	37	40	288	INTACT	0.479	-9.245	0.063	-40.259	0.097	15.277	0.118	27.472	0.352	8.164	0.925	2.871
contractile	Myosin-2	MYH2	Q9UKX2	9.67	195	223	451	INTACT	0.739	6.430	0.114	-35.182	0.222	51.786	0.013	37.336	0.053	-15.551	0.606	-6.407
metabolic	Adenylate kinase isoenzyme 1	AK1	P00568	9.61	24	22	178	INTACT	0.279	4.450	0.131	32.941	0.518	7.271	0.895	1.406	0.618	12.585	0.056	28.956
structural	Isoform 5 of Radixin	RDX	P35241-5	6.16	74	69	65	INTACT	0.449	10.794	0.132	48.955	0.419	13.037	0.079	26.030	0.546	8.795	0.694	35.279
metabolic	Heat shock protein beta-1	HSPB1	P04792	5.34	26	23	270	INTACT	0.344	-12.279	0.134	37.997	0.048	-33.508	0.837	-0.741	0.796	1.528	0.032	33.298
structural	Desmin	DES	P17661	5.32	50	54	509	INTACT	0.044	28.847	0.154	28.380	0.504	11.396	0.024	41.797	0.150	28.569	0.048	40.068
degradation	Tripartite motif-containing protein 72	TRIM72	Q6ZMU5	6.15	49	53	343	INTACT	0.340	-15.023	0.158	22.035	0.251	12.505	0.264	53.223	0.743	-1.363	0.528	9.833
transport	Hemoglobin subunit delta	HBD	P02042	8.76	14	16	297	INTACT	0.723	3.198	0.160	70.750	0.192	-17.710	0.023	-30.196	0.980	-5.420	0.104	25.740
metabolic	Creatine kinase M-type	CKM	P06732	7.13	40	43	1010	INTACT	0.104	10.247	0.164	-10.460	0.093	17.080	0.896	-1.438	0.125	-18.079	0.011	-57.303
structural	Desmin	DES	P17661	5.37	53	54	1170	INTACT	0.319	3.512	0.184	-19.519	0.420	8.405	0.740	2.925	0.954	3.150	0.190	-11.537
metabolic	Creatine kinase M-type	CKM	P06732	6.95	41	43	983	INTACT	0.191	-6.565	0.205	-9.055	0.805	2.641	0.081	-9.345	0.063	-19.369	0.006	-55.851
contractile	Myosin-2	MYH2	Q9UKX2	9.59	200	223	417	INTACT	0.631	2.829	0.230	-23.686	0.458	35.924	0.036	39.686	0.059	-12.888	0.171	-22.045
contractile	Actin, alpha skeletal muscle	ACTA1	Q5T8M7	5.51	44	38	416	INTACT	0.615	5.951	0.236	-23.175	0.228	18.554	0.815	1.339	0.480	4.657	0.391	-19.210
contractile	Isoform MLC3 of Myosin light chain 1/3, skeletal muscle isoform	MYL1	P05976-2	4.92	22	21	98	INTACT	0.342	-9.033	0.236	-13.189	0.497	10.302	0.335	15.418	0.977	-3.022	0.932	0.973
contractile	Isoform MLC3 of Myosin light chain 1/3, skeletal muscle isoform	MYL1	P05976-2	5.17	20	21	199	INTACT	0.082	23.429	0.238	7.781	0.978	-4.331	0.771	-0.439	0.370	-6.626	0.276	-11.943
contractile	Myosin-1	MYH1	P12882	9.48	200	223	551	INTACT	0.690	-4.372	0.247	-15.043	0.360	31.637	0.075	44.326	0.192	-11.514	0.147	-14.749
Ca	Bestrophin-3	BEST3	F8VVX2	6.67	18	17	36	INTACT	0.421	16.006	0.272	40.554	0.056	-87.545	0.293	-40.258	0.030	-42.169	0.161	-40.087
structural	Actinin, alpha 2, isoform CRA_b	ACTN2	B2RCS5	4.91	100	104	62	INTACT	0.544	-7.832	0.273	70.093	0.521	-12.846	0.123	-30.903	0.231	-10.466	0.323	18.221
Ca contractile	Tropomyosin beta chain	TPM2	P07951	5.26	34	33	592	INTACT	0.225	-11.486	0.280	35.618	0.557	-5.287	0.631	-3.109	0.091	-15.055	0.499	19.765
contractile	Actin, alpha skeletal muscle	ACTA1	P68133	4.95	42	42	642	INTACT	0.655	-5.770	0.289	-21.530	0.666	-5.218	0.910	3.015	0.018	21.377	0.254	23.030
glycolysis	Fructose-bisphosphate aldolase	ALDOA	H3BQN4	9.33	38	39	470	INTACT	0.753	0.156	0.294	14.020	0.554	-6.965	0.416	-10.814	0.065	20.680	0.017	54.191
transport	Hemoglobin subunit alpha	HBA1	P69905	9.71	17	15	282	INTACT	0.533	8.613	0.348	20.792	0.551	-11.979	0.538	-7.798	0.853	1.860	0.570	7.385
contractile	Isoform 2 of Actin, gamma-enteric smooth muscle	ACTG2	P63267-2	6.12	38	42	181	INTACT	0.418	-11.161	0.384	-15.655	0.086	17.568	0.010	48.739	0.261	8.075	0.010	31.764
contractile	Myosin light chain 1/3, skeletal muscle isoform	MYL1	P05976	5.24	21	21	673	INTACT	0.266	-11.106	0.405	-4.221	0.014	18.968	0.078	20.512	0.175	-13.107	0.698	-2.580
transcription	Elongation factor 1-alpha 1	EEF1A1	P68104	9.56	53	50	58	INTACT	0.749	3.233	0.420	43.295	0.625	-12.507	0.328	-18.533	0.613	19.422	0.226	28.387
metabolic	Creatine kinase M-type	CKM	P06732	7.43	42	43	717	INTACT	0.682	-7.129	0.483	-12.718	0.112	-17.283	0.047	-20.348	0.732	-4.286	0.008	-57.083
structural	Alpha-actinin-2	ACTN2	P35609	10.2	104	104	162	INTACT	0.785	2.241	0.484	-7.086	0.487	13.078	0.684	6.734	0.507	21.551	0.021	77.826
contractile	Myosin-7	MYH7	P12883	7.09	205	223	381	INTACT	0.085	16.181	0.493	14.459	0.051	34.110	0.215	33.495	0.766	2.589	0.945	5.783
structural	Isoform 5 of Myosin-binding protein C, slow-type	MYBPC1	Q00872-5	5.94	131	128	733	INTACT	0.658	6.238	0.513	-11.562	0.555	-5.429	0.358	7.851	0.195	-9.493	0.725	7.892
Ca contractile	Tropomyosin beta chain	TPM2	P07951	5.20	35	33	120	INTACT	0.149	-40.841	0.559	-18.088	0.838	7.614	0.183	-28.029	0.196	22.866	0.068	-35.092
Ca	Bestrophin-3	BEST3	F8VVX2	6.26	18	17	40	INTACT	0.057	-66.450	0.569	4.364	0.057	-46.859	0.248	-18.825	0.229	-31.220	0.115	-30.671
Ca	Protein S100-A13	S100A13	Q99584	5.55	13	11	72	INTACT	0.057	-87.694	0.577	-19.353	0.838	10.193	0.968	-2.678	0.518	17.630	0.031	35.661
Ca	Bestrophin-3	BEST3	F8VVX2	10.2	18	17	36	INTACT	0.907	7.117	0.600	20.084	0.527	3.366	0.811	-15.321	0.338	24.510	0.452	15.752
Ca contractile	Troponin C type 2 (Fast), isoform CRA_a	TNNC2	C9J7T9	4.59	18	16	328	INTACT	0.397	19.862	0.639	3.340	0.296	18.049	0.083	19.016	0.297	-17.195	0.001	-50.171
Ca	Bestrophin-3	BEST3	F8VVX2	4.51	15	17	36	INTACT	0.619	0.778	0.673	1.644	0.624	3.669	0.620	3.489	0.146	7.461	0.017	22.379
transport	Myoglobin	MB	P02144	5.51	17	17	57	INTACT	0.932	1.814	0.687	11.851	0.519	-19.525	0.867	-17.269	0.188	-18.921	0.227	-26.890
Ca	Bestrophin-3	BEST3	F8VVX2	8.68	19	17	46	INTACT	0.955	-4.365	0.707	20.252	0.883	-5.957	0.230	-37.166	0.726	-30.286	0.803	-33.192
metabolic	Heat shock protein beta-7	HSPB7	Q9UBY9	5.97	18	19	265	INTACT	0.230	19.379	0.741	10.231	0.195	-33.663	0.702	-19.585	0.081	-31.832	0.019	-54.451
metabolic	Fructose-bisphosphate aldolase A	ALDOA	P04075	7.44	38	39	380	INTACT	0.282	-6.333	0.854	-4.964	0.650	-3.678	0.050	-23.399	0.745	-3.947	0.016	-68.130
metabolic	Succinate dehydrogenase [ubiquinone] flavoprotein subunit, mitochondrial	SDHA	D6RFM5	6.22	68	64	155	INTACT	0.157	-56.986	0.866	-2.947	0.245	32.462	0.072	63.241	0.050	22.634	0.017	73.782
contractile	Myosin light chain 1/3, skeletal muscle isoform	MYL1	P05976	5.94	38	21	415	AGG	0.091	-25.492	0.014	-41.151	0.172	31.383	0.229	13.347	0.545	-3.836	0.331	11.836
Ca contractile	Isoform 4 of Tropomyosin alpha-1 chain	TPM1	P09493-4	4.98	127	33	562	AGG	0.639	12.663	0.038	38.043	0.089	46.624	0.031	69.072	0.548	-6.232	0.605	9.914
transport	Hemoglobin subunit alpha	HBA1	P69905	9.27	99	15	111	AGG	0.457	34.506	0.075	92.095	0.727	-14.350	0.420	-29.088	0.597	12.895	0.058	43.132
Ca	Bestrophin-3	BEST3	F8VVX2	8.35	98	17	39	AGG	0.339	16.525	0.078	45.482	0.527	-15.922	0.295	-30.296	0.293	-13.917	0.835	11.958
transport	Carbonic anhydrase 3	CA3	P07451	7.16	111	30	204	AGG	0.693	21.650	0.092	72.191	0.446	-17.725	0.456	-19.421	0.246	19.841	0.022	48.588
metabolic	Cytochrome b-c1 complex subunit Rieske, mitochondrial	UQCRFS1	P47985	6.40	53	30	114	AGG	0.138	19.466	0.117	27.956	0.508	3.635	0.360	5.698	0.053	16.997	0.017	18.384
contractile	Isoform 2 of Actin, gamma-enteric smooth muscle	ACTG2	P63267-2	4.69	195	42	93	AGG	0.130	-56.075	0.138	34.406	0.337	45.939	0.359	26.428	0.119	36.950	0.017	75.663
Ca contractile	Troponin T, fast skeletal muscle	TNNT3	H9KVA2	6.21	35	28	234	AGG	0.879	0.463	0.142	-21.683	0.681	-3.007	0.864	1.513	0.503	9.799	0.471	-6.220
Ca	Bestrophin-3	BEST3	F8VVX2	4.73	20	17	34	AGG	0.900	-2.481	0.174	28.277	0.350	17.663	0.716	4.050	0.262	15.046	0.449	11.325
transport	Hemoglobin subunit alpha	HBA1	P69905	9.71	18	15	262	AGG	0.558	8.003	0.183	18.206	0.834	-5.019	0.066	23.395	0.657	-8.004	0.465	11.350
Ca contractile	TNNT1 protein	TNNT1	Q3B759	5.31	28	23	113	AGG	0.374	6.084	0.197	-14.342	0.706	7.487	0.048	20.841	0.285	7.194	0.121	11.286
Ca	Bestrophin-3	BEST3	F8VVX2	4.93	100	17	39	AGG	0.035	-83.244	0.222	-42.503	0.248	15.770	0.971	7.077	0.204	-31.210	0.436	5.503
transcription	Ataxin-3	ATXN3	G3V3T0	8.62	19	11	37	AGG	0.501	16.650	0.289	34.968	0.686	-25.806	0.689	-11.199	0.204	-48.190	0.281	-31.961
transport	Hemoglobin subunit beta	HBB	P68871	6.19	28	16	157	AGG	0.348	21.979	0.336	20.300	0.565	17.868	0.054	-22.384	0.323	-8.531	0.212	10.734
transport	Hemoglobin subunit beta	HBB	P68871	5.86	28	16	169	AGG	0.034	-35.599	0.369	-1.744	0.327	27.851	0.226	23.574	0.840	-0.431	0.532	17.996
Ca contractile	Tropomyosin beta chain	TPM2	P07951	5.01	159	33	769	AGG	0.904	-5.087	0.476	2.766	0.698	-7.272	0.502	-8.195	0.343	-10.860	0.993	8.709
contractile	Actin, alpha skeletal muscle	ACTA1	P68133	5.32	123	42	626	AGG	0.810	-0.928	0.518	-13.951	0.477	-10.261	0.999	3.879	0.336	-19.552	0.257	12.758
transcription	Ataxin-3	ATXN3	G3V3T0	7.14	18	11	41	AGG	0.329	-21.376	0.522	3.707	0.384	-23.430	0.809	-7.606	0.208	-17.320	0.068	-43.067
metabolic	Mitochondrial inner membrane protein	IMMT	C9J406	5.75	87	73	148	AGG	0.785	-7.492	0.621	-28.581	0.309	19.853	0.024	26.239	0.988	4.047	0.264	-7.117
contractile	Isoform 2 of Actin, gamma-enteric smooth muscle	ACTG2	P63267-2	4.78	100	42	65	AGG	0.251	22.075	0.636	19.697	0.008	-38.309	0.331	10.892	0.022	52.418	0.020	33.330
transport	Hemoglobin subunit alpha	HBA1	P69905	9.60	28	15	314	AGG	0.996	0.397	0.641	5.530	0.351	-11.447	0.624	-4.382	0.672	11.715	0.487	-5.292
unkknown	Putative BCoR-like protein 2	BCORP1	Q8N888	8.96	98	16	38	AGG	0.984	-2.929	0.659	15.608	0.149	-32.953	0.475	-5.274	0.665	0.041	0.079	24.625
contractile	Isoform 2 of Actin, gamma-enteric smooth muscle	ACTG2	P63267-2	4.75	65	42	112	AGG	0.989	-4.139	0.792	14.342	0.286	-25.358	0.495	10.230	0.142	59.503	0.050	39.371
degradation	E3 ubiquitin-protein ligase listerin	LTN1	H7BYG8	5.14	126	91	44	AGG	0.237	-17.638	0.805	-2.634	0.561	3.976	0.637	-3.253	0.044	19.805	0.154	16.633
Ca	Bestrophin-3	BEST3	F8VVX2	6.35	173	17	53	AGG	0.293	26.579	0.835	9.887	0.367	-24.147	0.375	-20.227	0.587	-2.237	0.079	20.672
contractile	Isoform 2 of Actin, gamma-enteric smooth muscle	ACTG2	P63267-2	4.92	141	42	78	AGG	0.159	-55.460	0.845	-17.202	0.937	5.064	0.168	28.258	0.740	3.499	0.377	23.702
metabolic	Isoform 2 of Glycogen phosphorylase, muscle form	PYGM	P11217-2	6.78	250	97	266	AGG	0.958	-10.494	0.882	-8.007	0.114	22.257	0.779	1.048	0.080	-19.479	0.505	-5.476
Ca	Bestrophin-3	BEST3	F8VVX2	4.68	31	17	42	AGG	0.419	-24.676	0.884	-10.046	0.180	-34.873	0.056	-31.471	0.706	0.260	0.728	-2.315
contractile	Actin, alpha skeletal muscle	ACTA1	P68133	5.32	97	42	632	AGG	0.136	36.669	0.894	-6.617	0.139	-21.983	0.628	-5.594	0.370	16.156	0.517	13.842
structural	Nebulin	NEB	F8WCL5	9.35	113	773	112	FRAG	0.545	24.170	0.007	97.004	0.525	12.149	0.339	-16.293	0.829	1.738	0.327	23.878
transport	Fatty acid-binding protein, heart (Fragment)	FABP3	S4R371	5.99	14	15	574	FRAG	0.427	10.456	0.013	66.293	0.888	-1.004	0.683	-2.839	0.610	-5.919	0.225	33.056
contractile	Actin, alpha cardiac muscle 1	ACTC1	P68032	5.14	31	42	402	FRAG	0.616	-8.374	0.013	66.647	0.416	-12.373	0.898	9.155	0.219	-18.341	0.244	21.654
contractile	Myosin regulatory light chain 2, skeletal muscle isoform (Fragment)	MYLPF	H3BN54	5.20	16	15	797	FRAG	0.580	-15.458	0.021	-63.103	0.404	16.359	0.600	-13.293	0.486	9.229	0.024	-34.595
contractile	Myosin-2	MYH2	Q9UKX2	5.60	143	223	384	FRAG	0.759	-5.655	0.022	-53.665	0.875	0.954	0.008	-20.826	0.393	16.657	0.345	4.816
unknown	Serine/threonine-protein phosphatase 4 regulatory subunit 4	PPP4R4	Q6NUP7	7.00	18	99	32	FRAG	0.337	21.480	0.022	49.030	0.402	-42.126	0.490	-36.055	0.014	-73.364	0.007	-77.302
transport	Hemoglobin subunit alpha	HBA1	P69905	5.24	13	15	152	FRAG	0.050	20.765	0.026	31.505	0.451	-4.047	0.132	-8.209	0.920	-0.373	0.868	3.284
structural	Keratin, type I cytoskeletal 9	KRT9	P35527	7.05	18	62	173	FRAG	0.373	12.484	0.026	37.369	0.334	-43.696	0.848	-17.835	0.003	-69.265	0.015	-90.232
glycolysis	Enolase (Fragment)	ENO3	E5RGZ4	7.90	111	30	104	FRAG	0.493	-12.362	0.027	47.906	0.300	28.137	0.600	-18.914	0.551	2.809	0.150	42.410
contractile	Myosin regulatory light chain 2, skeletal muscle isoform (Fragment)	MYLPF	H3BML9	5.17	16	13	621	FRAG	0.800	4.657	0.046	-25.100	0.029	38.986	0.738	4.163	0.857	3.329	0.076	-27.338
contractile	Myosin regulatory light chain 2, skeletal muscle isoform (Fragment)	MYLPF	H3BML9	6.86	18	13	54	FRAG	0.632	11.869	0.049	64.910	0.357	-66.069	0.521	-51.517	0.037	-62.721	0.184	-51.561
transcription	Histidine protein methyltransferase 1 homolog	METTL18	O95568	4.89	20	42	38	FRAG	0.696	23.242	0.069	98.219	0.467	7.996	0.860	-2.406	0.152	27.597	0.043	22.357
transport	Myosin-7	MYH7	P12883	9.27	99	223	119	FRAG	0.457	34.506	0.075	92.095	0.727	-14.350	0.420	-29.088	0.597	12.895	0.058	43.132
transcription	Keratin, type I cytoskeletal 10	KRT10	P13645	6.77	18	59	69	FRAG	0.328	18.298	0.095	60.439	0.091	######	0.450	-62.068	0.122	-50.316	0.154	-53.174
metabolic	Short-chain specific acyl-CoA dehydrogenase, mitochondrial	ACADS	P16219	6.30	38	44	95	FRAG	0.417	-11.555	0.097	-22.539	0.323	9.613	0.631	2.673	0.602	7.540	0.620	-8.344
structural	Keratin, type II cytoskeletal 6A	KRT6A	P02538	6.13	33	60	435	FRAG	0.020	-25.855	0.101	-32.417	0.197	6.726	0.143	8.603	0.227	6.486	0.670	3.364
structural	Keratin, type I cytoskeletal 10	KRT10	P13645	5.22	14	59	250	FRAG	0.934	-1.998	0.116	-20.881	0.210	34.289	0.009	42.113	0.138	24.580	0.445	13.732
metabolic	Isoform 2 of Glycogen phosphorylase, muscle form	PYGM	P11217-2	5.98	28	97	155	FRAG	0.324	-31.697	0.125	-40.609	0.548	3.120	0.189	16.016	0.125	-27.728	0.670	8.776
transport	Hemoglobin subunit beta	HBB	P68871	6.28	13	16	374	FRAG	0.849	-1.401	0.125	70.949	0.264	-15.643	0.341	-15.074	0.154	-21.239	0.149	46.085
contractile	Myosin-1	MYH1	P12882	9.92	191	223	358	FRAG	0.609	-6.091	0.126	-31.534	0.054	43.161	0.105	39.252	0.845	2.181	0.885	3.248
transport	Hemoglobin subunit beta	HBB	P68871	4.13	13	16	423	FRAG	0.382	14.695	0.128	22.979	0.130	-32.324	0.256	-19.558	0.001	58.875	0.299	19.154
structural	Keratin, type II cytoskeletal 1	KRT1	P04264	4.87	31	66	41	FRAG	0.349	16.586	0.140	100.12	0.197	-33.032	0.472	-16.206	0.038	-41.007	0.317	-15.130
contractile	Myosin regulatory light chain 2, skeletal muscle isoform (Fragment)	MYLPF	H3BML9	6.36	18	13	280	FRAG	0.145	20.827	0.184	28.320	0.225	-50.349	0.988	-15.087	0.121	-28.392	0.253	-29.172
transport	Hemoglobin subunit beta	HBB	P68871	4.16	13	16	261	FRAG	0.423	10.065	0.193	38.468	0.029	-36.403	0.319	-21.181	0.364	21.832	0.176	21.477
transport	Myoglobin (Fragment)	MB	F2Z337	4.89	17	9	112	FRAG	0.032	48.747	0.201	36.641	0.491	-20.197	0.224	-28.763	0.819	-8.808	0.529	-12.923
contractile	Actin, alpha cardiac muscle 1	ACTC1	P68032	5.35	34	42	334	FRAG	0.300	-12.549	0.236	15.391	0.709	9.240	0.490	-5.697	0.384	-5.801	0.090	-26.145
contractile	Myosin regulatory light chain 2, skeletal muscle isoform (Fragment)	MYLPF	H3BML9	6.10	16	13	64	FRAG	0.233	22.680	0.258	27.140	0.418	-39.179	0.654	-6.463	0.418	-8.783	0.316	-24.070
metabolic	Acyl-coenzyme A synthetase ACSM2B, mitochondrial (Fragment)	ACSM2B	H3BQ84	5.31	18	12	47	FRAG	0.720	-15.501	0.271	-36.934	0.343	13.249	0.430	7.399	0.646	-6.673	0.154	-14.364
transport	Myoglobin (Fragment)	MB	F2Z337	4.75	16	9	115	FRAG	0.170	37.563	0.330	37.392	0.442	-19.641	0.039	-35.987	0.394	-22.248	0.064	-48.865
transport	Myoglobin (Fragment)	MB	F2Z337	4.14	16	9	107	FRAG	0.550	6.080	0.437	15.893	0.378	-29.285	0.905	3.866	0.798	12.402	0.052	-28.304
contractile	Myosin regulatory light chain 2, skeletal muscle isoform (Fragment)	MYLPF	H3BN54	5.17	37	15	662	FRAG	0.905	-7.540	0.457	-0.369	0.119	10.644	0.435	11.671	0.790	7.402	0.420	17.681
metabolic	Calsequestrin-1	CASQ1	P31415	4.75	34	45	117	FRAG	0.818	3.327	0.515	12.230	0.086	16.615	0.922	0.317	0.682	3.102	0.141	17.560
contractile	Myosin regulatory light chain 2, skeletal muscle isoform (Fragment)	MYLPF	H3BML9	6.33	18	13	129	FRAG	0.015	32.227	0.527	16.578	0.355	-27.585	0.763	-15.196	0.187	-16.661	0.304	-25.529
contractile	Myosin regulatory light chain 2, skeletal muscle isoform (Fragment)	MYLPF	H3BPK4	6.22	17	22	90	FRAG	0.703	5.638	0.548	0.771	0.240	-48.094	0.798	-10.036	0.308	-23.879	0.663	-17.519
structural	Cofilin-1 (Fragment)	CFL1	E9PLJ3	6.04	17	9	66	FRAG	0.351	14.784	0.576	9.863	0.090	-48.829	0.877	-13.256	0.106	-33.426	0.052	-41.203
metabolic	Acyl-coenzyme A synthetase ACSM2B, mitochondrial (Fragment)	ACSM2B	H3BQ84	4.93	29	12	42	FRAG	0.667	3.979	0.582	11.406	0.897	2.847	0.546	-9.938	0.370	-4.131	0.863	-1.452
transport	Myoglobin (Fragment)	MB	B0QYF8	9.37	16	16	164	FRAG	0.936	1.008	0.604	16.059	0.046	-45.512	0.032	-22.197	0.334	15.562	0.197	22.545
unknown	Microtubule-actin cross-linking factor 1, isoforms 1/2/3/5 (Fragment)	MACF1	H0Y390	9.41	137	506	40	FRAG	0.620	-4.088	0.628	5.432	0.346	17.411	0.601	14.243	0.357	-11.605	0.026	26.817
transport	Myoglobin (Fragment)	MB	F2Z337	4.22	31	9	74	FRAG	0.995	-6.011	0.668	14.068	0.027	-59.994	0.225	-18.566	0.445	28.736	0.625	6.979
structural	Desmin	DES	P17661	5.13	37	54	254	FRAG	0.495	9.080	0.710	-8.268	0.748	6.110	0.923	-2.209	0.809	-0.115	0.110	-37.263
structural	Unconventional myosin-XIX (Fragment)	MYO19	K7EMZ0	4.83	13	8	33	FRAG	0.997	-1.975	0.712	2.683	0.395	-26.914	0.105	-39.817	0.937	-0.938	0.661	5.539
structural	Nebulin	NEB	F8WCL5	8.96	109	773	67	FRAG	0.251	-16.458	0.725	0.249	0.798	-6.351	0.930	-0.511	0.042	-24.959	0.152	12.266
structural	Keratin, type II cytoskeletal 2 epidermal	KRT2	P35908	5.90	29	65	235	FRAG	0.030	-39.062	0.756	1.992	0.047	-24.031	0.016	-38.113	0.985	-2.752	0.996	1.694
metabolic	Pyruvate kinase (Fragment)	PKM	H3BTN5	8.16	57	53	528	FRAG	0.127	-13.560	0.760	-1.189	0.554	-6.602	0.078	-11.098	0.800	2.887	0.528	7.900
structural	Keratin, type I cytoskeletal 10	KRT10	P13645	5.63	21	59	200	FRAG	0.136	-23.353	0.958	2.664	0.539	-12.350	0.367	-14.524	0.500	8.158	0.159	27.849
contractile	Isoform 2 of Actin, gamma-enteric smooth muscle	ACTG2	P63267-2	5.38	36	42	171	FRAG	0.470	-4.668	0.992	-0.574	0.973	13.280	0.171	-18.861	0.050	-19.235	0.055	-50.668
unknown	#N/A	#N/A	#N/A	10.2	19	#N/A	#N/A	#N/A	0.910	-4.952	0.893	2.706	0.169	-28.707	0.863	-3.407	0.691	-16.698	0.841	-18.142
									**CON**		**CON**		**EX**		**EX**		**TEX**		**TEX**	
								**Total**	**Mid vs. Pre**		**Post vs. Pre**		**Mid vs. Pre**		**Post vs. Pre**		**Mid vs. Pre**		**Post vs. Pre**	
							**INTACT**	55		1		10		6		7		2		16
							**AGG**	29		2		2		1		3		2		4
							**FRAG**	46		5		11		5		5		7		5
							**TOTAL**	130		8		23		12		15		11		25

**Table 3:** Protein abundance differences in PEX vs TEX. Differences between PEX and TEX in all identified spots. Ordering within the table is based on proteoform interpretation (i.e. intact, aggregate, fragment) and p-values (2-tailed, unpaired t-tests) of the post comparison between PEX and TEX. P-values < 0.05 are shaded in yellow. Differences (%) within each comparison are shaded to indicate higher (red) or lower (blue) values in PEX relative to TEX.

									pre		mid		post	
									PEX compared to TEX		PEX compared to TEX		PEX compared to TEX	
TABLE 3	Protein Name	Symbol	Accession Number	pI	MW (kD)	UNIPROT MW (kd)	MS Protein Score	Proteoform Interpretation	P-Value	Diff. (%)	P-Value	Diff. (%)	P-Value	Diff. (%)
structural	Desmin	DES	P17661	5.32	50	54	509	INTACT	0.080	-48.410	0.137	44.153	0.006	95.133
structural	Desmin	DES	P17661	5.37	53	54	1170	INTACT	0.241	-26.728	0.525	-20.752	0.013	69.090
structural	Isoform 5 of Radixin	RDX	P35241-5	6.16	74	69	65	INTACT	0.225	11.022	0.581	-24.488	0.016	-33.774
transport	Hemoglobin subunit beta	HBB	P68871	6.27	14	16	321	INTACT	0.340	15.504	0.813	-7.165	0.022	-87.744
transport	Hemoglobin subunit beta	HBB	P68871	6.30	14	16	468	INTACT	0.092	51.528	0.744	5.507	0.023	-116.34
transport	Hemoglobin subunit delta	HBD	P02042	8.76	14	16	297	INTACT	0.156	28.821	0.721	-9.102	0.048	-63.425
glycolysis	Fructose-bisphosphate aldolase	ALDOA	H3BQN4	9.33	38	39	470	INTACT	0.078	-16.432	0.464	13.864	0.048	19.839
contractile	Myosin-7	MYH7	P12883	7.09	205	223	381	INTACT	0.384	6.656	0.687	5.378	0.051	50.011
Ca contractile	Tropomyosin beta chain	TPM2	P07951	5.20	35	33	120	INTACT	0.776	5.255	0.136	-26.258	0.052	-25.000
transport	Hemoglobin subunit alpha	HBA1	P69905	9.71	17	15	282	INTACT	0.684	-9.920	0.554	26.266	0.069	-133.86
structural	Ankyrin repeat domain-containing protein 2	ANKRD2	Q9GZV1	5.46	37	40	288	INTACT	0.062	31.041	0.686	4.410	0.077	-24.209
Ca contractile	Tropomyosin beta chain	TPM2	P07951	5.26	34	33	592	INTACT	0.778	3.525	0.543	10.321	0.094	-34.394
transport	Hemoglobin subunit alpha	HBA1	P69905	9.73	14	15	455	INTACT	0.555	4.742	0.646	-9.176	0.103	-27.367
metabolic	Heat shock protein beta-7	HSPB7	Q9UBY9	5.97	18	19	265	INTACT	0.231	12.992	0.481	-7.272	0.117	16.755
transport	Serum albumin	ALB	P02768	5.76	81	69	167	INTACT	0.027	-21.759	0.753	-5.935	0.135	-16.132
Ca	Bestrophin-3	BEST3	F8VVX2	8.68	19	17	46	INTACT	0.121	63.119	0.165	35.844	0.142	-36.226
degradation	Tripartite motif-containing protein 72	TRIM72	Q6ZMU5	6.15	49	53	343	INTACT	0.808	-5.893	0.311	18.660	0.150	18.779
structural	Actinin, alpha 2, isoform CRA_b	ACTN2	B2RCS5	4.91	100	104	62	INTACT	0.166	-29.194	0.686	-9.578	0.182	19.866
metabolic	Creatine kinase M-type	CKM	P06732	7.13	40	43	1010	INTACT	0.066	-27.345	0.908	2.031	0.184	18.477
metabolic	Heat shock protein beta-1	HSPB1	P04792	5.34	26	23	270	INTACT	0.402	20.749	0.255	-20.710	0.195	-39.983
Ca	Bestrophin-3	BEST3	F8VVX2	6.67	18	17	36	INTACT	0.906	-4.629	0.194	-31.746	0.197	22.953
contractile	Actin, alpha skeletal muscle	ACTA1	P68133	5.42	37	42	525	INTACT	0.727	-11.529	0.506	-8.823	0.213	21.408
transport	Carbonic anhydrase 3	CA3	P07451	5.27	28	30	38	INTACT	0.857	0.233	0.975	-1.180	0.221	-22.061
metabolic	Creatine kinase M-type	CKM	P06732	6.95	41	43	983	INTACT	0.088	-26.852	0.206	13.941	0.227	17.396
Ca	Bestrophin-3	BEST3	F8VVX2	4.51	15	17	36	INTACT	0.953	15.526	0.924	4.923	0.238	20.740
contractile	Myosin-2	MYH2	Q9UKX2	9.67	195	223	451	INTACT	0.858	6.756	0.167	27.252	0.245	24.083
Ca	Protein S100-A13	S100A13	Q99584	5.55	13	11	72	INTACT	0.476	17.335	0.364	43.234	0.256	-41.068
metabolic	Fructose-bisphosphate aldolase A	ALDOA	P04075	7.44	38	39	380	INTACT	0.987	0.173	0.739	2.399	0.304	19.704
contractile	Myosin light chain 1/3, skeletal muscle isoform	MYL1	P05976	5.24	21	21	673	INTACT	0.356	8.794	0.550	3.973	0.325	5.525
structural	Alpha-actinin-2	ACTN2	P35609	10.2	104	104	162	INTACT	0.645	18.989	0.214	-19.991	0.347	21.788
contractile	Myosin-2	MYH2	Q9UKX2	9.59	200	223	417	INTACT	0.607	-3.027	0.555	16.704	0.348	22.243
Ca contractile	Isoform 4 of Tropomyosin alpha-1 chain	TPM1	P09493-4	5.71	32	33	572	INTACT	0.892	4.122	0.909	-2.763	0.349	14.589
structural	Ankyrin repeat domain-containing protein 2	ANKRD2	Q9GZV1	5.57	37	40	325	INTACT	0.869	-5.352	0.924	-0.371	0.363	11.399
transcription	Elongation factor 1-alpha 1	EEF1A1	P68104	9.56	53	50	58	INTACT	0.621	15.888	0.525	12.458	0.404	-27.866
metabolic	Adenylate kinase isoenzyme 1	AK1	P00568	9.61	24	22	178	INTACT	0.458	12.384	0.411	28.689	0.419	-28.461
structural	Isoform 5 of Myosin-binding protein C, slow-type	MYBPC1	Q00872-5	5.94	131	128	733	INTACT	0.010	-48.654	0.752	10.405	0.432	10.425
Ca	Bestrophin-3	BEST3	F8VVX2	6.26	18	17	40	INTACT	0.113	34.200	0.222	-65.228	0.473	-14.341
contractile	Isoform MLC3 of Myosin light chain 1/3, skeletal muscle isoform	MYL1	P05976-2	5.17	20	21	199	INTACT	0.015	21.329	0.972	-0.861	0.484	-5.494
metabolic	Isoform 2 of Very long-chain specific acyl-CoA dehydrogenase, mitochondrial	ACADVL	P49748-2	8.74	68	70	96	INTACT	0.079	102.84	0.733	-25.798	0.511	1.538
Ca contractile	Troponin C type 2 (Fast), isoform CRA_a	TNNC2	C9J7T9	4.59	18	16	328	INTACT	0.779	-10.323	0.548	-6.770	0.558	11.516
contractile	Actin, alpha skeletal muscle	ACTA1	P68133	4.95	42	42	642	INTACT	0.258	19.077	0.870	-0.377	0.578	9.823
structural	Ankyrin repeat domain-containing protein 2	ANKRD2	Q9GZV1	5.50	37	40	143	INTACT	0.717	-6.417	0.383	-6.892	0.624	5.611
metabolic	Creatine kinase M-type	CKM	P06732	7.43	42	43	717	INTACT	0.309	-10.434	0.733	0.937	0.717	4.805
contractile	Isoform 2 of Actin, gamma-enteric smooth muscle	ACTG2	P63267-2	6.12	38	42	181	INTACT	0.329	-7.469	0.515	8.362	0.735	-2.324
Ca	Bestrophin-3	BEST3	F8VVX2	10.2	18	17	36	INTACT	0.357	-19.358	0.560	-10.515	0.743	-5.342
contractile	Myosin-1	MYH1	P12882	9.48	200	223	551	INTACT	0.681	-6.553	0.377	30.166	0.761	6.559
structural	Isoform 2 of Myosin-binding protein C, slow-type	MYBPC1	Q00872-2	5.70	141	128	625	INTACT	0.545	55.274	0.163	-38.197	0.774	-9.801
metabolic	Dihydrolipoyl dehydrogenase, mitochondrial	DLD	E9PEX6	6.82	54	52	271	INTACT	0.055	-25.170	0.253	13.173	0.782	7.113
contractile	Actin, alpha skeletal muscle	ACTA1	Q5T8M7	5.84	42	38	362	INTACT	0.302	-12.461	0.854	-0.356	0.787	2.569
contractile	Actin, alpha skeletal muscle	ACTA1	P68133	6.00	41	42	454	INTACT	0.237	-19.817	0.883	1.759	0.806	-2.055
transport	Myoglobin	MB	P02144	5.51	17	17	57	INTACT	0.175	24.261	0.970	-3.611	0.808	5.552
contractile	Myosin light chain 1/3, skeletal muscle isoform	MYL1	P05976	5.93	23	21	360	INTACT	0.756	-7.520	0.692	-9.072	0.816	7.240
contractile	Isoform MLC3 of Myosin light chain 1/3, skeletal muscle isoform	MYL1	P05976-2	4.92	22	21	98	INTACT	0.106	55.122	0.233	-49.330	0.829	-11.052
metabolic	Succinate dehydrogenase [ubiquinone] flavoprotein subunit, mitochondrial	SDHA	D6RFM5	6.22	68	64	155	INTACT	0.223	-28.107	0.548	14.639	0.918	4.386
contractile	Actin, alpha skeletal muscle	ACTA1	Q5T8M7	5.51	44	38	416	INTACT	0.125	-14.161	0.530	5.399	0.997	-0.892
unkknown	Putative BCoR-like protein 2	BCORP1	Q8N888	8.96	98	16	38	AGG	0.029	58.872	0.736	-3.683	0.016	-52.516
transcription	Ataxin-3	ATXN3	G3V3T0	8.62	19	11	37	AGG	0.017	80.334	0.020	51.427	0.026	-58.344
Ca contractile	TNNT1 protein	TNNT1	Q3B759	5.31	28	23	113	AGG	0.855	2.993	0.052	-26.801	0.034	-34.325
Ca	Bestrophin-3	BEST3	F8VVX2	4.68	31	17	42	AGG	0.805	4.236	0.018	-44.190	0.037	-57.999
contractile	Isoform 2 of Actin, gamma-enteric smooth muscle	ACTG2	P63267-2	4.92	141	42	78	AGG	0.210	-38.124	0.301	-47.025	0.046	51.284
transport	Hemoglobin subunit beta	HBB	P68871	6.19	28	16	157	AGG	0.372	13.750	0.685	-6.913	0.057	-73.020
Ca	Bestrophin-3	BEST3	F8VVX2	4.93	100	17	39	AGG	0.166	-44.406	0.213	-27.005	0.058	36.314
transport	Hemoglobin subunit alpha	HBA1	P69905	9.60	28	15	314	AGG	0.534	-11.151	0.432	14.437	0.068	-46.309
transport	Hemoglobin subunit alpha	HBA1	P69905	9.27	99	15	111	AGG	0.108	50.416	0.987	0.514	0.069	-78.634
metabolic	Isoform 2 of Glycogen phosphorylase, muscle form	PYGM	P11217-2	6.78	250	97	266	AGG	0.086	25.362	0.690	-2.100	0.079	35.555
Ca	Bestrophin-3	BEST3	F8VVX2	8.35	98	17	39	AGG	0.029	78.392	0.511	12.470	0.090	-49.545
Ca contractile	Troponin T, fast skeletal muscle	TNNT3	H9KVA2	6.21	35	28	234	AGG	0.006	-30.747	0.447	-17.370	0.120	18.771
contractile	Actin, alpha skeletal muscle	ACTA1	P68133	5.32	97	42	632	AGG	0.104	-52.053	0.742	-18.619	0.129	-32.058
transport	Hemoglobin subunit alpha	HBA1	P69905	9.71	18	15	262	AGG	0.907	6.309	0.770	41.961	0.157	-215.59
degradation	E3 ubiquitin-protein ligase listerin	LTN1	H7BYG8	5.14	126	91	44	AGG	0.705	-2.566	0.984	-1.767	0.218	-20.295
Ca contractile	Isoform 4 of Tropomyosin alpha-1 chain	TPM1	P09493-4	4.98	127	33	562	AGG	0.111	39.660	0.704	1.264	0.237	28.225
contractile	Actin, alpha skeletal muscle	ACTA1	P68133	5.32	123	42	626	AGG	0.098	-42.120	0.873	-16.347	0.241	-20.562
transcription	Ataxin-3	ATXN3	G3V3T0	7.14	18	11	41	AGG	0.065	54.508	0.701	7.562	0.256	13.650
contractile	Isoform 2 of Actin, gamma-enteric smooth muscle	ACTG2	P63267-2	4.69	195	42	93	AGG	0.811	-0.350	0.653	-23.508	0.257	-32.646
Ca contractile	Tropomyosin beta chain	TPM2	P07951	5.01	159	33	769	AGG	0.112	33.282	0.243	-28.410	0.282	14.917
contractile	Isoform 2 of Actin, gamma-enteric smooth muscle	ACTG2	P63267-2	4.75	65	42	112	AGG	0.289	-25.580	0.088	-59.102	0.316	31.025
transport	Hemoglobin subunit beta	HBB	P68871	5.86	28	16	169	AGG	0.810	6.106	0.154	-44.961	0.329	-245.69
Ca	Bestrophin-3	BEST3	F8VVX2	6.35	173	17	53	AGG	0.040	-20.977	0.146	-30.519	0.438	-15.299
Ca	Bestrophin-3	BEST3	F8VVX2	4.73	20	17	34	AGG	0.731	-3.489	0.254	-14.732	0.491	-7.829
contractile	Isoform 2 of Actin, gamma-enteric smooth muscle	ACTG2	P63267-2	4.78	100	42	65	AGG	0.025	-39.528	0.190	-32.966	0.498	5.611
transport	Carbonic anhydrase 3	CA3	P07451	7.16	111	30	204	AGG	0.423	30.272	0.373	29.812	0.688	-7.064
metabolic	Mitochondrial inner membrane protein	IMMT	C9J406	5.75	87	73	148	AGG	0.392	-9.729	0.084	23.126	0.715	3.168
contractile	Myosin light chain 1/3, skeletal muscle isoform	MYL1	P05976	5.94	38	21	415	AGG	0.372	-14.587	0.223	-19.102	0.793	10.226
metabolic	Cytochrome b-c1 complex subunit Rieske, mitochondrial	UQCRFS1	P47985	6.40	53	30	114	AGG	0.885	-1.203	0.962	-0.515	0.807	1.743
metabolic	Acyl-coenzyme A synthetase ACSM2B, mitochondrial (Fragment)	ACSM2B	H3BQ84	4.93	29	12	42	FRAG	0.344	16.126	0.017	-66.970	0.007	-72.367
metabolic	Acyl-coenzyme A synthetase ACSM2B, mitochondrial (Fragment)	ACSM2B	H3BQ84	5.31	18	12	47	FRAG	0.960	-1.433	0.666	-13.467	0.008	-52.183
metabolic	Pyruvate kinase (Fragment)	PKM	H3BTN5	8.16	57	53	528	FRAG	0.782	0.569	0.982	0.676	0.013	66.495
transport	Hemoglobin subunit alpha	HBA1	P69905	5.24	13	15	152	FRAG	0.716	1.770	0.581	-5.989	0.034	-37.938
structural	Keratin, type I cytoskeletal 10	KRT10	P13645	5.63	21	59	200	FRAG	0.962	-6.831	0.884	-4.235	0.057	-33.182
unknown	Microtubule-actin cross-linking factor 1, isoforms 1/2/3/5 (Fragment)	MACF1	H0Y390	9.41	137	506	40	FRAG	0.375	8.112	0.230	24.899	0.062	-39.800
transport	Myosin-7	MYH7	P12883	9.27	99	223	119	FRAG	0.108	50.416	0.987	0.514	0.069	-78.634
metabolic	Short-chain specific acyl-CoA dehydrogenase, mitochondrial	ACADS	P16219	6.30	38	44	95	FRAG	0.029	-49.840	0.113	27.213	0.072	26.078
structural	Unconventional myosin-XIX (Fragment)	MYO19	K7EMZ0	4.83	13	8	33	FRAG	0.389	18.656	0.313	-38.380	0.077	-40.357
transport	Hemoglobin subunit beta	HBB	P68871	4.16	13	16	261	FRAG	0.589	18.059	0.101	-95.360	0.081	-71.546
structural	Nebulin	NEB	F8WCL5	8.96	109	773	67	FRAG	0.011	50.238	0.696	23.149	0.131	-27.535
transport	Hemoglobin subunit beta	HBB	P68871	6.28	13	16	374	FRAG	0.185	27.182	0.480	-13.390	0.134	-56.692
transport	Fatty acid-binding protein, heart (Fragment)	FABP3	S4R371	5.99	14	15	574	FRAG	0.051	19.332	0.817	3.139	0.155	-24.675
contractile	Myosin-2	MYH2	Q9UKX2	5.60	143	223	384	FRAG	0.281	-19.685	0.820	-7.798	0.159	-22.691
structural	Nebulin	NEB	F8WCL5	9.35	113	773	112	FRAG	0.620	5.200	0.687	19.867	0.177	-33.573
contractile	Myosin regulatory light chain 2, skeletal muscle isoform (Fragment)	MYLPF	H3BML9	6.10	16	13	64	FRAG	0.282	-17.765	0.258	19.772	0.193	31.037
contractile	Actin, alpha cardiac muscle 1	ACTC1	P68032	5.14	31	42	402	FRAG	0.055	76.671	0.877	0.631	0.249	-42.101
structural	Cofilin-1 (Fragment)	CFL1	E9PLJ3	6.04	17	9	66	FRAG	0.130	19.294	0.247	-21.046	0.260	20.168
transport	Hemoglobin subunit beta	HBB	P68871	4.13	13	16	423	FRAG	0.695	23.772	0.130	######	0.267	-66.555
transport	Myoglobin (Fragment)	MB	F2Z337	4.89	17	9	112	FRAG	0.520	23.269	0.678	-10.150	0.291	-17.412
unknown	Serine/threonine-protein phosphatase 4 regulatory subunit 4	PPP4R4	Q6NUP7	7.00	18	99	32	FRAG	0.257	48.855	0.358	31.896	0.292	34.710
metabolic	Calsequestrin-1	CASQ1	P31415	4.75	34	45	117	FRAG	0.003	46.920	0.892	7.351	0.298	-19.602
contractile	Myosin regulatory light chain 2, skeletal muscle isoform (Fragment)	MYLPF	H3BML9	5.17	16	13	621	FRAG	0.361	11.836	0.877	-5.452	0.309	-11.894
contractile	Myosin regulatory light chain 2, skeletal muscle isoform (Fragment)	MYLPF	H3BN54	5.17	37	15	662	FRAG	0.441	16.372	0.668	8.082	0.326	-17.139
transcription	Histidine protein methyltransferase 1 homolog	METTL18	O95568	4.89	20	42	38	FRAG	0.261	-29.554	0.120	-32.688	0.381	16.099
structural	Keratin, type I cytoskeletal 10	KRT10	P13645	5.22	14	59	250	FRAG	0.007	22.120	0.219	-18.091	0.449	-6.599
structural	Keratin, type II cytoskeletal 1	KRT1	P04264	4.87	31	66	41	FRAG	0.026	59.615	0.240	-24.818	0.464	-20.453
contractile	Myosin regulatory light chain 2, skeletal muscle isoform (Fragment)	MYLPF	H3BPK4	6.22	17	22	90	FRAG	0.100	27.375	0.748	-9.993	0.471	-11.629
structural	Keratin, type II cytoskeletal 6A	KRT6A	P02538	6.13	33	60	435	FRAG	0.530	12.109	0.448	-9.065	0.517	12.195
glycolysis	Enolase (Fragment)	ENO3	E5RGZ4	7.90	111	30	104	FRAG	0.833	3.854	0.755	1.807	0.584	10.639
contractile	Myosin regulatory light chain 2, skeletal muscle isoform (Fragment)	MYLPF	H3BML9	6.86	18	13	54	FRAG	0.135	25.455	0.450	-13.981	0.650	9.213
structural	Keratin, type II cytoskeletal 2 epidermal	KRT2	P35908	5.90	29	65	235	FRAG	0.328	30.399	0.085	-73.101	0.650	37.656
contractile	Isoform 2 of Actin, gamma-enteric smooth muscle	ACTG2	P63267-2	5.38	36	42	171	FRAG	0.528	-6.446	0.866	3.103	0.653	10.546
transport	Myoglobin (Fragment)	MB	F2Z337	4.22	31	9	74	FRAG	0.656	-6.354	0.018	-80.282	0.656	-10.552
contractile	Myosin regulatory light chain 2, skeletal muscle isoform (Fragment)	MYLPF	H3BML9	6.33	18	13	129	FRAG	0.024	31.687	0.447	-25.945	0.723	-4.776
contractile	Myosin-1	MYH1	P12882	9.92	191	223	358	FRAG	0.515	-13.379	0.152	18.255	0.729	7.087
transcription	Keratin, type I cytoskeletal 10	KRT10	P13645	6.77	18	59	69	FRAG	0.801	-6.667	0.832	-3.348	0.786	16.080
transport	Myoglobin (Fragment)	MB	F2Z337	4.14	16	9	107	FRAG	0.675	-10.865	0.327	-24.146	0.790	0.706
contractile	Actin, alpha cardiac muscle 1	ACTC1	P68032	5.35	34	42	334	FRAG	0.697	2.570	0.397	-14.601	0.808	0.945
transport	Myoglobin (Fragment)	MB	F2Z337	4.75	16	9	115	FRAG	0.622	25.746	0.552	-14.003	0.817	-15.234
structural	Keratin, type I cytoskeletal 9	KRT9	P35527	7.05	18	62	173	FRAG	0.098	35.935	0.430	11.375	0.873	3.053
contractile	Myosin regulatory light chain 2, skeletal muscle isoform (Fragment)	MYLPF	H3BN54	5.20	16	15	797	FRAG	0.935	-4.170	0.635	6.043	0.881	7.035
contractile	Myosin regulatory light chain 2, skeletal muscle isoform (Fragment)	MYLPF	H3BML9	6.36	18	13	280	FRAG	0.263	14.359	0.801	-7.922	0.938	-3.447
transport	Myoglobin (Fragment)	MB	B0QYF8	9.37	16	16	164	FRAG	0.509	16.853	0.325	16.052	0.946	3.617
structural	Desmin	DES	P17661	5.13	37	54	254	FRAG	0.707	-5.214	0.847	3.286	0.958	1.844
metabolic	Isoform 2 of Glycogen phosphorylase, muscle form	PYGM	P11217-2	5.98	28	97	155	FRAG	0.374	21.548	0.026	58.431	0.967	0.421
unknown	#N/A	#N/A	#N/A	10.2	19	#N/A	#N/A	#N/A	0.266	-17.562	0.331	-38.427	0.414	8.669
								**Total**		**pre**		**mid**		**post**
							**intact**	55		3		0		7
							**aggregate**	29		6		2		5
							**fragment**	46		6		3		4
							**TOTAL**	130		15		5		16

**Table 4:** Protein phosphorylation differences in PEX vs TEX. Differences between PEX and TEX in all identified spots. Ordering within the table is based on proteoform interpretation (i.e. intact, aggregate, fragment) and p-values (2-tailed, unpaired t-tests) of the post comparison between PEX and TEX. P-values < 0.05 are shaded in yellow. Differences (%) within each comparison are shaded to indicate higher (red) or lower (blue) values in PEX relative to TEX.

									pre		mid		post	
									PEX compared to TEX		PEX compared to TEX		PEX compared to TEX	
TABLE 4	Protein Name	Symbol	Accession Number	pI	MW (kD)	UNIPROT MW (kd)	MS Protein Score	Proteoform Interpretation	P-Value	Diff. (%)	P-Value	Diff. (%)	P-Value	Diff. (%)
metabolic	Creatine kinase M-type	CKM	P06732	7.13	40	43	1010	INTACT	0.236	-20.578	0.256	13.685	0.001	28.608
metabolic	Creatine kinase M-type	CKM	P06732	6.95	41	43	983	INTACT	0.390	-7.588	0.273	10.779	0.001	32.480
contractile	Myosin-2	MYH2	Q9UKX2	9.59	200	223	417	INTACT	0.780	-2.484	0.171	48.475	0.006	66.349
contractile	Myosin-1	MYH1	P12882	9.48	200	223	551	INTACT	0.794	-1.674	0.138	41.428	0.006	62.886
contractile	Actin, alpha skeletal muscle	ACTA1	P68133	6.00	41	42	454	INTACT	0.714	4.888	0.275	13.461	0.008	32.904
metabolic	Creatine kinase M-type	CKM	P06732	7.43	42	43	717	INTACT	0.439	7.649	0.855	-4.380	0.012	40.507
Ca contractile	Troponin C type 2 (Fast), isoform CRA_a	TNNC2	C9J7T9	4.59	18	16	328	INTACT	0.085	-26.115	0.702	5.751	0.021	41.717
metabolic	Fructose-bisphosphate aldolase A	ALDOA	P04075	7.44	38	39	380	INTACT	0.706	3.300	0.805	4.543	0.025	40.745
contractile	Myosin-2	MYH2	Q9UKX2	9.67	195	223	451	INTACT	0.775	-3.146	0.039	71.296	0.026	41.678
contractile	Actin, alpha skeletal muscle	ACTA1	Q5T8M7	5.51	44	38	416	INTACT	0.310	16.739	0.203	23.749	0.038	41.029
Ca	Bestrophin-3	BEST3	F8VVX2	8.68	19	17	46	INTACT	0.898	-22.045	0.695	5.506	0.076	-25.687
Ca contractile	Isoform 4 of Tropomyosin alpha-1 chain	TPM1	P09493-4	5.71	32	33	572	INTACT	0.903	-13.820	0.912	3.034	0.080	-42.144
contractile	Actin, alpha skeletal muscle	ACTA1	Q5T8M7	5.84	42	38	362	INTACT	0.835	1.528	0.173	17.333	0.087	18.782
structural	Ankyrin repeat domain-containing protein 2	ANKRD2	Q9GZV1	5.57	37	40	325	INTACT	0.955	1.949	0.479	9.030	0.107	24.714
structural	Ankyrin repeat domain-containing protein 2	ANKRD2	Q9GZV1	5.50	37	40	143	INTACT	0.986	3.174	0.767	1.585	0.115	24.493
structural	Ankyrin repeat domain-containing protein 2	ANKRD2	Q9GZV1	5.46	37	40	288	INTACT	0.925	0.014	0.389	9.426	0.115	23.931
metabolic	Dihydrolipoyl dehydrogenase, mitochondrial	DLD	E9PEX6	6.82	54	52	271	INTACT	0.123	36.434	0.332	-25.548	0.119	-40.722
Ca contractile	Tropomyosin beta chain	TPM2	P07951	5.26	34	33	592	INTACT	0.333	-14.320	0.900	-1.025	0.120	-41.172
structural	Alpha-actinin-2	ACTN2	P35609	10.2	104	104	162	INTACT	0.389	14.501	0.721	8.119	0.130	-45.507
contractile	Myosin light chain 1/3, skeletal muscle isoform	MYL1	P05976	5.93	23	21	360	INTACT	0.110	61.540	0.822	0.830	0.141	13.119
structural	Desmin	DES	P17661	5.37	53	54	1170	INTACT	0.567	8.370	0.185	17.928	0.143	24.408
transport	Hemoglobin subunit beta	HBB	P68871	6.27	14	16	321	INTACT	0.344	-12.978	0.519	-14.885	0.148	-49.523
metabolic	Heat shock protein beta-1	HSPB1	P04792	5.34	26	23	270	INTACT	0.626	8.640	0.062	-21.255	0.158	-23.606
contractile	Actin, alpha skeletal muscle	ACTA1	P68133	5.42	37	42	525	INTACT	0.748	-3.572	0.723	-8.574	0.160	24.234
transport	Hemoglobin subunit beta	HBB	P68871	6.30	14	16	468	INTACT	0.684	-3.805	0.616	3.738	0.199	-72.209
transport	Myoglobin	MB	P02144	5.51	17	17	57	INTACT	0.195	-26.622	0.515	-17.249	0.210	-17.022
contractile	Isoform MLC3 of Myosin light chain 1/3, skeletal muscle isoform	MYL1	P05976-2	4.92	22	21	98	INTACT	0.944	1.879	0.896	1.562	0.239	16.453
metabolic	Adenylate kinase isoenzyme 1	AK1	P00568	9.61	24	22	178	INTACT	0.748	12.248	0.716	1.923	0.299	-13.293
transcription	Elongation factor 1-alpha 1	EEF1A1	P68104	9.56	53	50	58	INTACT	0.394	13.572	0.548	-21.987	0.304	-33.996
contractile	Isoform MLC3 of Myosin light chain 1/3, skeletal muscle isoform	MYL1	P05976-2	5.17	20	21	199	INTACT	0.082	-27.611	0.128	-23.383	0.338	-14.496
Ca	Bestrophin-3	BEST3	F8VVX2	6.26	18	17	40	INTACT	0.233	-23.950	0.142	-33.146	0.359	-12.714
transport	Hemoglobin subunit alpha	HBA1	P69905	9.73	14	15	455	INTACT	0.752	1.851	0.764	-5.764	0.386	-12.319
structural	Actinin, alpha 2, isoform CRA_b	ACTN2	B2RCS5	4.91	100	104	62	INTACT	0.114	32.876	0.141	27.388	0.390	-16.466
glycolysis	Fructose-bisphosphate aldolase	ALDOA	H3BQN4	9.33	38	39	470	INTACT	0.053	41.254	0.869	4.377	0.396	-20.963
transport	Hemoglobin subunit delta	HBD	P02042	8.76	14	16	297	INTACT	0.115	27.180	0.701	5.088	0.404	-28.722
contractile	Myosin light chain 1/3, skeletal muscle isoform	MYL1	P05976	5.24	21	21	673	INTACT	0.005	-33.802	0.870	-1.636	0.409	-8.236
Ca	Protein S100-A13	S100A13	Q99584	5.55	13	11	72	INTACT	0.336	24.532	0.663	14.737	0.422	-11.854
transport	Serum albumin	ALB	P02768	5.76	81	69	167	INTACT	0.634	-5.665	0.520	6.823	0.483	6.108
Ca	Bestrophin-3	BEST3	F8VVX2	10.2	18	17	36	INTACT	0.806	13.608	0.796	-13.325	0.486	-17.497
degradation	Tripartite motif-containing protein 72	TRIM72	Q6ZMU5	6.15	49	53	343	INTACT	0.814	2.039	0.115	26.199	0.493	42.350
transport	Carbonic anhydrase 3	CA3	P07451	5.27	28	30	38	INTACT	0.315	8.136	0.763	4.157	0.494	-9.057
contractile	Isoform 2 of Actin, gamma-enteric smooth muscle	ACTG2	P63267-2	6.12	38	42	181	INTACT	0.972	-0.010	0.208	12.809	0.499	12.872
metabolic	Heat shock protein beta-7	HSPB7	Q9UBY9	5.97	18	19	265	INTACT	0.378	-16.100	0.930	-8.510	0.520	11.246
Ca contractile	Tropomyosin beta chain	TPM2	P07951	5.20	35	33	120	INTACT	0.745	-10.235	0.152	-33.648	0.558	-4.471
metabolic	Isoform 2 of Very long-chain specific acyl-CoA dehydrogenase, mitochondrial	ACADVL	P49748-2	8.74	68	70	96	INTACT	0.534	-8.172	0.728	-7.823	0.582	14.958
metabolic	Succinate dehydrogenase [ubiquinone] flavoprotein subunit, mitochondrial	SDHA	D6RFM5	6.22	68	64	155	INTACT	0.989	-4.287	0.690	8.013	0.587	-11.022
structural	Desmin	DES	P17661	5.32	50	54	509	INTACT	0.791	5.346	0.922	-1.059	0.676	6.647
Ca	Bestrophin-3	BEST3	F8VVX2	6.67	18	17	36	INTACT	0.579	-1.988	0.278	-26.803	0.677	-2.112
transport	Hemoglobin subunit alpha	HBA1	P69905	9.71	17	15	282	INTACT	0.225	20.503	0.896	-0.789	0.707	4.098
structural	Isoform 5 of Myosin-binding protein C, slow-type	MYBPC1	Q00872-5	5.94	131	128	733	INTACT	0.614	-9.207	0.690	-6.968	0.729	-9.249
structural	Isoform 5 of Radixin	RDX	P35241-5	6.16	74	69	65	INTACT	0.641	-4.392	0.976	2.510	0.806	-12.052
structural	Isoform 2 of Myosin-binding protein C, slow-type	MYBPC1	Q00872-2	5.70	141	128	625	INTACT	0.655	2.407	0.982	5.312	0.823	-0.430
contractile	Actin, alpha skeletal muscle	ACTA1	P68133	4.95	42	42	642	INTACT	0.131	18.485	0.451	-7.035	0.959	-0.796
contractile	Myosin-7	MYH7	P12883	7.09	205	223	381	INTACT	0.122	-31.226	0.902	3.242	0.976	-3.985
Ca	Bestrophin-3	BEST3	F8VVX2	4.51	15	17	36	INTACT	0.481	16.275	0.884	3.724	0.986	-1.701
transport	Carbonic anhydrase 3	CA3	P07451	7.16	111	30	204	AGG	0.998	3.046	0.125	-29.160	0.005	-72.200
transport	Hemoglobin subunit alpha	HBA1	P69905	9.27	99	15	111	AGG	0.836	9.037	0.575	-11.737	0.018	-69.453
Ca	Bestrophin-3	BEST3	F8VVX2	6.35	173	17	53	AGG	0.560	-5.339	0.540	-21.994	0.021	-52.825
transport	Hemoglobin subunit beta	HBB	P68871	6.19	28	16	157	AGG	0.867	0.853	0.321	26.987	0.073	-34.374
Ca contractile	TNNT1 protein	TNNT1	Q3B759	5.31	28	23	113	AGG	0.134	-29.878	0.058	-36.719	0.117	-19.609
transcription	Ataxin-3	ATXN3	G3V3T0	7.14	18	11	41	AGG	0.500	-10.578	0.524	-12.336	0.169	20.236
unkknown	Putative BCoR-like protein 2	BCORP1	Q8N888	8.96	98	16	38	AGG	0.504	9.082	0.456	-19.685	0.247	-20.274
metabolic	Mitochondrial inner membrane protein	IMMT	C9J406	5.75	87	73	148	AGG	0.199	-20.645	0.879	-7.151	0.267	12.084
contractile	Isoform 2 of Actin, gamma-enteric smooth muscle	ACTG2	P63267-2	4.78	100	42	65	AGG	0.063	37.899	0.099	-46.297	0.268	14.692
contractile	Actin, alpha skeletal muscle	ACTA1	P68133	5.32	123	42	626	AGG	0.575	-13.189	0.802	-4.538	0.291	-22.864
Ca contractile	Troponin T, fast skeletal muscle	TNNT3	H9KVA2	6.21	35	28	234	AGG	0.578	7.548	0.559	-11.346	0.310	15.966
contractile	Isoform 2 of Actin, gamma-enteric smooth muscle	ACTG2	P63267-2	4.92	141	42	78	AGG	0.574	11.190	0.843	2.769	0.394	15.285
contractile	Isoform 2 of Actin, gamma-enteric smooth muscle	ACTG2	P63267-2	4.69	195	42	93	AGG	0.217	22.229	0.645	34.760	0.411	-13.675
transport	Hemoglobin subunit alpha	HBA1	P69905	9.71	18	15	262	AGG	0.937	-0.605	0.840	2.289	0.472	10.152
contractile	Actin, alpha skeletal muscle	ACTA1	P68133	5.32	97	42	632	AGG	0.579	7.133	0.110	-27.469	0.527	-12.206
Ca	Bestrophin-3	BEST3	F8VVX2	8.35	98	17	39	AGG	0.630	13.637	0.570	14.054	0.564	-28.371
Ca	Bestrophin-3	BEST3	F8VVX2	4.68	31	17	42	AGG	0.246	21.596	0.644	-6.208	0.573	-5.674
Ca contractile	Tropomyosin beta chain	TPM2	P07951	5.01	159	33	769	AGG	0.969	3.989	0.840	2.313	0.652	-13.105
degradation	E3 ubiquitin-protein ligase listerin	LTN1	H7BYG8	5.14	126	91	44	AGG	0.046	13.411	0.934	-0.232	0.654	-6.187
metabolic	Cytochrome b-c1 complex subunit Rieske, mitochondrial	UQCRFS1	P47985	6.40	53	30	114	AGG	0.458	8.470	0.886	0.245	0.661	-3.256
metabolic	Isoform 2 of Glycogen phosphorylase, muscle form	PYGM	P11217-2	6.78	250	97	266	AGG	0.082	-31.134	0.572	10.577	0.665	-23.037
contractile	Isoform 2 of Actin, gamma-enteric smooth muscle	ACTG2	P63267-2	4.75	65	42	112	AGG	0.226	31.042	0.164	-52.378	0.708	3.643
transcription	Ataxin-3	ATXN3	G3V3T0	8.62	19	11	37	AGG	0.842	-0.571	0.332	20.915	0.722	17.997
Ca	Bestrophin-3	BEST3	F8VVX2	4.73	20	17	34	AGG	0.829	2.310	0.613	-5.029	0.820	-4.577
contractile	Myosin light chain 1/3, skeletal muscle isoform	MYL1	P05976	5.94	38	21	415	AGG	0.744	-3.570	0.170	32.708	0.882	-2.190
Ca contractile	Isoform 4 of Tropomyosin alpha-1 chain	TPM1	P09493-4	4.98	127	33	562	AGG	0.074	-39.608	0.892	1.636	0.892	10.182
Ca	Bestrophin-3	BEST3	F8VVX2	4.93	100	17	39	AGG	0.521	4.711	0.026	57.228	0.915	6.274
transport	Hemoglobin subunit beta	HBB	P68871	5.86	28	16	169	AGG	0.317	-11.886	0.549	15.431	0.928	-6.836
transport	Hemoglobin subunit alpha	HBA1	P69905	9.60	28	15	314	AGG	0.995	1.731	0.300	-24.725	0.968	2.617
transport	Hemoglobin subunit beta	HBB	P68871	6.28	13	16	374	FRAG	0.417	-10.748	0.702	-6.219	0.016	-86.174
transport	Myosin-7	MYH7	P12883	9.27	99	223	119	FRAG	0.836	9.037	0.575	-11.737	0.018	-69.453
unknown	Microtubule-actin cross-linking factor 1, isoforms 1/2/3/5 (Fragment)	MACF1	H0Y390	9.41	137	506	40	FRAG	0.044	-28.026	0.903	-0.069	0.031	-42.117
metabolic	Short-chain specific acyl-CoA dehydrogenase, mitochondrial	ACADS	P16219	6.30	38	44	95	FRAG	0.531	9.903	0.205	13.690	0.041	22.255
metabolic	Calsequestrin-1	CASQ1	P31415	4.75	34	45	117	FRAG	0.200	-12.681	0.657	-3.224	0.050	-32.049
structural	Nebulin	NEB	F8WCL5	8.96	109	773	67	FRAG	0.458	-11.145	0.665	2.703	0.054	-25.416
structural	Nebulin	NEB	F8WCL5	9.35	113	773	112	FRAG	0.560	-12.816	0.637	-3.076	0.067	-62.524
structural	Keratin, type I cytoskeletal 10	KRT10	P13645	5.63	21	59	200	FRAG	0.343	13.479	0.503	-8.069	0.093	-29.026
metabolic	Pyruvate kinase (Fragment)	PKM	H3BTN5	8.16	57	53	528	FRAG	0.798	-2.231	0.478	-8.463	0.094	-22.549
glycolysis	Enolase (Fragment)	ENO3	E5RGZ4	7.90	111	30	104	FRAG	0.332	24.513	0.007	60.388	0.097	-36.006
transport	Fatty acid-binding protein, heart (Fragment)	FABP3	S4R371	5.99	14	15	574	FRAG	0.678	-6.509	0.759	3.900	0.111	-45.740
transport	Myoglobin (Fragment)	MB	F2Z337	4.14	16	9	107	FRAG	0.971	7.119	0.184	-38.576	0.130	42.751
structural	Unconventional myosin-XIX (Fragment)	MYO19	K7EMZ0	4.83	13	8	33	FRAG	0.549	20.606	0.832	-3.361	0.131	-22.350
contractile	Myosin-1	MYH1	P12882	9.92	191	223	358	FRAG	0.666	-7.265	0.030	33.180	0.179	25.737
structural	Keratin, type I cytoskeletal 10	KRT10	P13645	5.22	14	59	250	FRAG	0.556	-5.304	0.676	7.043	0.208	18.660
metabolic	Isoform 2 of Glycogen phosphorylase, muscle form	PYGM	P11217-2	5.98	28	97	155	FRAG	0.118	-43.708	0.518	-9.793	0.256	-34.741
contractile	Actin, alpha cardiac muscle 1	ACTC1	P68032	5.35	34	42	334	FRAG	0.960	-2.923	0.554	10.697	0.272	15.956
structural	Keratin, type I cytoskeletal 9	KRT9	P35527	7.05	18	62	173	FRAG	0.093	-43.326	0.516	-13.255	0.354	12.637
contractile	Myosin regulatory light chain 2, skeletal muscle isoform (Fragment)	MYLPF	H3BML9	6.10	16	13	64	FRAG	0.496	-2.281	0.627	-22.567	0.380	13.938
contractile	Myosin regulatory light chain 2, skeletal muscle isoform (Fragment)	MYLPF	H3BN54	5.17	37	15	662	FRAG	0.253	-22.430	0.340	-45.149	0.406	-29.019
metabolic	Acyl-coenzyme A synthetase ACSM2B, mitochondrial (Fragment)	ACSM2B	H3BQ84	5.31	18	12	47	FRAG	0.049	-34.420	0.218	-17.235	0.435	-9.440
contractile	Actin, alpha cardiac muscle 1	ACTC1	P68032	5.14	31	42	402	FRAG	0.940	-1.558	0.735	3.549	0.458	-13.188
transport	Hemoglobin subunit beta	HBB	P68871	4.16	13	16	261	FRAG	0.255	26.733	0.151	-48.021	0.522	-16.155
contractile	Myosin regulatory light chain 2, skeletal muscle isoform (Fragment)	MYLPF	H3BN54	5.20	16	15	797	FRAG	0.536	-9.301	0.702	-4.549	0.531	8.694
metabolic	Acyl-coenzyme A synthetase ACSM2B, mitochondrial (Fragment)	ACSM2B	H3BQ84	4.93	29	12	42	FRAG	0.323	15.002	0.502	18.870	0.575	6.125
transport	Myoglobin (Fragment)	MB	F2Z337	4.75	16	9	115	FRAG	0.940	7.459	0.992	9.985	0.628	17.635
contractile	Myosin regulatory light chain 2, skeletal muscle isoform (Fragment)	MYLPF	H3BML9	5.17	16	13	621	FRAG	0.277	-25.666	0.684	4.901	0.685	5.549
structural	Cofilin-1 (Fragment)	CFL1	E9PLJ3	6.04	17	9	66	FRAG	0.262	-20.414	0.414	-24.698	0.704	3.539
contractile	Myosin regulatory light chain 2, skeletal muscle isoform (Fragment)	MYLPF	H3BML9	6.36	18	13	280	FRAG	0.379	-12.307	0.458	-23.247	0.710	-0.061
transcription	Histidine protein methyltransferase 1 homolog	METTL18	O95568	4.89	20	42	38	FRAG	0.268	22.890	0.807	-0.273	0.728	-1.962
transport	Hemoglobin subunit beta	HBB	P68871	4.13	13	16	423	FRAG	0.128	34.368	0.004	-75.666	0.778	-6.020
transport	Hemoglobin subunit alpha	HBA1	P69905	5.24	13	15	152	FRAG	0.104	15.375	0.173	13.997	0.780	3.232
transport	Myoglobin (Fragment)	MB	F2Z337	4.22	31	9	74	FRAG	0.368	16.711	0.020	-92.540	0.784	-8.679
structural	Keratin, type II cytoskeletal 1	KRT1	P04264	4.87	31	66	41	FRAG	0.910	4.862	0.672	13.940	0.799	3.891
transport	Myoglobin (Fragment)	MB	F2Z337	4.89	17	9	112	FRAG	0.731	9.086	0.902	1.484	0.833	-4.530
contractile	Isoform 2 of Actin, gamma-enteric smooth muscle	ACTG2	P63267-2	5.38	36	42	171	FRAG	0.466	-14.468	0.841	11.175	0.837	10.738
contractile	Myosin regulatory light chain 2, skeletal muscle isoform (Fragment)	MYLPF	H3BML9	6.86	18	13	54	FRAG	0.445	-8.197	0.986	-2.011	0.850	-8.166
structural	Keratin, type II cytoskeletal 6A	KRT6A	P02538	6.13	33	60	435	FRAG	0.815	-4.139	0.730	-2.768	0.852	0.892
transcription	Keratin, type I cytoskeletal 10	KRT10	P13645	6.77	18	59	69	FRAG	0.519	-2.711	0.405	-32.530	0.857	-8.675
structural	Desmin	DES	P17661	5.13	37	54	254	FRAG	0.195	-32.632	0.463	-25.495	0.870	1.254
structural	Keratin, type II cytoskeletal 2 epidermal	KRT2	P35908	5.90	29	65	235	FRAG	0.077	31.922	0.637	12.027	0.888	-6.467
contractile	Myosin regulatory light chain 2, skeletal muscle isoform (Fragment)	MYLPF	H3BML9	6.33	18	13	129	FRAG	0.297	-20.016	0.390	-23.974	0.918	-10.137
contractile	Myosin-2	MYH2	Q9UKX2	5.60	143	223	384	FRAG	0.195	24.861	0.555	10.880	0.923	-1.428
contractile	Myosin regulatory light chain 2, skeletal muscle isoform (Fragment)	MYLPF	H3BPK4	6.22	17	22	90	FRAG	0.510	-15.747	0.346	-26.993	0.941	-8.377
transport	Myoglobin (Fragment)	MB	B0QYF8	9.37	16	16	164	FRAG	0.007	46.073	0.318	-18.533	0.965	-2.515
unknown	Serine/threonine-protein phosphatase 4 regulatory subunit 4	PPP4R4	Q6NUP7	7.00	18	99	32	FRAG	0.178	-29.181	0.958	-0.391	0.982	0.879
unknown	#N/A	#N/A	#N/A	10.2	19	#N/A	#N/A	#N/A	0.922	-11.276	0.180	-30.059	0.930	2.672
								**Total**		**pre**		**mid**		**post**
							**intact**	55		1		1		10
							**aggregate**	29		1		1		3
							**fragment**	46		3		4		4
							**TOTAL**	130		5		6		17

The t-tests described above and shown in **Tables 1–4** were conducted according to standard gel spot picking procedures and compared data sets spot by spot (univariate analysis). In addition, we performed multivariate data analysis to reveal patterns, looking at our data more globally. For this purpose, we used Principal Components Analysis (PCA), a “dimension reduction” algorithm. PCA was performed using the analysis program TIBCO Spotfire. PCA transforms the original set of variables (spot intensities for selected samples) to a new, orthogonal, set of variables (the principal components) such that most of the variability in the original data is captured in the top few of the new components. In general, it is sufficient to examine the first 2 or 3 components to discover any existing clustering in the data. Our approach was to plot PCA1 vs. PCA2 vs. PCA3. We performed this calculation for the total abundance and phosphorylation data separately.

## Results

From a total of 932 protein spots detected, 130 spots emerged as potentially altered in terms of phosphoprotein or total protein abundance due to HDBR (pre, mid, post) and/or countermeasures (CON, PEX, TEX), and were subjected to MS analysis for protein identification. Out of 130 spots, 129 were identified by MS while 1 remained unidentified. Among the identified spots, 55 proteoforms had molecular weights (MW) that were within 15% of predicted MW (UNIPROT) and were assumed to be full size proteins (INTACT). An additional 29 spots corresponded to MW that exceeded the predicted MW by at least 15% and were assumed to include protein modifications, dimers and aggregates (AGG). The remaining 45 spots were at least 15% below the predicted MW and were assumed to be protein fragments and peptides (FRAG).

### Subjects

As previously reported in detail, LBM significantly decreased in CON, remained near/below baseline in PEX, and increased in TEX. Conversely, FM increased in both CON and PEX and remained near/below baseline in TEX [3]. HDBR resulted in decreased strength while exercise with or without testosterone countermeasures were protective against such losses in load bearing muscles [3].

### Pre to post HDBR changes

Within-group abundance changes of all identified spots (pre-post, 2-tailed, paired t-tests) are shown in **Table 1**. Ordering in the table was based on Proteoform interpretation (i.e. intact, aggregate, fragment) and p-values of pre-post changes in CON. In CON, HDBR induced significant changes in the abundances of 17 intact proteoforms, 5 aggregates, and 4 fragments. The intact proteins included structural proteins (ANKRD2, ACTN2), Ca regulation and contractile proteins (TPM2, MYH2 (2 spots), BEST3 (2 spots), ACTG2), metabolic regulators (AK1, HSPB7, EEF1A1, HSPB1, TRIM72, DLD), and transport proteins (CA3, HBA1, HBB). The number of proteoforms that were significantly altered during HDBR were lower in PEX (6 intact, 5 aggregates, 3 fragments) and TEX (9 intact, 4, aggregates, 3 fragments) when compared to CON. Two of the proteins that underwent abundance changes in CON also changed in TEX. HSPB7 abundance went down in both CON and TEX, while it significantly increased in PEX. HBA1 increased in CON and TEX but underwent no significant change in PEX during HDBR. Testosterone has known erythropoietic properties, however, it remains unclear whether findings of increased HBA/HBB were physiologically relevant to skeletal muscle metabolism since circulating hematocrit did not change during the course of this study [3].

Spots that were differentially affected in response to countermeasures during HDBR included several structural and Ca regulation/contractile proteoforms as well as a few metabolic and transport proteins. Radixin (RDX) increased while TPM2 decreased respectively in PEX and TEX but not in CON. ACADVL and DES increased in PEX but not in TEX or CON. S100A13 increased in TEX but not in PEX or CON. MB, MYL1, and MYH7 each decreased in TEX but not in PEX or CON. Two spots representing ACTA1 were differentially affected in PEX and TEX respectively. ACTA1 (P68133, 41kD) increased in TEX but did not change in PEX or CON, while ACTA1 (Q5T8M7, 44kD) decreased in PEX but this change failed to reach significance in CON or TEX.

Changes in the phosphorylated proteoforms within each group (pre-post) are shown in **Table 2**. Significant HDBR-induced changes in phosphorylation status were observed in CON for 10 intact, 2 aggregate, and 11 fragment protein spots. The intact proteoforms included structural proteins (ANKRD2, MYBPC1), contractile proteins (ACTA1, TPM1), metabolic proteins (DLD, ACADVL), and transport proteins (ALB, CA3, HBA1, HBB). In TEX, 16 intact, 4 aggregate, and 5 fragment spots were significantly altered. Similar to changes during HDBR in CON, phosphorylated DLD and CA3 increased in TEX. The number of alterations in phosphorylated spots was lowest among the PEX group which included 7 intact, 3 aggregate, and 5 fragment proteoforms. Among the intact proteins, phosphorylated ALB increased in PEX, opposite to that observed in CON.

### Differences in PEX vs TEX

The pre-post effects of the two countermeasure groups (PEX vs TEX) were compared to provide insight on the influence of testosterone treatment vs. the underlying effects of exercise on proteomic responses during HDBR. Comparisons of the protein abundances between PEX and TEX subjects before (pre) and after (post) HDBR are shown in **Table 3**. Ordering in the table was based on proteoform interpretation (i.e. intact, aggregate, fragment) and p-values of post differences between PEX compared to TEX (positive values reflect higher expression in PEX compared to TEX). The abundances of 7 intact proteoforms, 5 aggregates, and 4 fragments were found to differ between PEX and TEX following HDBR. The intact proteins included structural proteins DES (2 spots) and RDX, oxygen transport proteins HBB (2 spots), HBD, and ALDOA, a glycolytic enzyme integral to the function and structure of the sarcoplasmic reticulum [12, 13]. In contrast, at baseline there were 3 intact, 6 aggregates, and 6 fragment spots found to be different between PEX and TEX. The intact proteins included ALB (transport protein), MYBPC1 (structural protein), and MYL1 (contractile protein), none of which were found to be different between the two exercise groups following HDBR.

Comparisons of protein phosphorylation status between PEX and TEX subjects before (PRE) and after (post) HDBR are shown in **Table 4**. While there were only a few pre-HDBR differences in phosphorylated proteoforms between PEX and TEX (1 intact, 1 aggregate, and 3 fragment), this difference increased to 10 intact, 3 aggregate, and 4 fragment proteoforms post-HDBR. The intact proteins that were significantly different (all higher in PEX following HDBR when compared to TEX) included contractile proteins (MYH1, MYH2 (2 spots), ACTA1 (2 spots), TNNC2) and metabolic proteins (CKM (3 spots) and ALDOA).

### Principal Components Analyses (PCA)

**Fig 2:**
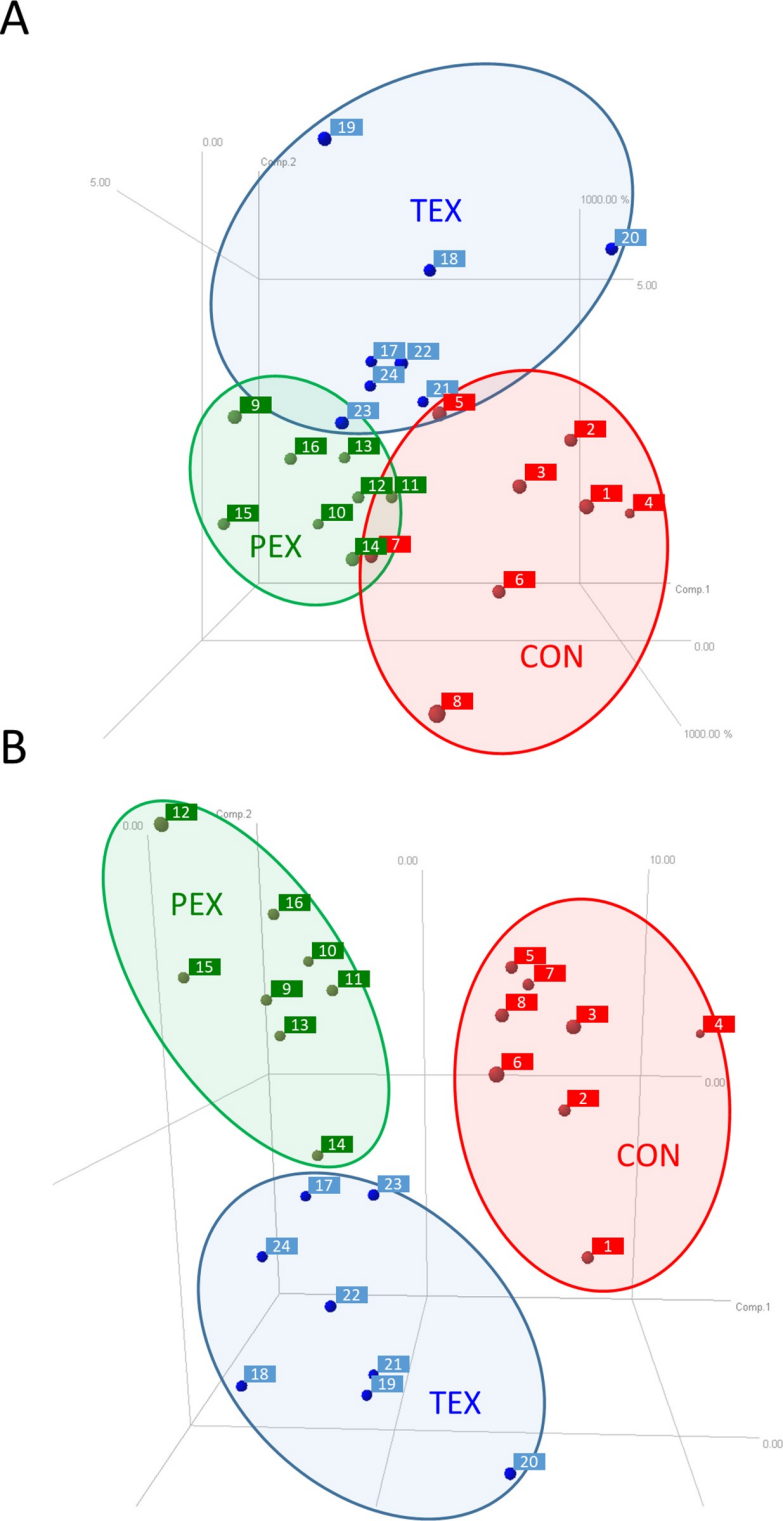
Principal Components Analyses (PCA). Principal Components Analysis demonstrating post-HDBR differences in the proteomes of CON (red, subjects 1–8), PEX (green, subjects 9–16), and TEX (blue, subjects 17–24). Spots are numbered for consistent comparison of data from individual subjects between figures throughout the manuscript. **(A)** PCA based on post-HDBR differences in protein abundance. **(B)** PCA based on post-HDBR differences in protein phosphorylation.

Results from the PCA performed on post-HDBR data are shown in **Fig 2**. The purpose of this analysis was to reveal patterns of protein expression/phosphorylation in the post data that may distinguish the CON, PEX, and TEX sample groups from one another. For abundance PCA, the spot intensities of the 71 gel spots that showed significant expression difference among these three sample groups were used as input. **Fig 2A** is the resulting PCA1-PCA2-PCA3 plot. The 24 dots in the figure represent the eight CON (red), eight PEX (green), and eight TEX (blue) samples. Notably, there is distinct clustering among the groups, with the three sample groups occupying separable, non-overlapping, regions in the PCA plot. The results of a similar PCA, based on the 81 gel spots exhibiting significant phosphorylation differences is depicted in **Fig 2B**. The color coding of the 24 spots is the same as for the abundance PCA and the clustering of the three sample groups is even more evident for the phosphorylation PCA. The variability in data captured by PCA along PC1 and PC2 were 39% and 10% respectively in **Fig 2A**, and were 30% and 12% respectively in **Fig 2B**. While these numbers are moderate, the clear separation among CON, TEX, and PEX observed in the plots (which is especially noticeable in **Fig 2B**, the phosphorylation based-plot) suggests that, by the end of the study, there were clear differences in the patterns of protein expression/phosphorylation among the participant groups that underwent just bed rest (CON), exercise (PEX), and exercise + testosterone (TEX) treatment.

### Pathway analyses

**Fig 3:**
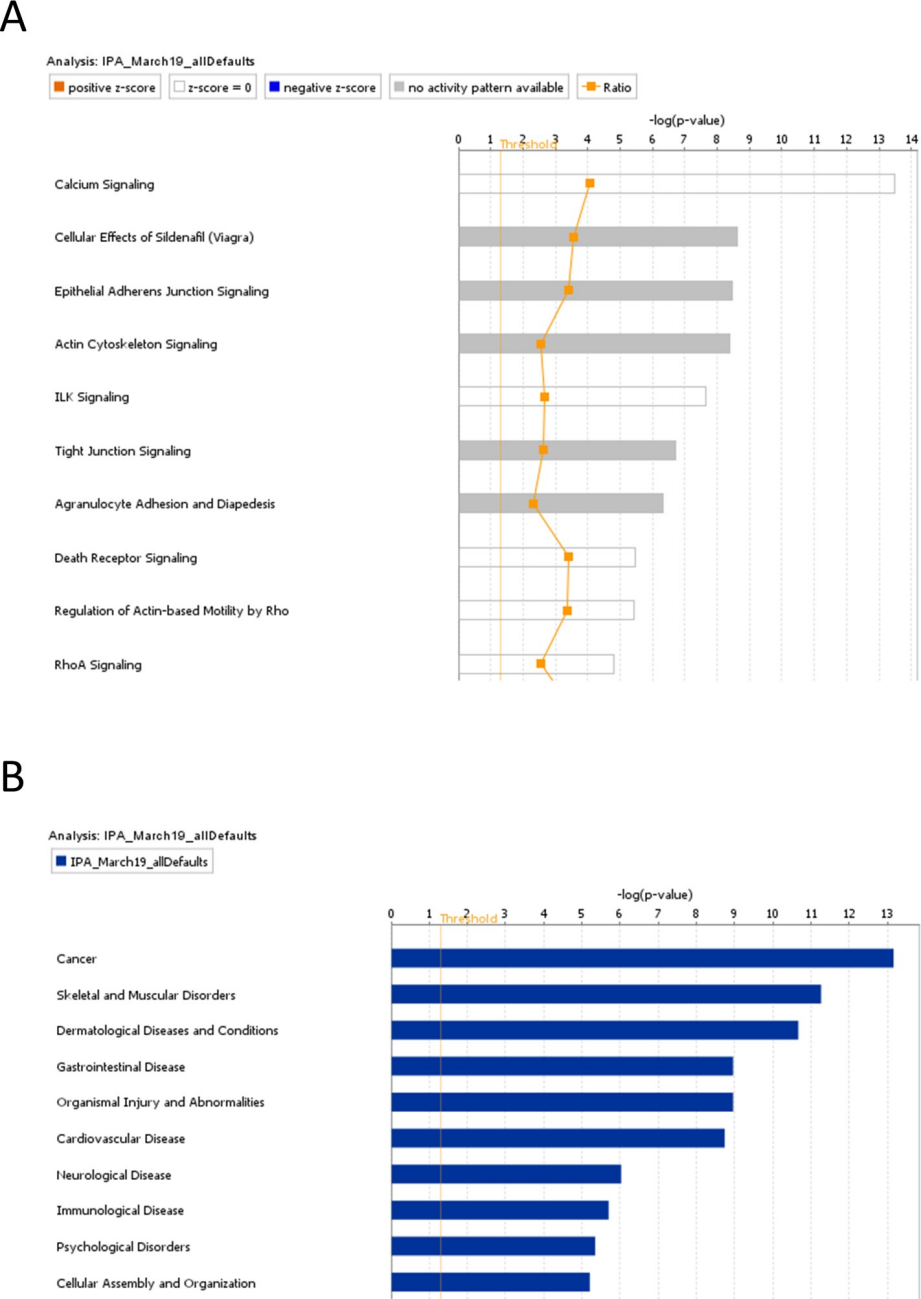
Ingenuity Pathway Analysis (IPA). **(A)** Top pathways identified by preliminary IPA based on differential changes in protein abundances and phosphorylation in response to ~70 days of HDBR with or without countermeasures. **(B)** Top associated pathologies identified by IPA based on differential changes in protein abundances and phosphorylation in response to ~70 days of HDBR with or without countermeasures.

HDBR resulted in proteomic changes within skeletal muscle and both countermeasures caused differential changes during long term HDBR. Major canonical signaling networks were identified through Ingenuity Pathway Analysis (IPA) as playing a role in the differential regulation of skeletal muscle in response to HDBR in the presence or absence of applied exercise and placebo or exercise and testosterone countermeasures. Several pathways such as Calcium Signaling, Cellular Effects of Sildenafil (Viagra), Epithelial Adherens Junction Signaling, Actin Cytoskeleton Signaling, and ILK Signaling can be associated with those signaling networks (**Fig 3**). Similar analyses revealed that the biological functions most likely impacted included Cellular Assembly and Organization, Cellular Function and Maintenance, Cell Death and Survival, Carbohydrate Metabolism, Molecular Transport, Organ Morphology, Tissue Development, Behavior, Cardiovascular System Development and Function, and Skeletal and Muscular System Development and Function. Finally, IPA associated a number of known pathologies with the observed changes, including musculoskeletal, dermatological, gastrointestinal, cardiovascular, neurological, immunological, and psychological disorders.

Accession numbers of proteoforms that showed significant pre-post HDBR changes were also submitted for gene ontology enrichment analyses of individual groups (http://www.geneontology.org) (see **S1 Table, gene ontology**). For instance, confinement to HDBR (CON) altered abundances of proteins related to biological process of muscle contraction (MYH2, ACTG2, TPM2, TRIM72, ANKRD2), consistent with the previously published losses in muscle mass and strength in this group [3]. Exercise countermeasures (PEX) uniquely affected cellular organization in skeletal muscle (ACTA1, TPM2, TPM1, KRT9, DES) while the addition of testosterone (TEX) affected mesenchymal migration proteins (ACTA1, ACTG2, ACTC1), which would be consistent with increased myogenesis [14] and previously published increases in lean body mass in the TEX group [3]. Thus, the countermeasures resulted in considerable modulation of the networks and pathways identified in our proteomic analyses and show overlap with known physiological responses that occur on Earth and during space flight.

### Prediction models

**Fig 4:**
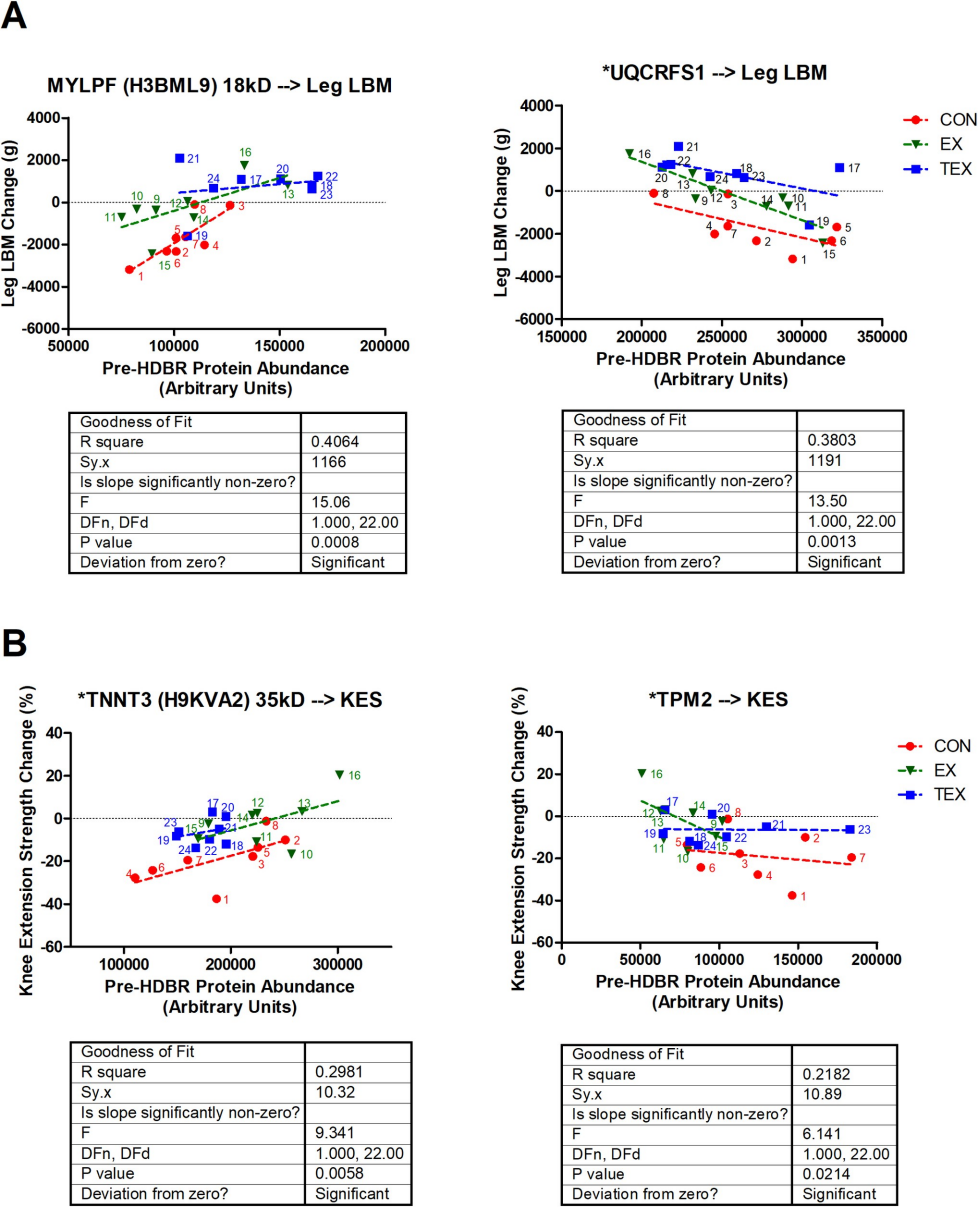
Prediction model. **(A)** Baseline (Pre-HDBR) protein abundance levels of Myosin regulatory light chain 2 (MYLPF) in the vastus lateralis plotted against PRE-post changes in Leg LBM. There were no relationships between baseline Leg LBM and protein abundances (not shown). **(B)** Baseline (Pre-HDBR) protein abundance levels of Troponin T Type3 (TNNT3) in the vastus lateralis plotted against PRE-post changes in knee extension strength (KES). There were no relationships between baseline strength and protein abundances (not shown).

Linear regression analyses using the discovery proteomics data identified several potentially predictive biomarkers for the individual subject susceptibility to HDBR and the effectiveness of countermeasures to prevent losses in lean leg mass and knee extension strength. There were significant (P < 0.05) relationships found between baseline (i.e., pre-HDBR) abundance levels of Myosin regulatory light chain 2, skeletal muscle isoform (fragment) (MYLPF H3BML9 (18kD); regulator of muscle contraction) (**Fig 4A**), Ubiquinol-cytochrome c reductase complex (UQCRFS1 P47985 (53kD); mitochondrial respiratory chain protein involved in energy metabolism), Adenylate kinase isoenzyme (AK1 P00568 (24kD); cytosolic protein involved in skeletal muscle ATP metabolism and energy homeostasis), and Desmin (DES P17661 (50kD); an intermediate filament protein) vs. subsequent changes in lean leg mass during HDBR. Baseline expression of Troponin T Type3, fast skeletal muscle type (TNNT3 H9KVA2 (35kD); anchoring protein necessary for skeletal muscle contraction) (**Fig 4B**), Ubiquinol-cytochrome c reductase complex (UQCRFS1 P47985 (53kD); energy metabolism), Actin A (ACTA1 P68133 (41kD) & ACTA1 Q5T8M7 (42kD); a muscle specific structural microfilament), and a 159 kD proteoform identified as an aggregate of Tropomyosin beta chain (TPM2 P07951 (159kD); a calcium induced contractile regulator expected at 32 kD) in the *vastus lateralis* were predictive of changes in strength performance of the relevant large muscle group (*quadriceps femoris*) during HDBR.

## Discussion

The present study demonstrated notable alterations in the skeletal muscle proteome of healthy men in response to skeletal muscle unloading under extremely well-controlled long-term HDBR conditions. In addition, these changes were compared to the alterations that occur when exercise countermeasures with or without testosterone supplementation were included as part of the HDBR condition. We previously reported on the efficacy of these countermeasures in protecting against muscle atrophy [3]. Exercise was largely successful in protecting against HDBR-induced strength loss while the addition of testosterone to exercising subjects resulted in an accumulation of lean body mass. The addition of this proteomic investigation supplemented the existing data by identifying several proteins with known structural, contractile, or metabolic functions to be altered in response to bed rest, exercise, and/or testosterone. Overall, our results corroborate findings by others [15–18] that long duration bed rest results in changes in the abundances and post-translational phosphorylation of proteins with structural, contractile, and metabolic functions.

### Structural changes

Confinement to bed rest is known to induce a downregulation of structural proteins [15, 16]. Likewise in this study, long duration HDBR resulted in decreased abundances and/or phosphorylation of several structural proteins (ANKRD2, ACTN2, MYBPC1) in CON, which was not observed in the countermeasure groups PEX and TEX. Conversely, high intensity and/or eccentric exercise protocols are known to promote synthesis of structural proteins such as DES as well as ERM (Ezrin, RDX and Moesin) proteins [19, 20]. The ERM proteins play an important role in signal transduction between the extracellular matrix and the cytoskeletal proteins that maintain cell structure [21] and are susceptible to gravitational stresses *in vitro* [22, 23]. In the current study, DES significantly increased in PEX which resulted in higher post-HDBR abundances compared to TEX while increases in RDX abundances in TEX subjects outpaced the increase observed in PEX subjects, resulting in higher post-HDBR expression among TEX subjects. Overall, these findings confirm the published data demonstrating benefits of exercise and testosterone countermeasures on protecting against losses of muscle mass in these subjects during long duration HDBR [3, 4].

### Contractile changes

Bed rest induced alteration in abundances of contractile proteins were consistent with those reported elsewhere. For instance, increased tropomyosin (TPM2) and decreases in smooth muscle actin ACTG2 were previously reported in response to 60 days of bed rest [17]. Decreases in MYH2 abundances in the present report, along with unaltered abundances in MYH1, are also consistent with findings described during other bed rest studies [15, 17, 24, 25]. However, this direction of change is in contrast to the expected modest slow-to-fast transition from MYH1 towards MYH2a fibers in *vastus lateralis* reported by our colleagues [26], This disparity may be due to methodological differences between comparing ratios of fibers identified by the predominant protein type vs. measuring protein abundances in total sample homogenates. Thus, the decrease in myosin protein abundance may be more reflective of overall losses in skeletal muscle protein than of shifts in skeletal muscle fiber type.

Changes in abundances and phosphorylation of contractile protein abundances were also observed in the PEX and TEX exercise groups. Despite comparable responses in muscle strength between the exercise groups [3], phosphorylation of several contractile proteins were higher in PEX compared to TEX after HDBR. Specifically, phosphorylation of MYH1 and MYH2 tended to increase in PEX while (statistically nonsignificant) declines in TEX were observed. Similarly, opposite pre-post HDBR changes were observed in the phosphorylation of thin filament proteins ACTA1 and TNNC2 between PEX and TEX. post-HDBR phosphorylation among PEX was consistently higher for these proteins than that among TEX (**Table 4**). Phosphorylation of the contractile protein troponin 2 (TNNC2) decreased in TEX and tended to increase in PEX while no consistent changes were observed in spots related to myosin regulatory light chain (MYLPF).

Regulation of contractile proteins through phosphorylation is complex and it is difficult to relate the changes observed in the skeletal muscle proteome to the functional changes observed in the subjects. For instance, phosphorylation of myosin 1 heavy chain is important during cellular organization but does not affect the strength of its interaction with actin [27]. In contrast, phosphorylation of contractile proteins such as myosin regulatory light chain and troponin plays a role in maintaining Ca^2+^ sensitivity and improve force production, especially under suboptimal Ca concentrations [28, 29]. The mechanisms behind the differences in phosphorylation of the mentioned contractile proteins among exercising groups during HDBR remain unclear but could be related to a number of factors. Among the possibilities is that the need for cellular reorganization and functional optimization in response to exercise alone (PEX) was partially offset by a drive towards hypertrophy and generation of new muscle tissue in the subjects receiving testosterone (TEX). Abundance and phosphorylation of S100A13 increased in TEX. This protein family has been shown to increase in response to estrogens as well as androgens in various tissues and are regarded as early response genes involved in regulation of tissue growth, angiogenesis and inflammatory responses [30, 31]. The consistently higher level of phosphorylation among contractile proteins in PEX may be indicative of increased cellular restructuring and protein turnover in response to exercise induced mechanical stresses [32]. In contrast, administration of testosterone may have blunted this exercise-induced protein turnover and catabolism [33] contributing to the net increases in lean mass and protection of muscle strength in TEX.

### Metabolic changes

Phosphorylation of ALDOA, a triad-associated protein involved in Ca^+2^ regulation and integral to excitation-contraction coupling of skeletal muscle [13], tended to decline in PEX and TEX. post-HDBR abundance as well as phosphorylation of this glycolytic enzyme were lower among TEX compared to PEX. Androgen induced downregulation of ALDOA has been reported in adipose tissue and this corresponds to a repression of pyruvate synthesis and decreased lipogenesis [34].

Abundance of ACADVL, a mitochondrial enzyme which catalyzes the first step in the beta oxidation pathway, increased in response to exercise (PEX) but not in testosterone treated subjects (TEX). Interestingly, HDBR alone (CON) resulted in increases in phosphorylated ACADVL. The mechanism behind this shift in the CON subjects is unclear as increased abundance of ACADVL has previously been associated with responses to exercise training [16, 20, 35]. Increased abundance (PEX) and/or activation by phosphorylation of ACADVL (CON) during HDBR may have been responses to offset shifts towards increased buildup of intracellular lipids [36, 37] and could be consistent with the increases in fat mass observed in these subjects [3]. Furthermore, HDBR has been reported to result in decreases in mitochondrial enzymes such as DLD [16], which is consistent with our results in CON. Although prevented by resistive vibration exercise (RVE) countermeasures [16], the exercise and testosterone countermeasures in the present study did not inhibit the decline in DLD. Similarly, there were no overt changes in the abundance of the energy transduction protein CKM, although phosphorylation of CKM declined in PEX as well as TEX during HDBR (**Table 3**). This decline was more profound in the TEX group resulting in significant post-HDBR differences between PEX and TEX (**Table 4**). Interestingly, the decline in phospho-CKM in TEX was somewhat offset by an increase in phosphorylation of the alternate ATP producing kinase, AK1 (P = 0.056, **Table 3**).

### Prediction models

In addition to demonstrating that HDBR results in alterations in the skeletal muscle proteome that may be modulated by exercise and testosterone countermeasures, we identified a subset of proteins that appeared predictive of HDBR-induced changes in muscle mass and strength. Discovery of sensitive proteomic biomarkers may in the future allow for personalized medicine approaches by aiding in the development of more directed countermeasures based upon baseline proteome profiles. Our data identified several proteins at baseline that showed strong correlation with subsequent changes in muscle mass and strength during HDBR. These proteomic biomarkers offer a good potential for prediction of HDBR induced changes in body composition or strength (**Fig 4)**. While countermeasures improved lean body mass and strength during HDBR compared to control, baseline abundances of these skeletal muscle proteins were predictive of the outcomes. Visually, the linear regression analyses lead to interesting interpretations when the pre-post responses within each countermeasure group are compared between the groups. For instance, in **Fig 4A**, low abundance of the 18kD proteoform for MYLPF in skeletal muscle tissue collected before bed rest was a strong predictor for the quantity of LBM lost during HDBR, especially in absence of countermeasures. Furthermore, these data suggest that exercise alone may be an excellent countermeasure for individuals with relatively mid to high baseline levels of 18kD MYLPF, and that exercise + testosterone may be effective in improving LBM independent of baseline levels of these proteins. Baseline levels of TNNT3 in *vastus lateralis* were good predictors of changes in knee extension strength during HDBR while exercise with or without testosterone countermeasures provided an upward shift in protection against loss of strength (**Fig 4B**). Subjects with best cases in terms of knee extension strength changes during HDBR were among those with highest pre-HDBR abundances in TNNT3.

Identifying individuals as either responders or non-responders to the effects of HDBR and/or countermeasures is complex and depends on a myriad of factors including, but not limited to, the individual’s susceptibility to HDBR induced muscle atrophy as well as the predicted effect of the countermeasures. As expected, no individual protein we identified was fully predictive for every subject or physiological function measured, and the development of accurate predictive models will likely involve algorithms that include panels of several proteins and factors. However, our simple approach illustrates the potential use of this predictive model for identifying the responses to either HDBR and/or countermeasures in individual subjects (**Fig 4**). For example, subject # 1 (CON) was among the individuals with the most severe muscle atrophy in terms of both mass as well as strength. Conversely, the pre-bed rest abundance levels of 3 out of the 4 proteins depicted in **Fig 4** were good predictors as this subject was consistently towards the low end of the regression-line in the absence of countermeasures. Subject # 11 (PEX) was a good responder to the exercise countermeasure. Pre-HDBR data would have predicted losses in muscle mass and strength akin to those observed in subject #1 (CON) but subject #11 repeatedly performed better, consistent with the upward shifted lines in **Fig 3A–3D**. Subjects # 17 and #21 (TEX) were good responders to the testosterone + exercise countermeasure and these subjects consistently performed better than predicted by pre-HDBR proteomics data. Interestingly, subject 16 (PEX) was among the greatest gainers of muscle mass and strength. This subject was among the exercisers, although his data were consistently towards the upper end of the regression-line suggesting that even in the absence of countermeasures this subject might have been among those with the lowest severity of muscle atrophy.

While this study was not originally designed or powered for this type of investigation, the prediction modeling exercise was undertaken as proof-of-concept to probe for possible factors associated with individual variations in responses to countermeasures among the test subjects. We propose that our data may serve as a prelude to methods that utilize the baseline proteome in a personalized medicine approach to aid in the prediction of health and performance risks in response to the absence or presence of countermeasures. Further development of such methods could have clinical applications and would specifically help NASA and other agencies shape personalized prescriptions of countermeasures for crewmembers to follow during long duration space flight missions.

A potential clinical limitation of the current study is that it did not include a non-exercising group that received testosterone during HDBR. The addition of such a group was included in the early design of this study. However, a control + testosterone group was ultimately downselected during discussions with the NASA human research program (HRP) and the other investigative teams included in this bed rest campaign as it was deemed that such a group was of less operational interest to NASA given that exercise countermeasure protocols will continue to be high priority for all astronauts during flight. Inclusion of a non-exercise (CON) group remained a high priority for the complement of selected investigators and stakeholders that were participating in this bed rest campaign. Thus, the selected countermeasures for evaluation during this investigation included the Sprint exercise protocol and a combined Sprint + testosterone protocol vs. standard HDBR control conditions.

## Conclusion

In summary, long-duration HDBR results in numerous proteomic alterations spanning a range of biological functions that are blunted or reversed by the addition of exercise countermeasures. During HDBR, exercise appeared to drive cellular reorganization in skeletal muscle while the addition of testosterone blunted catabolism and induced overall skeletal muscle hypertrophy. This investigation demonstrated that the inclusion of a low dose testosterone countermeasure partially modulates the effects of exercise providing a unique insight into the differential mechanical and biochemical regulators of muscle proteins during HDBR. Furthermore, the baseline proteomic data offered important insight and its potentially applicability as a powerful tool to predict changes in muscle mass and strength, and the effectiveness of exercise and hormonal countermeasures. Knowledge of the individual physiological susceptibility to functional declines during unloading may help tailor effective countermeasure strategies to the individual astronaut prior to embarking on a space flight mission.

## Supporting information

S1 Table Gene ontology. Proteoforms that showed significant pre-post abundance changes i**n Table 1** were submitted for Enrichment Analyses (Annotation Version and Release Date: GO Ontology database Released 2018-08-09, http://www.geneontology.org/,). Table include only results with False Discovery Rate < 0.05. A. CON, B. PEX, C. TEX.
(PDF)

S1 File Proteoform analyses. Raw protein abundance and phosphorylation data from MS Analyses. These data were submitted to the NASA Life Science Data Archive (https://lsda.jsc.nasa.gov/).
(XLSX)

S2 File Study protocol. The study complied with the Declaration of Helsinki and was approved by The University of Texas Medical Branch (UTMB) Institutional Review Board (IRB) and by the NASA Committee for the Protection of Human Subjects (CPHS).
(PDF)

S3 File CONSORT checklist. This study adheres to CONSORT guidelines.
(DOC)
