# Supplementary material for: Proteomic investigation of human skeletal muscle before and after 70 days of head down bed rest with or without exercise and testosterone countermeasures
Source: PLoS One. 2019 Jun 13;14(6):e0217690. doi: 10.1371/journal.pone.0217690 (PMC6563988; doi:10.1371/journal.pone.0217690)
Supplement: S1 Table — Proteoforms that showed significant pre-post abundance changes in Table 1 were submitted for Enrichment Analyses (Annotation Version and Release Date: GO Ontology database Released 2018-08-09, http://www.geneontology.org/,). Table include only results with False Discovery Rate < 0.05. A. CON, B. PEX, C. TEX. (PDF) [file pone.0217690.s001.pdf]

S1 Table, Gene Ontology. Proteoforms that showed significant PRE-POST abundance changes in Table 1 were submitted for Enrichment Analyses (Annotation Version and Release Date: GO Ontology database Released 2018-08-09, http://www.geneontology.org/,). Tables below include only results with False Discovery Rate < 0.05. A. CON, B. PEX, C. TEX

A. CON: P00568, Q9UBY9, P07451, *F8VVX2*, P07951, Q9UKX2, *F8VVX2*, Q9GZV1, P68104, P69905, P04792, Q6ZMU5, P68871, Q9UKX2, P63267, *E9PEX6*, *B2RCS5*, *F8VVX2*, P69905, *F8VVX2*, P69905, P69905, P35908, P16219, P69905, P31415, *F8WCL5* (*Unmapped*)

BIOLOGICAL PROCESS

Homo sapiens GO biological process complete	#	#	expected	Fold Enrichment	+/-	raw P value	FDR
bicarbonate transport	36	3	.03	> 100	+	2.63E-06	1.38E-02
muscle contraction	238	5	.17	29.47	+	5.37E-07	8.45E-03
↳muscle system process	285	5	.20	24.61	+	1.28E-06	1.01E-02

MOLECULAR FUNCTION

No statistically significant results.

CELLULAR COMPONENT

Homo sapiens GO cellular component complete	#	#	expected	Fold Enrichment	+/-	raw P value	FDR
haptoglobin-hemoglobin complex	10	2	.01	> 100	+	3.11E-05	5.74E-03
hemoglobin complex	11	2	.01	> 100	+	3.68E-05	6.21E-03
↳cytosol	5077	11	3.62	3.04	+	8.20E-05	1.04E-02
↳cytoplasmic part	9646	14	6.88	2.04	+	2.56E-04	2.26E-02
↳cytoplasm	11495	15	8.19	1.83	+	1.23E-04	1.38E-02
endocytic vesicle lumen	18	2	.01	> 100	+	8.93E-05	1.07E-02
↳organelle part	9480	14	6.76	2.07	+	1.24E-04	1.32E-02
↳intracellular organelle part	9183	14	6.55	2.14	+	8.13E-05	1.10E-02
myosin filament	22	2	.02	> 100	+	1.30E-04	1.25E-02
↳actin cytoskeleton	490	5	.35	14.31	+	1.74E-05	3.52E-03
↳cytoskeleton	2168	8	1.55	5.18	+	4.24E-05	6.60E-03
↳intracellular non-membrane-bounded organelle	4197	11	2.99	3.68	+	1.23E-05	3.10E-03
↳non-membrane-bounded organelle	4204	11	3.00	3.67	+	1.25E-05	2.81E-03
↳cytoskeletal part	1673	7	1.19	5.87	+	7.37E-05	1.07E-02
I band	139	3	.10	30.28	+	1.29E-04	1.30E-02
↳sarcomere	196	5	.14	35.79	+	2.10E-07	4.25E-04
↳myofibril	217	5	.15	32.32	+	3.43E-07	2.32E-04
↳contractile fiber	231	5	.16	30.36	+	4.64E-07	2.35E-04
↳supramolecular fiber	958	7	.68	10.25	+	1.93E-06	7.84E-04
↳supramolecular polymer	965	7	.69	10.18	+	2.03E-06	6.86E-04
↳supramolecular complex	966	7	.69	10.17	+	2.04E-06	5.92E-04

↳contractile fiber part	214	5	.15	32.78	+	3.21E-07	3.25E-04
blood microparticle	143	3	.10	29.43	+	1.40E-04	1.29E-02
extracellular exosome	2095	7	1.49	4.69	+	3.05E-04	2.58E-02
↳extracellular vesicle	2115	7	1.51	4.64	+	3.24E-04	2.62E-02
↳extracellular organelle	2117	7	1.51	4.64	+	3.26E-04	2.54E-02

B. PEX: Q9UBY9, *Q5T8M7*, P35241, P07951, P49748, P17661, P07951, *H9KVA2*, *G3V3T0*, P09493, P68133, *H3BQ84*, P35527 (*Unmapped*)

BIOLOGICAL PROCESS

Homo sapiens<br>GO biological process complete	#	#	expected	Fold Enrichment	+/-	raw P value	FDR
muscle filament sliding	38	4	.01	> 100	+	9.52E-10	7.49E-06
↳actin-myosin filament sliding	38	4	.01	> 100	+	9.52E-10	1.50E-05
↳actin-mediated cell contraction	84	4	.03	> 100	+	1.97E-08	1.03E-04
↳actin filament-based movement	105	4	.04	> 100	+	4.69E-08	1.84E-04
↳actin filament-based process	540	4	.21	19.48	+	2.84E-05	4.97E-02
↳muscle contraction	238	4	.09	44.21	+	1.15E-06	3.02E-03
↳muscle system process	285	4	.11	36.92	+	2.33E-06	5.24E-03
supramolecular fiber organization	443	5	.17	29.69	+	2.27E-07	7.14E-04
cytoskeleton organization	1067	5	.41	12.33	+	1.67E-05	3.29E-02

MOLECULAR FUNCTION

Homo sapiens<br>GO molecular function complete	#	#	expected	Fold Enrichment	+/-	raw P value	FDR
structural constituent of cytoskeleton	104	4	.04	> 100	+	4.51E-08	2.10E-04
↳structural molecule activity	815	5	.31	16.14	+	4.50E-06	6.99E-03
cytoskeletal protein binding	937	6	.36	16.84	+	2.06E-07	4.80E-04

CELLULAR COMPONENT

Homo sapiens<br>GO cellular component complete	#	#	expected	Fold Enrichment	+/-	raw P value	FDR
muscle thin filament tropomyosin	4	2	.00	> 100	+	1.89E-06	4.27E-04
↳contractile fiber part	214	4	.08	49.16	+	7.58E-07	3.07E-04
↳contractile fiber	231	4	.09	45.55	+	1.02E-06	2.59E-04
↳supramolecular fiber	958	5	.36	13.73	+	9.90E-06	1.34E-03
↳supramolecular polymer	965	5	.37	13.63	+	1.03E-05	1.30E-03
↳supramolecular complex	966	5	.37	13.61	+	1.03E-05	1.23E-03
↳intracellular non-membrane-bounded organelle	4197	8	1.60	5.01	+	2.52E-06	5.11E-04
↳non-membrane-bounded organelle	4204	8	1.60	5.01	+	2.56E-06	4.71E-04
↳striated muscle thin filament	31	3	.01	> 100	+	2.14E-07	4.35E-04
↳actin cytoskeleton	490	5	.19	26.84	+	3.72E-07	2.52E-04
↳cytoskeleton	2168	7	.82	8.49	+	9.07E-07	2.63E-04
↳myofilament	32	3	.01	> 100	+	2.34E-07	2.38E-04
↳myofibril	217	4	.08	48.48	+	8.01E-07	2.71E-04
↳sarcomere	196	4	.07	53.68	+	5.37E-07	2.72E-04
↳cytoskeletal part	1673	6	.64	9.43	+	6.21E-06	9.68E-04
stress fiber	61	2	.02	86.24	+	2.44E-04	2.60E-02
↳actomyosin	74	2	.03	71.09	+	3.55E-04	3.27E-02
↳contractile actin filament bundle	61	2	.02	86.24	+	2.44E-04	2.75E-02
↳actin filament bundle	68	2	.03	77.36	+	3.01E-04	3.05E-02
actin filament	105	3	.04	75.15	+	7.22E-06	1.05E-03
↳polymeric cytoskeletal fiber	736	5	.28	17.87	+	2.73E-06	4.62E-04
cell leading edge	394	3	.15	20.03	+	3.47E-04	3.35E-02

C. TEX: Q9UBY9, P69905, P35241, Q99584, P02144, P05976, P07951, P68133, P12883, P69905, P68133, P11217, P68133, P68032, *H0Y390*, P63267 (*Unmapped*)

BIOLOGICAL PROCESS

Homo sapiens<br>GO biological process complete	#	#	expected	Fold Enrichment	+/-	raw P value	FDR
mesenchyme migration	5	3	.00	> 100	+	7.91E-09	1.78E-05
↳tissue migration	95	3	.05	55.37	+	2.09E-05	2.35E-02
↳mesenchyme morphogenesis	48	3	.03	> 100	+	2.90E-06	5.07E-03
oxygen transport	14	2	.01	> 100	+	3.56E-05	3.50E-02
skeletal muscle thin filament assembly	14	2	.01	> 100	+	3.56E-05	3.29E-02
↳actin filament-based process	540	5	.31	16.24	+	7.77E-06	1.22E-02
↳skeletal myofibril assembly	16	2	.01	> 100	+	4.53E-05	3.96E-02
muscle filament sliding	38	5	.02	> 100	+	2.19E-11	1.15E-07
↳actin-myosin filament sliding	38	5	.02	> 100	+	2.19E-11	1.72E-07
↳actin-mediated cell contraction	84	5	.05	> 100	+	9.32E-10	2.93E-06
↳actin filament-based movement	105	5	.06	83.50	+	2.73E-09	7.17E-06
↳muscle contraction	238	7	.14	51.57	+	2.00E-11	3.15E-07
↳muscle system process	285	7	.16	43.07	+	6.86E-11	2.70E-07
↳system process	1890	7	1.08	6.49	+	2.51E-05	2.64E-02
cardiac muscle contraction	74	3	.04	71.09	+	1.01E-05	1.45E-02
↳striated muscle contraction	103	4	.06	68.10	+	3.02E-07	5.95E-04
↳heart contraction	84	3	.05	62.63	+	1.46E-05	1.91E-02
↳heart process	95	3	.05	55.37	+	2.09E-05	2.53E-02

MOLECULAR FUNCTION

Homo sapiens<br>GO molecular function complete	#	#	expected	Fold Enrichment	+/-	raw P value	FDR
oxygen carrier activity	13	2	.01	> 100	+	3.11E-05	4.84E-02
cytoskeletal protein binding	937	6	.53	11.23	+	5.82E-06	2.71E-02
drug binding	1751	7	1.00	7.01	+	1.52E-05	3.54E-02

CELLULAR COMPONENT

Homo sapiens<br>GO cellular component complete	#	#	expected	Fold Enrichment	+/-	raw P value	FDR
muscle myosin complex	18	2	.01	> 100	+	5.63E-05	5.43E-03
↳contractile fiber part	214	5	.12	40.97	+	8.68E-08	5.87E-05
↳cytoplasm	11495	12	6.56	1.83	+	7.84E-04	4.41E-02
↳contractile fiber	231	5	.13	37.95	+	1.26E-07	5.11E-05
↳supramolecular fiber	958	5	.55	9.15	+	1.20E-04	9.72E-03
↳supramolecular polymer	965	5	.55	9.09	+	1.24E-04	9.67E-03
↳supramolecular complex	966	5	.55	9.08	+	1.25E-04	9.36E-03
↳intracellular non-membrane-bounded organelle	4197	10	2.39	4.18	+	4.45E-06	8.20E-04
↳non-membrane-bounded organelle	4204	10	2.40	4.17	+	4.52E-06	7.64E-04
↳myosin II complex	26	2	.01	> 100	+	1.12E-04	9.43E-03
↳myosin complex	68	3	.04	77.36	+	7.91E-06	1.23E-03

↳actin cytoskeleton	490	8	.28	28.63	+	4.22E-11	8.56E-08
↳cytoskeleton	2168	8	1.24	6.47	+	4.34E-06	8.80E-04
↳cytoskeletal part	1673	7	.95	7.34	+	1.13E-05	1.52E-03
myosin filament	22	2	.01	> 100	+	8.16E-05	7.52E-03
striated muscle thin filament	31	2	.02	> 100	+	1.56E-04	1.09E-02
↳myofilament	32	2	.02	> 100	+	1.65E-04	1.12E-02
↳myofibril	217	5	.12	40.40	+	9.29E-08	4.71E-05
↳sarcomere	196	5	.11	44.73	+	5.66E-08	5.74E-05
filopodium	100	4	.06	70.14	+	2.70E-07	9.11E-05
↳actin-based cell projection	199	4	.11	35.25	+	3.91E-06	8.80E-04
stress fiber	61	2	.03	57.49	+	5.70E-04	3.50E-02
↳actomyosin	74	3	.04	71.09	+	1.01E-05	1.46E-03
↳contractile actin filament bundle	61	2	.03	57.49	+	5.70E-04	3.61E-02
↳actin filament bundle	68	2	.04	51.57	+	7.04E-04	4.08E-02
actin filament	105	3	.06	50.10	+	2.79E-05	3.54E-03
blood microparticle	143	4	.08	49.05	+	1.08E-06	3.13E-04
↳extracellular region part	3491	8	1.99	4.02	+	1.52E-04	1.10E-02
↳extracellular region	4302	8	2.45	3.26	+	6.84E-04	4.08E-02
↳extracellular space	3304	8	1.88	4.25	+	1.01E-04	8.92E-03
lamellipodium	189	4	.11	37.11	+	3.20E-06	8.11E-04
↳cell leading edge	394	4	.22	17.80	+	5.52E-05	5.60E-03
extracellular exosome	2095	7	1.19	5.86	+	4.93E-05	5.88E-03
↳extracellular vesicle	2115	7	1.21	5.80	+	5.24E-05	5.90E-03
↳extracellular organelle	2117	7	1.21	5.80	+	5.27E-05	5.63E-03
cytosol	5077	9	2.90	3.11	+	2.95E-04	1.93E-02
