# Supplementary material for: Proteomic investigation of human skeletal muscle before and after 70 days of head down bed rest with or without exercise and testosterone countermeasures
Source: PLoS One. 2019 Jun 13;14(6):e0217690. doi: 10.1371/journal.pone.0217690 (PMC6563988; doi:10.1371/journal.pone.0217690)
Supplement: S2 File — The study complied with the Declaration of Helsinki and was approved by The University of Texas Medical Branch (UTMB) Institutional Review Board (IRB) and by the NASA Committee for the Protection of Human Subjects (CPHS). (PDF) [file pone.0217690.s003.pdf]

Date: Sunday, June 19, 2011 4:57:37 PM Print Close

ID: Pro0028 View: 1.0 - Study Identification Information

1.0 - Study Identification Information

This is the first step in your Human Research Application. You will automatically be guided to the appropriate forms needed to complete your submissions.

1.0 * **Study Name:**

Testosterone Supplementation as a Countermeasure against Musculoskeletal Losses during Space Exploration

2.0 * **Brief Synopsis / Abstract:**

The long-term goal of this proposal is to determine the therapeutic efficacy of testosterone at preserving lean muscle mass, muscle strength, and bone mineral density in healthy humans during spaceflight. We propose to examine the interactive or additive effects of the combination of testosterone and exercise on lean body mass (LBM), muscle strength and bone health.

To achieve these goals we will test the effect of testosterone replacement during and after 70 days of bed rest:
Cycled testosterone replacement (weekly testosterone for two weeks, followed by two weeks off, etc.) in conjunction with exercise will have an additive effect in preventing loss of muscle mass, muscle strength, bone mass, and markers of bone metabolism in men representative of the astronaut population compared to exercise with placebo testosterone.

Current evidence suggests that the combination of testosterone and exercise will optimize the effectiveness of the existing exercise and nutritional countermeasures. Results from this proposal will lay the ground work for the implementation of combinational countermeasures that will additively work to maintain preflight physiology of astronauts during long-term spaceflight missions.

3.0 **Please attach a Cover Letter.**

Description

There are no items to display

4.0 **Is this a Student project?** ( ) Yes (•) **No**

5.0 **Is this an Engineering/Hardware evaluation?** (human in the loop) ( ) Yes (•) **No**

6.0 * **Please choose one of the following Scientific Investigations.**

- (•) New Proposal
- ( ) Legacy Protocol (Protocol approved prior to January 1, 2011)
- ( ) ISSMP/Complement/Data Sharing Plan
- ( ) Flight Analog Project Complement (Bedrest, NEEMO, DRATS, etc.)

7.0 * **Principal Investigator:**

Randall Urban

8.0 Co-Investigators:

Name
William Durham
Melinda Sheffield-Moore
Edgar Dillon
Douglas Paddon-Jones

9.0 Study Coordinator:

10.0 Key Personnel: (Key Personnel that are registered in the system.)

Name	Role
There are no items to display	

11.0 Unregistered Key Personnel: (Key Personnel not registered in the system. If you enter unregistered study staff they will not receive any notifications.)

	Name	Role
View	Kristofer Jennings	Statistician
View	Manisha Chandalia, MD	DSMP Safety Monitor

12.0 * **Are there letters of support?** (●) **Yes** ( ) No

13.0 Please attach letters of support?

ID
Jennings Letter of Support
Shandalia Letter of Support

ID: Pro0028

View: 1.1 CITI Training Documents

1.1 - CITI Training Documents

Principal Investigator: Randall Urban

Co-Investigators: William Durham
Melinda Sheffield-Moore
Edgar Dillon
Douglas Paddon-Jones

Study Coordinator:

Key Personnel: There are no items to display

Unregistered Key Personnel: Kristofer Jennings
Manisha Chandalia, MD

1.0 * **Please attach the most current CITI training documents for all US study team members listed above.**

	Name	CITI completion date
View	Edgar Dillon	5/8/2011
View	Kristofer Jennings	4/1/2011
View	Melinda Sheffield-Moore	10/8/2010
View	Manisha Chandalia	6/24/2010
View	Douglas Paddon-Jones	3/24/2011
View	William Durham	8/11/2010
View	Randall Urban	7/20/2009

2.0 **Please upload any Biosketch Documents:**

Description

There are no items to display

ID: Pro0028 View: 1.2 Conflict of Interest

1.2 - Conflict of Interest

1.0 *** Do any of the participating study investigators or other research personnel (or their immediate family/significant other) have a financial and/or intellectual property interest in the sponsor or products used with this project?**

○ Yes ◉ **No**

2.0 ***A Conflict of Interest Statement must be submitted with all human subjects initial and renewal protocols. The disclosure must be written on letterhead from the PI's institution.**

If YES, a signed letter on institutional letterhead must be submitted from the PI. The letter must answer affirmatively and disclose the relationship with the company or entity arrangements. A related financial interest does NOT automatically mean that the investigator cannot participate in the research. The CPHS will determine if the financial and or other conflict of interest can be reduced, eliminated or managed in order to allow participation in the research project.

If NO, a signed letter on institutional letterhead must be submitted from the PI. The letter must include the following statement:

“I do not receive any research support from non-public sponsors of research. I do not perform any validation research of a drug or device. I do not receive any gifts or income from individuals associated with my research studies. I do not use my position with _____________ (fill in the blank with name of employer, i.e. NASA, Wyle, USRA) or proprietary or confidential information obtained in performing my duties, in any marketing, investing, or commercial ventures.

Name	Version
Conflict of Interest Chandalia	0.01
Conflict of Interest Jennings	0.01
Urban Team Conflict of Interest Statements	0.01

ID: Pro0028

View: 1.3 Study Funding Information

1.3 - Study Funding Information

1.0 * **Has Funding been awarded for this study?**
Yes

2.0 **Study Name:**
Testosterone Supplementation as a Countermeasure against Musculoskeletal Losses during Space Exploration

* **Is this the funded study title?** ◯ Yes ◉ **No**

3.0 **If No, what is the funded study title?**
Testosterone and Leucine Supplementation as Gender Specific Countermeasures against Musculoskeletal Losses during Space Exploration

4.0 * **What is the source of funding for this study?**
NASA Research Announcement (NRA)

5.0 **If other please specify:**

6.0 **If there are multiple sources of funding for this study, please describe:**

7.0 **Attach input from your Division budget analyst, Experiment/Project/Grant Manager or letter, of support from the Division or Branch office chiefs documenting the availability of funds for this project.**

ID	Date Modified
NNX10AP86G	1/28/11

ID: Pro0028

View: 1.4 Scientific Merit Assessment

1.4 - Scientific Merit Assessment

1.0 * **Has this study received a scientific merit assessment?** (selected) **Yes** ( ) No

2.0 **If reviewed, what organization, review body and or individual conducted the review?**

	Organization	Reviewed Body	Reviewer
View	NASA	NASA NRA Review Panel	Dennis Grounds

3.0 * **Scientific Merit Assessment:** (check all that apply) Definition

name
Full Research and Development Protocol

4.0 **If Other**, please describe:

ID: Pro0028 View: 2.0 Background and Significance

2.0 Background and Significance

1.0 * **Background and Significance:**
Briefly sketch the scientific background leading to the present proposal.

Exposure to microgravity is known to result in a number of physiological impairments including loss of muscle mass and strength [1] loss of bone mass [2-5], reduced erythropoiesis [2, 6-8], reduced myogenic differentiation [9], and a functional decline in the hypothalamic-pituitary-gonadal axis (HPGA) and circulating testosterone concentrations [10]. Current countermeasures have been largely ineffective at preventing or reversing the physiologic decrements that occur in response to spaceflight. While in-flight exercise is currently the most effective and feasible countermeasure against musculoskeletal losses, both isokinetic and isotonic exercise have been shown to further reduce endogenous testosterone concentrations in a simulated spaceflight model (i.e. bed rest) [11]. As mission-length is continuously increasing and the recurring visions of NASA including human exploration of Mars, optimization of implemented countermeasures is of high importance. As stated in the 2008 Evidence Report Summary: Risk of Impaired Performance Due to Reduced Muscle Mass, Strength, and Endurance: *"Without doubt, transport between the Earth and Mars as well as the return trip represents the greatest risk to humans encountered in the history of human spaceflight."*

Our research team has extensive experience with the clinical use of androgens and amino acids in several populations including older and younger men and women. We have recent data on testosterone treatments in older men that extends 5 months (clinicaltrials.gov ID: NCT00957528) and are currently conducting an NCI funded clinical trial investigating the use of testosterone and amino acid treatments for 10 weeks in female cancer patients (NCT00878995). Further, our scientific and medical expertise at UTMB in conducting studies of muscle and bone metabolism make us uniquely qualified to undertake the proposed project. Finally, we are uniquely positioned to conduct the proposed research as our laboratory and study personnel are in close proximity to the NASA Flight Analogs Research Unit at UTMB.

2.0 **Please upload any documents supporting the above background and significance statement.**

ID	Date Modified
	There are no items to display

3.0 **Preliminary Studies:**
Preliminary data often aid reviewers in assessing how valuable the project is likely to be. If graphs or tables are used to convey information, please maintain a consistent style.

a Testosterone as a countermeasure in Astronauts

Testosterone promotes muscle protein synthesis. Testosterone increases muscle protein synthesis through its interaction with the androgen receptor and by enhancing muscle-specific IGF-I expression [12, 13]. Testosterone has also been

shown to increase satellite cell number and increase myogenic differentiation of pluripotent stem cells through the Wnt signaling pathway [14, 15]. Furthermore, testosterone has been shown to reduce systemic inflammatory cytokines such as TNFa, IL-6 and IL-1b[16-18], capable of mediating NF-κB dependent muscle proteolysis.

Rationale for maintaining physiological testosterone concentrations. The rationale for the use of testosterone as a countermeasure to maintain physiologic levels of testosterone is based on the following scientific evidence in men and women: 1) we have demonstrated that models of stress and trauma cause significant impairments in endogenous testosterone production and are associated with significant losses in muscle mass and strength [19]; and 2) simulated microgravity (i.e. stress) and spaceflight are both capable of disrupting the hypothalamic pituitary gonadal axis (HPGA), leading to reduced endogenous production of testosterone. There are reports of lowered testosterone concentrations in male astronauts [20], and animals [21-23] during real or simulated spaceflight. These observations during microgravity share striking similarities with the changes that take place during aging. Under terrestrial circumstances, the 46% drop in circulating testosterone noted in the astronauts studied by Strollo et al. would clinically classify them as hypogonadal [20]. Restoration or normalization of testosterone has been shown to ameliorate musculoskeletal losses in hind-limb suspended rats [24] and muscle mass but not strength in humans in the absence of exercise countermeasures [25]. However, no human studies have examined the therapeutic efficacy of testosterone in conjunction with an exercise protocol at preventing or reversing the many negative physiologic alterations that occur in response to bed rest or space flight.

Rationale for the exclusion of females. While testosterone is deemed safe for use in women in low doses, we instead propose to study the efficacy of a testosterone countermeasure in men in this proof-of-concept investigation. We contend that if the efficacy and safety of testosterone can be shown under these conditions in male representatives of the astronaut population, similar treatment paradigms can be tested in female representatives in future protocols.

Rationale for using testosterone in a cyclic dosing paradigm. For men with suppressed levels of endogenous testosterone, the standard of care treatment is an injection of intramuscular (IM) testosterone either weekly (100 mg IM) or every two weeks (200 mg IM). However, we have recent data demonstrating that a monthly cycled testosterone administration (i.e. dosed at 100 mg IM weekly on alternating months; one month on and one month off) is similarly anabolic and functional promoting to skeletal muscle as the continuous, standard of care approach, yet requiring only half the dose. Furthermore, we also have data showing that if we cycle testosterone weekly (100 mg IM weekly on alternating weeks; one week on and one week off), we can elicit a similar anabolic effect over 3-months, illustrating the potential versatility of alternative testosterone dosing regimens and the paradigm that similar positive anabolic results can be obtained with fewer doses of testosterone over time.

Our earlier research has shown that 6 months of continuous near-physiological testosterone administration increases LBM and muscle strength in healthy older men [26]. We have also shown that restoration of blood testosterone in severely burned male patients is anabolic to muscle protein, primarily by reducing muscle protein breakdown [27]. We recently published a study in which we administered a synthetic derivative of testosterone (oxandrolone, Oxandrin) to older women (7.5 mg po bid for 14 days) and measured mixed muscle fractional synthesis rate (FSR), anabolic markers androgen receptor and IGF-I protein expression at days 0, 5 and 14 [28]. These promising results from various populations prompted us to investigate alternative cyclic dosing regimens, further improving the safety and efficacy of androgens for clinical use.

We recently completed a 5 month placebo controlled double blind study in older men where we compared the standard of care continuous dosing regimen to that of a monthly cycled administration in which older men were provided weekly testosterone for one month, alternated by one month of placebo. Our data show that continuous (100 mg/wk) or monthly cycled (100 mg/wk for the 1st month, followed by one month placebo etc.) testosterone replacement increases muscle strength (**Figure 1**) and LBM (**Figure 2**) in older men with low normal endogenous testosterone concentrations, and reduces serum markers of bone resorption (**Figure 3**). The androgen stimulation of skeletal muscle protein synthesis was not reduced during the "off" cycles for testosterone. Furthermore, our data show that monthly cycled testosterone regimens (one month on, one month off) have similar positive effects on muscle mass as continuous testosterone regimens. Additionally, these results indicate that a monthly cycled regimen leads to similar reductions in markers of bone resorption, without the associated decrease in markers of bone formation that follows after months of continuous administration (**Figure 4**).

Here, we propose cycled testosterone replacement (two weeks of testosterone followed by two weeks off) offering a noninvasive, low-risk approach to prevent hormonal impairments that occur during long-term missions (i.e. 6 month Earth-Mars transit). A cycled testosterone approach offers several advantages over a continuous administration regimen: 1) The total amount of testosterone administered per person is greatly reduced, 2) It minimizes the potential side effects that may occur during continuous administration (elevated hematocrit, etc.), 3) It allows for a period of normalization for the HPGA during the "off' periods, and 4) It allows for repeated periods of increased protein anabolism during the "on" periods.

b. <u>Project impact</u>

Maintaining appropriate hormonal and nutritional balance during spaceflight is of critical importance in preventing musculoskeletal losses. It is our contention that the restoration of physiologic levels of testosterone is essential to prevent and/or restore spaceflight- and microgravity-induced losses in the musculoskeletal system. Current evidence suggests that the combination of testosterone, nutrition, and exercise will optimize the effectiveness of the existing exercise and nutritional countermeasures. Results from this proposal will lay the groundwork for the implementation of combinational countermeasures that will additively work to maintain preflight physiology of astronauts during long-term spaceflight missions.

c. Summary of Preliminary Data

1. Cycled testosterone treatment improves lean body mass (LBM), muscle strength, and bone metabolism.
2. Cycled testosterone treatment appears to improve bone metabolism compared with continuous administration, with lower testosterone exposure over time.

ID: Pro0028

View: 2.1 Medical Monitor and Level of Risk

2.1 Medical Monitor and Level of Risk

1.0 * **What is this study's Medical Monitor level?** Definition

Level 3

Level 4

2.0 **If different parts of the study have different levels of risk, please outline the risks associated with each part.**

Level 3: Muscle Biopsy

Level 4: MRI, Ultrasound

3.0 * **What is this study's level of risk?** Definition

( ) Minimal Risk

(•) Greater than Minimal Risk

ID: Pro0028

View: 2.2 Drugs, Devices and Biologics

2.2 - Drugs, Devices and Biologics

1.0 * **Does this study include any drugs and/or biologics under investigation?** ○ Yes ◉ **No**

If yes, please decribe the Investigational New Drug (IND) status for the drugs and/or biologic under investigation in this study.

2.0 * **Does this study include any devices under investigation?** ○ Yes ◉ **No**

If yes, please decribe the Investigational Device Exemption (IDE) status for Devices under investigation in this study.

ID: Pro0028 View: 2.3 Required Reviews

2.3 - Required Reviews

1.0 * **Requested Review Type:**

	Name
○	Exempt
○	Expedited
●	Full IRB Review

2.0 **Please select all applicable Ancillary Reviews.**

Name

There are no items to display

ID: Pro0028

View: 2.4 Study Summary

2.4 Study Summary

1.0 * **Expected Start Date:**
3/2/2011

2.0 * **Expected End Date:**
8/29/2013

3.0 * **Type of Subjects:**

Name
Non-Astronaut

4.0 * **Type of Study:**

ID
Ground-based

ID: Pro0028 View: 2.5 Study Locations

2.5 - Study Locations

Check all locations where data collection will be obtained

1.0 * **Study Locations:**

ID	Name	Category
Other	Other	Other

ID: Pro0028

View: 2.6 Other Sites/Institutions

2.6 - Other Research Sites

Study Locations:

ID	Name	Company Category
Other	Other	Other

1.0 * **If any part of this study will be performed at a site not associated with NASA. Where will this research be conducted? Attach all appropriate documentation:**

Name	IRB Approval Status	Description
The University of Texas Medical Branch	Approved	

ID: Pro0028 View: 3.0 Specific Aim and Hypothesis

3.0 - Specific Aim and Hypothesis:
(Examples: Engineering, Human Research Project**)**

1.0 ***Specific Aims:** For each Specific Aim please add an hypothesis, primary outcome, and statistical analysis.

Aim 1: To determine the effect of cycled testosterone replacement in conjunction with resistance exercise during bed rest on **muscle mass, muscle strength, and fatigue** in **men** aged 24-55 years.

Aim 2: To determine the effect of cycled testosterone replacement in conjunction with resistance exercise during bed rest on **markers of bone metabolism and bone mass** in **men** aged 24-55 years.

2.0 ***Hypothesis(es):** List hypothesis(es) and an associated primary outcome measure(s) for each specific aim. If the study is not designated to test a hypothesis, describe the goals of the project as related to this specific aim. (ex: "determine hardware/logistic feasibility," "estimate mean and standard deviation of intervention effects," "evaluate degree of association between X and Y")

Aim 1: Cycled testosterone replacement (weekly testosterone injections for 2 weeks, followed by 2 weeks off, etc.) in conjunction with exercise will have an additive effect in preventing loss of muscle mass and muscle strength in *men representative of the astronaut population* compared to exercise with placebo testosterone.

Aim 2: Cycled testosterone replacement (weekly testosterone injections for 2 weeks, followed by 2 weeks off, etc.) in conjunction with exercise will have an additive effect in preventing loss of bone mass and alterations in markers of bone metabolism in *men representative of the astronaut population* compared to exercise with placebo testosterone.

3.0 * **Primary Outcome Measure:** (Dependent Variables)

Aim 1: Muscle volume, Lean body mass, Fat mass, Muscle performance, Muscle fatigue.

Aim 2: Serum and Urinary markers of bone metabolism, Bone mineral density, NASA Flight Analogs Project Standard Measures.

4.0 ***Statistical Analysis:** Breifly describe what statistical analysis(es) will be applied in order to address the hypothesis/primary outcome.

Note: If the above hypothesis/primary outcome is quantitative, briefly descibe how qualitative data will be analyzed.

Aim 1: Paired t-test (or nonparametric alternative, Wilcoxon signed rank test), ANCOVA, General Linear Mixed Model (GLMM).

Aim 2: Paired t-test (or nonparametric alternative, Wilcoxon signed rank test), ANCOVA, General Linear Mixed Model (GLMM).

ID: Pro0028 View: 3.1 Study Design

3.1 - Study Design Characteristics

1.0 * **Study Design:**

**Design Characteristics – treatment / intervention categories, other experimental variables (eg: time in bedrest) – total number of subjects**
Nested - separate subjects in each treatment category

**Randomized – intervention with 2+ groups – subjects are randomly assigned to a specific treatment / condition**
Double Blinded - subject and investigators do not know which condition subject is in

2.0 **Sample Size:**
Indicate how many subjects will be studied and why this number was chosen.

- Power/Sample size calculations should be included for hypothesis-testing studies (may require assistance by a statistician).
- Alternatively, if the purpose of the experiment is to estimate a characteristic of the response measure(s) (e.g. mean, SD of change, etc) then provide evidence that the proposed sample size will enable estimation within a reasonable degree of error.
- Novel studies (first-time-ever) and descriptive/feasibility studies benefit from an understanding of the relationship between sample size and precision (may require assistance by a statistician).

Bed rest Control: 8 subjects

Bed rest + Exercise: 8 subjects

Bed rest + Exercise + Testosterone: 8 subjects

A power analysis was conducted to demonstrate that our sample size is sufficient to detect statistical differences and avoid type II errors. The sample sizes are based on the primary measures of metabolic and functional outcomes. We use conservative estimates of the variation where possible.

Primary metabolic measure: lean body mass: We recently demonstrated that a group of subjects on testosterone had increased LBM of 4.2 kg compared to controls that had a loss of 2 kg. The SDs were 1.6 and 2.2 [26]. A sample size of 8 in each group will have 96% power to detect a difference in means of 4 assuming that the common standard deviation is 2 using a two group t-test with a 0.050 two-sided significance level. If the SD=2.5, there is still 84% power.

*Primary metabolic measure: lean leg mass:*Preliminary estimates for lean leg mass in males comes from Ferrando et al. in which treated subjects had increases of mean±SD of 4.2±1.6 and controls on average had losses of -2.0±2.2 [26]. A sample size of 8 in each group will have 84% power to detect a difference in means of 4 assuming that the common SD is 2.5 using a two group t-test with a 0.05 two-sided significance level.

Primary functional measures: We recently demonstrated that a group of subjects on testosterone had increased leg extension, triceps extension, and bicep curl strength compared to controls who had no gain or losses [26]. Estimates of variation come

from this work. A sample size of 8 per group will have 80% power to detect a difference in means of 15 kg between the groups for leg extension when the SD=10 using a two group t-test with a 0.05 two-sided significance level. With 8 per group there is 92% power for tricep extension to detect a difference of 10 kg (SD=5.5) and 98% power to detect a difference of 9 kg (SD=4) for bicep curl.

*Summary of power for primary outcomes:*We have demonstrated that with N=24 (N=8 for each of the treatment groups) should provide sufficient power to assess the specific aims and hypotheses for our study using calculations based on a two-group t-test. The power presented here is minimal as it does not take into consideration the repeated measures design which will benefit the power aspect of the study. In other words by using the proposed repeated measures mixed model, the power characteristics will be improved as the time points gather strength across time.

ID: Pro0028 View: 3.2 Explain Study design

3.2 - Explain Study Design

You indicated the following design characteristic:

1.0 **Study design characteristics selection:**

**Design Characteristics – treatment / intervention categories, other experimental variables (eg: time in bedrest) – total number of subjects**
Nested - separate subjects in each treatment category

* **Study design: Explain all selections listed above.**

Male volunteers (24-55 years) will be randomly assigned to one of 3 bed rest groups (non-exercising control + placebo, exercise + placebo, exercise + testosterone) using a standard of care approach with NASA spaceflight nutritional requirements and standard exercise protocols as the control condition. An example of a 70-day bed rest timeline is provided in **Figure 5.**

non-exerciseing control + placebo (n=8)

exercise + placebo (n=8)

exercise + testosterone (n=8)

Our study design is compatible with a head down tilt (HDT) bed rest protocol. It is important that the proposed testosterone countermeasure is tested under conditions that can be compared to other countermeasures.

Our general hypothesis is that the maintenance of normal physiologic male levels of testosterone during spaceflight will protect against the functional loss of muscle and bone, and will maximize the efficacy of existing resistance exercise protocols at preventing or reversing functional impairments that occur during bed rest.

To achieve these goals we will test the following **specific hypothesis** before, during and after 70 days of bed rest:
Cycled testosterone replacement (weekly testosterone injections for 2 weeks, followed by 2 weeks off, etc.) in conjunction with exercise will have an additive effect in preventing loss of muscle mass, muscle strength, bone mass, and markers of bone metabolism in *men representative of the astronaut population* compared to exercise with placebo testosterone.

Specific Aim 1A: To determine the effect of cycled testosterone replacement in conjunction with resistance exercise during bed rest on **muscle mass, muscle strength, and fatigue** in **men** aged 24-55 years.

Specific Aim 1B: To determine the effect of cycled testosterone replacement in conjunction with resistance exercise during bed rest on **markers of bone metabolism and bone mass** in **men** aged 24-55 years.

***Testosterone replacement countermeasure**:* During the respective treatment periods, weekly placebo or testosterone enanthate injections (100 mg/wk) will be administered.When the nadir serum total testosterone concentration exceeds the normal range, we will reduce the next intramuscular injection from 100 mg to 75 mg and continue to monitor the nadir serum total testosterone concentration. If the nadir value persists above the normal range, we will reduce and additional 25 mg to 50 mg weekly.

Exercise countermeasure:Our protocol is compatible with and will utilize the Integrated Resistance and Aerobic Training (iRAT) protocol that is planned for testing in bed rest.

Nutrition: Our protocol is compatible with, and will utilize, the standard FAP dietary guidelines.

Blood Collection.We will collect morning fasting blood before iRAT, before bed rest, weekly during bed rest and after bed rest (12mL each) for measurements of hormones (i.e. Testosterone, Estradiol), markers of bone metabolism (i.e. NTX, Osteocalcin, BAP, sRANKL), plasma amino acids, inflammatory cytokines (Millipore Human Cytokine-13plex kit, Millipore, Billerica, MA), and other markers of protein catabolism including serum activin A and myostatin. Maximum blood required over the entire bed rest duration will be (13 x 12 mL) = 156 mL (see Blood Volume Request for requirement by test).

Urine Collection. Twenty-four hour (void by void) urine collections will be collected every week for measurements of hormones (Cortisol) and markers of bone degradation (Pyrilinks-D®).

Saliva Collection. Saliva will be collected every week for measurements of hormones (Testosterone, Cortisol) and other markers of protein catabolism.

Muscle Biopsies. We will collect muscle tissue from the *vastus lateralis* before, during, and after bed rest for the determination of proteins and RNA that regulate muscle protein synthesis and breakdown (mTOR, S6K1, Akt, AMPK, 4E-BP1, eEF2, eIF 4G, Follistatin, TCF-4, Smad7, MyoD, Myosin HC2, Androgen Receptor, B-Catenin, Myogeni).

Mood/Fatigue. Daily (MD Anderson Brief Fatigue Inventory and after exercise the 10-Point Scale for Fatigue) and weekly (Profile of Moods States & Multidimensional Fatigue Symptom Inventory -Short Form) data will be collected by questionnaires to determine changes in perceived mood or fatigue. These
are validated questionnaires and were selected based on prior experience.

Collected weekly (post-exercise, before lights-out):

Profile of Mood States (POMS). Subjects will indicate their current mood by checking off a series of words on a 5 point scale (0= Not at all, 5= Extremely).
Multidimensional Fatigue Symptom Inventory-Short Form (MFSI-SF). Subjects will indicate their feelings over the past 7-days on a 4 point scale (0= Not at all, 4= Extremely).

Collected daily (post exercise, before lights-out):
MD Anderson Brief Fatigue Inventory (BFI). Subjects will rate their fatigue on a 10-point scale (0= no fatigue, 10= as bad as you can imagine). Questions address fatigue; right now; average during past 24-hrs; worst during past 24 hrs; and level of interference with activities.

Collected 3-Times daily (on exercise days only):

10-Point Scale for Fatigue (10PSF). Identical to question #1 on the BFI. Subjects will indicate their fatigue on a 10 point scale (0= No fatigue, 10= Worst fatigue) before, immediately following, and 4 hours after completing the exercise regimen. (control subjects will be day/time matched to exercisers). This test may be completed orally, in which case staff will record the answer on the sheet.

Hardcopy questionnaires will be provided for subjects to fill out and return to FARU staff.

In addition, we have consulted with Dr. Kim Seeton and we will include the Buss-Durkee Hostility Index (BDHI) as an assessment of hostility as recommended. This will be administered weekly in conjunction with the POMS above.

Restoration of testosterone concentrations in hypogonadal men often results in improved mood (i.e. decreased depression, improved cognition) and decreased fatigue [29, 30]. Therefore, the proposed low-dose testosterone treatment regimen is not expected to result in the negative mood changes often associated with high-dose misuse of anabolic steroids (i.e. irritability, aggression)[31]. Mood disorders are not prevalent among **young** males with low doses of chronic abuse (less than 300mg/week) [46]. The dose of testosterone proposed here (intermittent administration of 100mg/week) is well below this dose and **an order of magnitude below** the high doses where mood disorders are more likely to present (i.e. more than 1000mg per week) [46,47]. While the effects of testosterone replacement on mood changes during bed rest are yet unknown, no additional behavioral or psychological issues are anticipated to those normally encountered during long term bed rest [32]. If anything, we expect that testosterone treatment will result in improved mood and reduced fatigue.

ID: Pro0028 View: 3.3 Randomized Study

3.3 - Randomized Study

Describe your randomization plan, including use of strata (if any), and method for randomizing study recruits.
(May require assistance from statistician)

1.0 Study design characteristics selection:

Randomized – intervention with 2+ groups – subjects are randomly assigned to a specific treatment / condition

Double Blinded - subject and investigators do not know which condition subject is in

* **Randomized Study:**

Male subjects will be randomized to one of three double-blinded groups (non-exercising control + placebo, exercise + placebo, exercise + testosterone). The pharmacy and safety monitor will have access to the coding key. The subjects, bedside nurses, PI and Co-Is will remain blinded throughout data collection and analyses. The attending physician, safety monitor, study lead, study management, and data manager may remain unblinded.

ID: Pro0028 View: 4.0 Procedures

4.0 - Study Procedures, Tests, Evaluation

.

1.0 * **Study Procedures, Tests, Evaluations:** Please list, in sequence, all study procedures, tests, and evaluations required for the study.
In order to evaluated the efficacy of the proposed countermeasure this protocol will share measures from FTT, iRATS, and Standard Measures.

The **primary** dependent measures from this research will include muscle cross-sectional area and volume (determined by Magnetic Resonance Imaging (MRI)), lean body mass (LBM determined by intelligent Dual-energy X-ray Absorptiometry (iDXA)), fat mass (FM determined by iDXA), muscle performance measures (Biodex). The **secondary** dependent measures will include bone mineral density (BMD determined by iDXA), serum and urinary markers of bone turnover, markers of skeletal muscle signaling and inflammation, muscle fatigue, and quality of life.

***Bone Mineral Density (BMD) using UTMB's new iDXA*.** Whole body iDXA scans will be obtained prior to bed rest, monthly during bed rest, and one month after bed rest. We will specifically measure LBM (total, trunk, leg), BMD (total, hip, spine), and FM (total, trunk, leg, %). UTMB's new iDXA is the latest state-of-the-art DXA that has the capability to measure muscle and bone with a much greater degree of sensitivity.
Additionally, the new iDXA has the capability to measure additional parameters of fracture risk not obtained with the standard DXA*.*

MRI. To determine muscle cross-sectional area and total volume, subjects will have their lower body imaged in a clinical 1.5 Tesla magnet (GE Signa, Milwaukee, WI) located in the UTMB Department of Radiology. Briefly, image slices will be taken from the greater trochanter to the ankle joint with the muscle at rest. Image data files generated will be analyzed for appendicular total and muscle volumes using NIH Image software (NIH Image public domain software). Muscle volume (cm^3) will be computed as the addition of individual slice areas multiplied by slice thickness (5 mm). The full procedure including details of the anatomical positioning and MRI imaging techniques have been described previously (16).

Mood, Fatigue, and Quality of Life Questionnaires. Before, during and after bed rest we will measure mood and quality of life using validated questionnaires on a daily or weekly basis. Psychological state will be assessed *weekly* using the 65-item Profile of Mood States (POMS) questionnaire, a validated measure that yields an overall score (range 0-168, with higher scores indicating more disturbance in mood) and 6 subscales (tension-anxiety, depression-dejection, anger-hostility, vigor-activity, fatigue-inertia, and confusion-bewilderment). To assess fatigue, the Multidimensional Fatigue Symptom Inventory (MFSI) [33] and the MD Anderson Brief Fatigue Inventory (BFI) [34], will be completed *every day*. In addition, subjects will indicate their fatigue on a 10 point scale for fatigue (10PSF) (0= no fatigue, 10= as bad as you can imagine) before, immediately following, and 4 hours after completing the exercise regimen on *exercise days only*.

Muscle Biopsies.A total of three muscle biopsies (~100-200 mg) will be taken from the *vastus lateralis*, 10-15 cm above the knee [35, 36]. One muscle biopsy will be taken prior to the start of bed rest, one during bed rest following the second cycle of testosterone and one at bed rest completion. Muscle biopsies will be taken using strict aseptic procedures from the lateral portion of the *vastus lateralis* muscle. This is a commonly performed procedure in our research studies [28, 36-38] (see Part V: Risks and Benefits). All tissue will be snap frozen in liquid nitrogen to stop all enzymatic reactions and frozen at -80˚C for later analysis of signaling, inflammatory markers, and catabolic markers such as those in the ActRIIB signaling pathway.

Skeletal Muscle Signaling. Muscle tissue from the biopsies will be used to assess cell signaling proteins. In the men we will examine key signaling proteins downstream to Testosterone: Follistatin, TCF4, Smad2/3, Smad7**,** MyoD,Myosin Heavy Chain 2 (MHC2), Androgen Receptor (AR), beta-Catenin, Myogenin, myostatin and other protein involved in the ActRIIB signaling pathway**.** A detailed description of the SDS PAGE (sodium dodecyl sulfate polyacrylamide gel electrophoresis) and

immunoblotting techniques is available in recent publications [39-41].

Cardiac Compliance. Diastolic Function, measured noninvasively via transthoracic echocardiography, will be determined from the mitral inflow velocity (E-wave) and relaxation of the mitral annulus (e') before, during, and after bed rest. The ratio (or index) of E-wave/e' is independent of preload and can be used to assess cardiac compliance (ventricular stiffness). Normal diastolic function is represented by an E/e' < 8. An E/e' index of 8-12 suggests reduced compliance and and E/e' index > 12 suggests severe restriction. It is expected that inactivity results in increased ventricular stiffness. This measure is included to determine whether testosterone replacement in conjunction with exercise offers protection against possible bed rest induced changes in ventricular stiffness.

Oral Glucose Tolerance Test (OGTT). Glucose tolerance will be determine before, during, and after bed rest using a standard OGTT and a breath test. After an overnight fast (water allowed), an antecubital venous IV is placed for the collection of blood samples. During the baseline blood collection, a simultaneous breath sample is collected by having the subjects breathe into a breath collection bag fitted with one way valves. Following the baseline sample collection, a drink containing 75 g glucose and 150 mg U-$^{13}C_6$-glucose is administered and consumed within 1 minute. From this point (t=0 minutes) blood and breath samples are collected at 30 min, 1hr, 2hrs, and 3hrs. For the collection of breath samples, the subjects are instructed to breath normally, hold their breath for 3 seconds, and exhale completely into the provided collection bags. Subjects remain at rest throughout the OGTT. The ratios of $^{13}CO2$ to $^{12}CO2$ in single breath samples are measured using a UBiT-IR300 infrared spectrophotometer (Otsuka Electronics Co., Ltd, Hirakata, Osaka, CV ≤ 1.0 %). All results are calculated as per mille (‰) change of $^{13}CO2$ abundance from the baseline breath sample and expressed as ‰ delta over baseline (‰DOB). Insulin is an important regulator of skeletal muscle metabolism including glucose disposal as well as protein synthesis. It is anticipated that inactivity results in reduced glucose tolerance. This measure is included to determine whether testosterone replacement in conjunction with exercise offers protection against possible bed rest induced changes in glucose tolerance.

Muscle Function and Fatigue Testing. Before, during, and after bed rest the subject will perform muscle function testing on the Biodex dynamometer. Muscle function testing will include tests of maximal voluntary contraction (MVC), which involves producing the maximal amount of force one time, *as well as skeletal muscle fatigue*, which involves measuring the decline of contractile performance during repetitive contractions. These tests will be conducted using leg extension movements on a Biodex dynamometer. An integrated functional testing protocol has been developed in conjunction with Dr. Lori Ploutz-Snyder (see details of Dr. Ploutz-Snyder's functional testing protocol).

Vitamin D supplementation

Any overt metabolic or vitamin deficiency during a focused investigation involving intervention therapies should be regarded as a confounding factor. Of highlighted importance in this study is the possible presence of Vitamin D deficiency prior to enrollment because of its known importance in optimum bone health. While vitamin D deficiency is likely to affect muscle metabolism, little is known regarding the direct effects of vitamin D on skeletal muscle and about the presence of Vitamin D receptors in skeletal muscle [49]. However, the male reproductive tract is a direct target for vitamin D and while some have found associations between androgen concentrations and 25(OH)D concentrations in men [50] others have not [51]. Vitamin D supplementation (3,332 IU daily for 1 year) has recently been shown to benefit testosterone concentrations in overweight men (aged 20-49 years) with both vitamin D deficiency and low normal testosterone [52]. While significant and of high interest both clinically and scientifically, it should be noted that these increases were measured over a year and that, while vitamin D status improved markedly, testosterone concentration improved modestly (10.7 ± 3.9 nmol/l to 13.4 ± 4.7 nmol/l), staying well below the mid normal ranges (9.09-55.28 nmol/l) in these subjects . In order to minimize vitamin D status as a confounding factor and to ensure optimum health of the subjects before bed rest we will correct for vitamin D deficiencies in subjects prior to the onset of the study according to standard FAP procedures outlined in the exclusion criteria.

In addition we will utilize the following NASA Standard Measures:

Clinical Nutritional Assessment, Clinical Laboratory Assessment, Muscle Function Testing, Aerobic Capacity – Cycle Ergometry, Functional Fitness.

Please attach any documents supporting the statements in question 1.0. This may include tables, diagrams, or pictures.

Description	ID	Date Modified
Figures	Figures	2/25/11
Blood Volume Request	Blood Volume Request	1/28/11
Statistical Analysis Supplement	Statistical Analysis Supplement	1/28/11

2.0 * **Will any interviews, questionnaires, or surveys be conducted for the study?** (•) **Yes** ( ) No

If yes, Please attach all interviews, questionnaires, and surveys

Title	ID	Date Modified
	MD Anderson Brief Fatigue Inventory -- BFI	1/13/11
	10-Point Scale of Fatigue -- 10PSF	1/13/11
	Profile of Mood States - POMS	1/13/11
	Multidimensional Fatigue Symptom Inventory -- MFSI-SF	1/13/11

3.0 * **Will subjects or their health care provider be given the results of any experimental tests that are performed for the study?** (•) **Yes** ( ) No

If "Yes," please describe the tests, provide a rationale for providing subjects with the experimental test results and explain what, how and by whom subjects and their health care provider will be told about the meaning, reliability, and applicability of the test results for health care decisions.

Research results are available upon request by the subject.

Clinical data collected for safety monitoring will be included in the medical records.

Research data will not be included in the medical records.

4.0 * **Will subjects undergo any study procedures or tests off-site?** (•) **Yes** ( ) No

ID: Pro0028 View: 4.1 Procedures page 2

4.1 - Procedures part 2

1.0 * **Time commitment:** Indicate how much time will be required of the subjects, both per visit and in total, for the study.
Anticipated time commitment:
Standard FAP screening timelines will be used.
Pre-Bed Rest: 13 days at FARU
Bed rest: 70 days at the FARU
Post bed rest: 14 days at FARU

2.0 * **Facilities:** List the locations where study procedures will be performed. Please provide a description of the facility if appropriate.

a. NASA Flight Analog Research Unit (NASA-FARU).

This unit is housed in 7,100 sq. ft. of the 6th Floor of the Children's Hospital. The facility is a national center for collaborative, ground-based, health oriented, human spaceflight research. The unit is a satellite of the Institute for Translational Sciences – Clinical Research Center (ITS-CRC), administered and staffed by CRC personnel. Funding is entirely from NASA as a pass-through on the ITS-CRC grant from the NIH/NCRR. The facility will play an increasingly important role with the approaching end of the Shuttle Program.

b. iDXA

The high-resolution GE Lunar iDXA™ densitometer is located on the 10th floor of the John Sealy Hospital. This instrument is property of the Sealy Center on Aging and is available to ITS-CRC investigators at no cost.

a. Division of Endocrinology Muscle Metabolism Laboratory.

(Director: M. Sheffield-Moore, Ph.D.). The main focus of the muscle metabolism laboratory is to determine effective treatments for muscle loss in several different populations, including cancer and aging. Current studies include how anabolic treatments such as testosterone and/or amino acid supplementation influence muscle metabolism in cancer patients. Additionally, the lab studies blood flow and muscle perfusion as it relates to aging. This laboratory (1900 sq ft space) is located in the John Sealy Annex, which is adjacent to the John Sealy Towers. The lab is equipped with (one) Immulite 2000 for high throughput, continuous random access hormone and cytokine immunoassay analyzer; (two) Agilent Technologies 7890A GC Systems, 5975C inert XL MSD Turbo EI Mainframes with autosamplers; (two) Thermo Savant speed-vacuum systems for sample concentration and desiccation; (one) Eppendorf microcentrifuge; (one) Eppendorf bench centrifuge to separate blood subfractions; (one) BioRad MyiQ Real Time PCR machine to determine gene expression; (one) BioRad ChemiDoc Imager for imaging western blots and DNA/RNA gels (three) BioRad electrophoresis systems with blotting apparatus; (one) MTX Lab Systems Fluorometer; (one) Beckman Coulter DU730 UV/Vis Spectrophotometer; (one) Biotek ELx800 plate reader; (one) Meretek UBiT-IR 300 for breath analysis; (one) Biosafety sterile cabinet for tracer preparation; (one) fume hood; (two) -80˚C freezers; (two) -20˚C freezers; (two) 4˚C refrigerators; (one) sonicator; (three) homogenizers and a number of smaller standard laboratory items.

3.0 * **Adequacy of Resources:** Principal investigators must have the necessary resources required to conduct the proposed research in a way that assumes the rights and welfare of participants are adequately protected. Describe the resources you have in place to conduct this study. Examples may

include personnel, funding, and equipment required to perform the study.

All equipment and personnel to support this study are available at UTMB or JSC. There is sufficient staff to conduct the study. The FARU and ITS-CRC are in place to support bed rest studies.

4.0 * **Please provide/describe the test termination criteria guidelines and associated rationale.**

Standard FAP criteria and exclusion criteria.

ID: Pro0028

View: 5.0 Human Test Subject Facility

5.0 - Human Test Subject Facility

1.0 * **Will all subject recruitment be handled by the Human Test Subject Facility (HTSF) located at Johnson Space Center?** (selected) **Yes** ( ) No

ID: Pro0028 View: 5.2 Remuneration

5.2 - Remuneration

Payment to subjects

1.0 * **Will subjects receive remuneration for study participation?**
YES

2.0 **Please describe the remuneration the subject will receive.**

According to standard FAP bed rest procedures.

ID: Pro0028

View: 5.3 Cost Related to Participation

5.3 - Costs Related to Participation

1.0 * **Select all categories indicating costs which participants or their insurance companies will be responsible for:**

	Name
[x]	Participants will have no costs associated with this study
[ ]	Study Related Procedures
[ ]	Study Drugs or Devices
[ ]	Other

2.0 **If study participants or insurance companies will assume costs for this study, describe the procedures, drugs, or devices for which the participants must assume costs:**

ID: Pro0028

View: 5.4 Study Population

5.4 Study Population

1.0 * **Check all that apply to describe your study population:**

Study Population
Normal Healthy Volunteers (Non-Astronaut)

2.0 * **General description of study population:**

Healthy males aged 24-55 years.

3.0 * **Indicate the inclusion and exclusion criteria for enrollment:**

In addition to the standard FAP criteria we will follow the following exclusion criteria:

i. Exclusionary medications will be an anticoagulant (Coumadin) because of the risk of bleeding during the biopsy procedure. Additional medications which will be disallowed for participation include: anabolic steroids, nitrates, antihistamines, andglucocorticoids.
ii. The subjects must have a minimal VO_2max score of 30 ml/kg and minimal isokinetic knee extension strength/body weight ratio of 2.0 Nm/kg.
iii. Subjects with LDL cholesterol above 200 mg/dL will be excluded because testosterone administration may elevate LDL cholesterol levels further.
iv. Any man with a history of breast cancer or prostate cancer, or any indication of an occult carcinoma from an elevation of prostate specific antigen (PSA) above 3.0 ng/ml during screening by HTSF, or severe benign prostatic hypertrophy (BPH) by history (frequent urination, reduced stream) will be excluded. In this study we will give parenteral testosterone to young men in a cyclic fashion. We will check a screening PSA in these men and include men only with a PSA < 3.0 ng/ml. This is based on a recent study which showed that serum PSA levels of 4 or greater occurred at 2.1% in subjects in their 30's and 1.6% in subjects in their 40's [42]. The use of the PSA as a screen for prostate cancer is controversial. The American College of Preventive Medicine concluded in 2008 that there is insufficient evidence to recommend routine screening for prostate cancer with a PSA [43]. This position has not changed as recently reviewed although risk stratification of PSA-based screening is being considered [44]. Parenteral testosterone administration in men with erectile dysfunction increased PSA levels from baseline 37% but did not result in a doubling of the PSA level which is a standard measurement of disease progression in men being treated for prostate cancer [45]. Therefore, in our study we will check baseline PSA levels and monitor them monthly in our subjects receiving testosterone. PSA velocities greater than 0.75 ng x ml^{-1} x yr^{-1} while receiving testosterone will result in withdrawing the subject from the study and referral to an urologist.
v. Subjects with liver dysfunction evidenced by a history of hepatitis B or hepatitis C, or by a three-fold elevation of liver enzymes (Alk phos, ALT, AST) above normal on screening will be excluded from the study. Testosterone can have hepatotoxic effects in some subjects and should be used with careful monitoring of LFTs.
vi. Testosterone and other anabolic steroids can cause fluid retention that could worsen uncontrolled hypertension. Any subject with a blood pressure on three consecutive measurements taken at one week intervals that has a systolic pressure > 140 or a diastolic blood pressure > 90 will be excluded. This is the definition of hypertension as established by the Joint National Committee on Detection and Evaluation of High Blood Pressure. Subjects will be included if they are on two or less blood pressure medications and have a blood pressure below these criteria.
vii. Any subject who has a major medical illness such as diabetes, chronic obstructive pulmonary disease, or sleep apnea will be excluded. Moreover, subjects will not have a recent history of smoking tobacco. Morbidly obese older men (BMI > 35) will also be excluded.
viii. Subjects with evidence of kidney disease (serum creatinine > 2.0mg/dl) will be excluded from participation.
ix. Any subject with thyroid disease as determined by an abnormal TSH level will be excluded from participation.
x. Any subject testing positive for HIV will be excluded as the effects of this disease on the

inflammatory process are unknown.
xi. Allergy to iodine, a component of Betadine which is used to prepare the subject's skin for invasive procedures, will be cause for exclusion from this study.
xii. Subjects who engage in high intensity resistance training on a regular basis will be excluded. Many studies show that exercise, particularly resistance exercise increases muscle strength and muscle protein synthesis.
xiii. Subjects with a known coagulation disorder or with clinical evidence indicative of a bleeding disorder (easy bruising, "free bleeders") will not be enrolled in this study due to potential problems that could arise from muscle biopsy procedures.
xiv. Subjects with agitation/aggression disorder will be excluded.
xv. Subjects with smoking history of >20 pack per year or unable to abstain from smoking for duration of the study will be excluded.
xvi. Subjects with inplanted electronic devices (e.e pacemakers, electronic infusion pumps, stimulators) will be excluded.
xvii. Subjects with recent (6 months) treated cancer other than basal cell carcinoma will be excluded.
xviii. Subjects with a recent history of GI bleed (<12 months) will be excluded.
xix. Subjects with hemoglobin or hematocrit lower than acceptable lab values will be excluded.
xx. Subjects with a history of stroke with motor disability will be excluded.
xxi. Subjects currently on a weight-loss diet will be excluded.
xxii. Subjects with any other condition or event deemed exclusionary by the PI and physician will be excluded.
xxiii. Subjects will be screened with serum 25-hydroxyvitamin D level using 30-day lab values.
-If the 30-day value is <50 nmoles/L, the subject will be recommended to take 2000 IU/day of Vitamin D3 until admittance into the study at UTMB. Once admitted into the study, the subject will receive 2000 IU/day of Vitamin D3 during the pre-bed rest phase at UTMB.
-If the 30-day value is >50nmoles/L, the subject will not need to take the Vitamin D3 before admittance into the study at UTMB. The subject will also not need to receive the Vitamin D3 during the pre-bed rest phase of the study at UTMB.
-All subjects will receive 800 IU/day of Vitamin D3 during the in-bed rest phase of the study at UTMB.
xxiv. Subject with HDL above or below the normal clinical ranges used by HTSF during screening will be excluded as this may be an indication of steroid use.
xxv. Subjects with testosterone above or below the normal clinical ranges used by HTSF during screening will be excluded.

4.0 *** If there are any age, ethnic, language, or gender-based exclusion criteria, please provide justification.**

We will enroll only men in this protocol. If the efficacy and safety of testosterone in mitigating the musculoskeletal losses can be shown in men during these bed rest conditions then further considerations can be taken to include females in future protocols.

5.0 **Are pregnant subjects excluded from participation?**

(•) **Yes** ( ) No

If yes, use the following statement

"Female subjects in this study will model those in the astronaut population for whom participation in space missions is not allowed during pregnancy; therefore, for female subjects, a non-positive result from a pregnancy test will be required prior to inclusion in the study and prior to each experimental session."

ID: Pro0028 View: 5.41 Normal Healthy Volunteers

5.41 - Normal Healthy Volunteers (Non-Astronauts)

1.0 Target Number of Participants:
24

2.0 Please describe specific criteria for this population.

We anticipate having to recruit 32 subjects to reach our target number of 24 participants. We will utilize the inclusion and exclusion criteria described above.

ID: Pro0028 View: 5.5 Study Population Summary

5.5 - Study Population Summary

1.0 Number of Participants by Group

Normal Healthy Volunteers (Non-Astronaut)	24
Astronauts	0
Other	

2.0 What is the maximum number of subjects you plan to recruit for this site? (Integer values only)

24

3.0 If this is a multi-site study, indicate the projected total subject accrual. (Integer values only)

ID: Pro0028 View: 5.6 Risk Assessment

5.6 - Risk Assessment

1.0 * **Risks associated with this study:**

	Risk discomfort	Minimize risks
View	Testosterone: Androgen therapy in humans has been associated with increased fluid retention, an increase in acne and sex drive, changes in the blood cholesterol profile (higher total cholesterol and LDL cholesterol, lower HDL cholesterol), changes in hematocrit (increased red blood cell count), damage to the liver, sleep apnea, and the possibility that testosterone can be converted by the body into estrogen, leading to breasts enlargement and soreness. Medications like this one have been associated with potential prostate enlargement and with promoting the growth of cancer cells. Male subjects will be checked for this periodically during the study by means of a blood test called a PSA (Prostate- Specific Antigen) level.<br><br>Restoration of testosterone concentrations in hypogonadal men often results in improved mood (i.e. decreased depression, improved cognition) and decreased fatigue [29, 30]. The proposed low-dose testosterone treatment regimen is not expected to result in the negative mood changes often associated with high-dose misuse of anabolic steroids (i.e. irritability, aggression)[31]. Mood disorders are not prevalent among **young** males with low doses of chronic abuse (less than 300mg/week) [46]. The dose of testosterone proposed here (intermittent administration of 100mg/week) is well below this dose and **an order of magnitude below** the doses where mood disorders are more likely to present (i.e. more than 1000mg per week) [46,47]. While the effects of testosterone replacement on mood changes during bed rest are yet unknown, no additional behavioral or psychological issues are anticipated to those normally encountered during long term bed rest [32]. If anything, we expect that testosterone treatment will result in improved mood and reduced fatigue.	Subjects will be monitored weekly for changes in hormone concentrations and presentation of symptoms. Subjects will be checked for this periodically during the study by means of a blood test called a PSA (Prostate-Specific Antigen) level.
View	MRI: The risks associated with the MRI procedure are minor as it involves a static magnetic field and weak radio waves. These have not been known to cause health damage. This procedure, however, may cause episodes of claustrophobia as the subject will be required to enter an open ended well lit cylinder. The other risk associated with MRI's involves metals in and on the body. To lessen this risk subjects will be required to remove all metals including jewelry from their body. The noise generated by the MRI scanner can cause temporary hearing loss. Subjects will be provided with ear plugs. MRI scans will be performed by qualified MRI technicians in the UTMB Division of Radiology.	Subjects will remove all metallic objects before entering the MR environment or MR system room including hearing aids, beeper, cell phone, keys, eyeglasses, hair pins, barrettes, jewelry (including body piercing jewelry), watch, safety pins, paperclips, money clip, credit cards, bank cards, magnetic strip cards, coins, pens, pocket knife, nail clipper, steel-toed boots/shoes, and tools. Loose metallic objects are especially prohibited in the MR system room and MR environment. Subjects with metals within their body will be assessed by a study physician before they are allowed to proceed with the MRI procedure. Safety may be assessed using the following source:

		www.mrisafety.com.<br><br>Subject will be provided with earplugs to reduce noise and still allow communication during the MRI scan.
View	Anemia: Repeated blood draws or excessive sampling volume increases the risk of anemia.	We will draw <400 mL of blood during the entire protocol.
View	Deep Venous Thrombosis (DVT): While there have been no reported DVT events associated with previous bed rest studies, there is a small risk of a DVT as a result of reduced physical activity or bed rest.	Long term immobility and dehydration increases the risk of Deep Vein Thrombosis (DVT).<br>While there have been no reported DVT events associated with previous bed rest studies, there is a small risk of a DVT as a result of reduced physical activity or bed rest.<br>Subjects may move legs in supine position and are encouraged to maintain adequate hydration. Risk will be reduced in subjects randomized into one of the exercise groups.
View	Phlebotomy: The risks of phlebotomy include vaso-vagal reaction, pain and infection if aseptic procedure is not used.	The risks of phlebotomy include vaso-vagal reaction, pain and infection if aseptic procedure is not followed.<br><br>Aseptic procedures will be followed.
View	Exercise Testing: Risks may include muscle tightness, soreness, fatigue, inability to perform physical activity, and rarely a muscle strain or tear. In older individuals, there is a small risk of myocardial infarction during maximal exercise tests. This is generally assumed to be approximately 1 in 10,000.	Exercise will be supervised by trained personnel to prevent injury as per iRATS long duration bed rest protocol.
View	Muscle Biopsies: Minor discomfort or pain, muscle soreness, bruising following muscle biopsy, minor bleeding during muscle biopsy, Infection, numb or tingling sensation lasting a period of days or months.<br><br>Drs. Sheffield-Moore, Paddon-Jones, Dillon, and Durham have together performed more than 2000 muscle biopsies with no serious side effects or complications, and have been certified by UTMB to perform such procedures. The vastus lateralis is regarded as a safe choice for biopsy sampling as there are no large vessels or major nerves in this region. The most common incidents associated with this procedure are bruising or black-and-blue marks in 1.4% of subjects, bleeding from the biopsy site in 0.2% of subjects, and pain lasting more than 3 days in 0.3% of subjects [48]. We will anesthetize the skin and subcutaneous tissue and make a 6-7 mm incision. A Trochar biopsy needle (Bergström) will be advanced through the fascia of the muscle with the cutting window closed. Then, with suction applied, the cutting cylinder will be opened and closed several times to collect tissue.	To minimize the risk of infection and bruising, an antibiotic ointment and pressure dressing are applied. Following the muscle biopsy, the skin will be closed with either sutures or liquid bonding agent and dressed with a compression bandage. Pain during tissue sampling will be minimized or eliminated with local anesthesia, while muscle soreness for the following 24-48 hours is easily controlled with over-the-counter strength ibuprofen or acetaminophen, and cold packs. The risk of infection is minimized by the use of aseptic technique and the application of antibiotic ointment. If infection is diagnosed, cultures will be taken and appropriate antibiotic therapy administered until resolution.
View		

iDXA: The risk involves low levels of radiation exposure (less than 0.3 mrem/whole body scan and less than 2.3 mrem including regional scans including AP Spine, Femurs, Calcaneus and Forearm for a person of average body size).	Subjects will be made aware of their exposure and risks prior to the scans and will have the choice to withdraw.

2.0 * **Has hazard analysis been completed?** ◉ **Yes** ○ No

3.0 **If completed please attach hazard analysis:**

Description	ID	Date Modified
Hazard Analysis	ID00000024	2/28/11

ID: Pro0028

View: 5.7 Potential Benefits and Alternatives

5.7 - Potential Benefits and Alternatives

1.0 * **Are there potential direct benefits to study subjects?**

No, there is no direct benefit to subjects in this study.

If YES, please describe any potential for direct benefits to participants in this study

2.0 **Describe any potential benefits to Society or Space Flight:**

Results from this study will further elucidate the role of testosterone in the maintenance of skeletal muscle and bone during long term bed rest as a model for space flight.

It is hypothesized that maintaining appropriate balance between hormonal status, nutritional status, and physical activity during spaceflight is of critical importance in preventing musculoskeletal losses. It is our contention that the restoration of physiologic levels of testosterone is essential to prevent and/or restore spaceflight- and microgravity-induced losses in the musculoskeletal system. Current evidence suggests that the combination of testosterone and exercise will optimize the effectiveness of the existing exercise and nutritional countermeasures.

3.0 * **Are there any alternatives to study participation available to prospective subjects?**

Choose not to participate

4.0 **If other, please describe alternatives (research or non-research) that are available to subjects if they choose not to participate in all aspects of this study:**

ID: Pro0028

View: 6.0 Data Privacy & Confidentiality

6.0 - Data Privacy & Confidentiality

Privacy concerns people, whereas confidentiality concerns data. Specifically, confidentiality refers to the researcher's plan to handle, manage and disseminate the participant's identifiable private information. Privacy refers to a person's wish to control the access of others to themselves.

Use the following statement:

Privacy/Confidentiality (for Research Protocol)

Subject privacy and data confidentiality will be maintained in accordance with 1) NASA Policy Directive (NPD) 7100.8, "Protection of Human Research Subjects"; 2) NASA Procedural Requirements (NPR) 7100.1, "Protection of Human Research Subjects"; and 3) to the extent allowed by Federal law.

1.0 * **Does this study fall under a Data and Safety Monitoring Plan (DSMP)?** Definition (●) **Yes** ( ) No

2.0 * **How will the data for this study be collected and recorded?**

All data collected will be de-identified and will include variables such as age, ethnicity, anthropometric measurements, and biological measurements (excluding genetic testing), MRI/iDXA scans, and strength/fatigue measures. Data from radiologic scans will be de-identified by UTMB information services (IS) personnel within the ITS-CRC before being released to the PI and CO-I's. Data will be stored electronically on access restricted servers or as hard-copies in locked cabinets within locked rooms at UTMB. Files will be password protected before sharing with non-UTMB bed rest.

3.0 * **Select Data Recording Identifiers used on this study:**

Name
Data are kept in locked file cabinet
Data are kept in locked office or suite
Data are stored on a secure network

4.0 **If Other is selected, please specify:**

5.0 **Please describe the encryption method employed to protect the data.**

Sensitive Digital Data, as defined by UTS 165, includes social security numbers, Protected Health Information (PHI), Sensitive Research Data, digital Data associated with an individual and/or digital Data protected by law. Sensitive digital Data must be secured and protected while at rest (electronic storage on a hard drive, digital or optical media), mobile (laptop, PDA or flash drive) and in transit (via email or the Internet). The data shall be encrypted with at least 128-bit encryption. Current standards are: Transport Layer Security/Secure Socket Layer=128 bit, Data at Rest=256 AES References: http://www.utmb.edu/infosec/Policies/UTMB-ISOPublication100-1A.pdf
http://www.utmb.edu/InfoSec/Policies/PS/PS129_Encryption.pdf

6.0 **Please describe the authentication methods use to ensure the security of the database.**

UTMB login credentials from authorized users

7.0 * **Who, other than the specified study team, will have access to the study records or data? Specify their name, role, and affiliation.**

By signing the subject consent form, subjects agree that data collected as part of the Flight Analogs

Project may be shared, using only the number identifier, among appropriate investigators and collaborators of this study and their experiment personnel. All investigators, collaborators and experiment personnel in direct contact with research subjects will have completed the required Collaborative Institutional Training Initiative (CITI) online training.

The Life Sciences Data Archive (LSDA) will resume responsibility for archival of data according to a Data Submission Agreement to be developed with representatives of LSDA.

8.0 * **How will the investigator maintain privacy in the research setting(s)?**

All study data will be collected by the research team, reviewed by the PI, and stored in secure, locked files and/or databases in order to protect it from inadvertent loss or improper access. Data and other records will be identified by coded number only to maintain subject confidentiality. All the information obtained in connection with these studies will remain confidential as far as possible within state and federal law. Information gained from this study that can be linked to the subject's identity will not be released to anyone other than the investigators, the subject and the subject's physician. The results of these studies will be published in scientific journals without identifying the subjects by name.

9.0 * **What are the consequences to participants of a loss of privacy (eg., risks to reputation, insurability, other social risks)?**

Sense of insecurity due to public exposure, embarrassment, percieved loss of control of person, loss of trust by the subject toward the research team.

10.0 * **If coded or identified data will be released, specify the persons/agencies to whom the information will be released. Please also indicate the provisions that will be taken to assure that the transmission of the data will maintain confidentiality:**

All study data will be collected by the research team, reviewed by the PI, and stored in secure, locked files and/or databases in order to protect it from inadvertent loss or improper access. Data and other records will be identified by coded number only to maintain subject confidentiality. All the information obtained in connection with these studies will remain confidential as far as possible within state and federal law. Information gained from this study that can be linked to the subject's identity will not be released to anyone other than the investigators, the subject and the subject's physician. The results of these studies will be published in scientific journals without identifying the subjects by name.

11.0 * **When the study is completed and the data is submitted and accepted by NASA, please indicate your plans for the destruction of the local dataset.**

Hardcopies of any data to be destroyed will be shredded. Electronic files containing study data will deleted from the UTMB network.

12.0 * **Is this study collecting health information?** (•) **Yes** ( ) No

Describe any additional steps taken to assure that identities of subjects and any of their health information which is protected under the law is kept confidential. If photography, video or audio recordings will be made as part of the study, disposition of these recordings should be addressed here and in the consent form.

Subject privacy and data confidentiality will be maintained in accordance with 1) NASA Policy Directive (NPD) 7100.8, “Protection of Human Research Subjects”; 2) NASA Procedural Requirements (NPR) 7100.1, “Protection of Human Research Subjects”; and 3) to the extent allowed by Federal law.

ID: Pro0028

View: 6.2 Non-Astronaut data Collection

6.2 - Non-Astronaut Data Collection

1.0 * **Please select all data types collected during this study:** (Check all that apply)

Data Type
Age
Gender
Race

2.0 **If other please specify:**

Weight, Height, History & Physical, DEXA, MRI, Tissues (blood, muscle, urine, saliva).

ID: Pro0028

View: 6.3 Data Safety Monitoring Plan

6.3 - Data Safety Monitoring Plan

1.0 * **Check the one box below that most accurately reflects the plan for data and safety monitoring for this study.**

	Name
○	The study will be monitored only by the study investigators and/or sponsor.
◉	The study will be monitored by at least one individual who is not associated with the study, but not by a formally constituted Data and Safety Monitoring Board (DSMB).
○	A formally constituted Data and Safety Monitoring Board (DSMB) will monitor the study.

2.0 * **Describe the clinical criteria for withdrawing an individual subject from the study due to safety or toxicity concerns:**

According to FAP procedures and exclusion criteria.

3.0 * **Summarize any pre-specified criteria for stopping or changing the study protocol due to safety concerns.**

The subject will be withdrawn from the study when: -Exceeding FAP and exclusion criteria. - Exceeding normal ranges in clinical parameters or as advised by the safety monitor.

4.0 * **Are there any plans to perform an interim efficacy analysis:**

○ Yes ◉ **No**

5.0 **If you answered Yes, please describe the plans to conduct an interim analysis.**

N.A.

ID: Pro0028 View: 7.0 Consent Forms & Process of Consent

7.0 - Consent Forms & Process of Consent

1.0 Will this study include non-English speaking participants? ○ Yes ● **No**

2.0 We've prepared several different types of consent form templates. Please follow the instructions below to complete the process.

Instructions:
2.1) Download the applicable consent form/subject information handout template to your machine and modify this where applicable.

- Engineering Consent and Lay Summary Template
- Multinational Space Station Human Research Informed Consent
- Research Consent and Lay Summary Template

* **2.2) Upload consent forms/subject information handouts, assent forms here:**

Name	Modified	Version
NASA Consent Form - Urban | History	1/16/2011 11:17 AM	0.01
Subject Information Handout - Layman Summary Testosterone Urban | History	6/2/2011 1:44 PM	0.02
Video and Photo Consent Form | History	5/8/2011 2:55 AM	0.01

3.0 Describe how, when, and where the consent process will be initiated:

Standard FAP procedures will be used.

4.0 Who will obtain informed consent from subjects for this research?

Standard FAP procedures will be used. The complement PI and Co-I's will obtain consent.

8.0 References

1.0 References:

1. Leonard JI, Leach CS, Rambaut PC. Quantitation of tissue loss during prolonged space flight. *Am J Clin Nutr.* Nov 1983;38(5):667-679.

2. Berry CA. The medical legacy of Gemini. *Life Sci Space Res.* 1968;6:1-19.

3. LeBlanc AD, Spector ER, Evans HJ, Sibonga JD. Skeletal responses to space flight and the bed rest analog: a review. *J Musculoskelet Neuronal Interact.* Jan-Mar 2007;7(1):33-47.

4. Smith SM, Wastney ME, Morukov BV, et al. Calcium metabolism before, during, and after a 3-mo spaceflight: kinetic and biochemical changes. *Am J Physiol.* Jul 1999;277(1 Pt 2):R1-10.

5. Smith SM, Wastney ME, O'Brien KO, et al. Bone markers, calcium metabolism, and calcium kinetics during extended-duration space flight on the mir space station. *J Bone Miner Res.* Feb 2005;20(2):208-218.

6. Smith SM. Red blood cell and iron metabolism during space flight. *Nutrition.* Oct 2002;18 (10):864-866.

7. Alfrey CP, Udden MM, Leach-Huntoon C, Driscoll T, Pickett MH. Control of red blood cell mass in spaceflight. *J Appl Physiol.* Jul 1996;81(1):98-104.

8. Talbot JM, Fisher KD. Influence of space flight on red blood cells. *Fed Proc.* Aug 1986;45 (9):2285-2290.

9. Taylor WE, Bhasin S, Lalani R, Datta A, Gonzalez-Cadavid NF. Alteration of gene expression profiles in skeletal muscle of rats exposed to microgravity during a spaceflight. *J Gravit Physiol.* Dec 2002;9(2):61-70.

10. Strollo F, Boitani C, Basciani S, et al. The pituitary-testicular axis in microgravity: analogies with the aging male syndrome. *J Endocrinol Invest.* 2005;28(11 Suppl Proceedings):78-83.

11. Wade CE, Stanford KI, Stein TP, Greenleaf JE. Intensive exercise training suppresses testosterone during bed rest. *J Appl Physiol.* Jul 2005;99(1):59-63.

12. Simental JA, Sar M, Lane MV, French FS, Wilson EM. Transcriptional activation and nuclear targeting signals of the human androgen receptor. *J Biol Chem.* Jan 5 1991;266(1):510-518.

13. Gentile MA, Nantermet PV, Vogel RL, et al. Androgen mediated improvement of body composition and muscle function involves a novel early transcriptional program including IGF-1, MGF and induction of beta-catenin. *J Mol Endocrinol.* Sep 24 2009.

14. Sinha-Hikim I, Roth SM, Lee MI, Bhasin S. Testosterone-induced muscle hypertrophy is associated with an increase in satellite cell number in healthy, young men. *Am J Physiol Endocrinol Metab.* Jul 2003;285(1):E197-205.

15. Singh R, Bhasin S, Braga M, et al. Regulation of myogenic differentiation by androgens: cross talk between androgen receptor/ beta-catenin and follistatin/transforming growth factor-beta signaling pathways. *Endocrinology.* Mar 2009;150(3):1259-1268.

16. Khosla S, Atkinson EJ, Dunstan CR, O'Fallon WM. Effect of estrogen versus testosterone on circulating osteoprotegerin and other cytokine levels in normal elderly men. *J Clin Endocrinol Metab.* Apr 2002;87(4):1550-1554.

17. Malkin CJ, Pugh PJ, Jones RD, Kapoor D, Channer KS, Jones TH. The effect of testosterone replacement on endogenous inflammatory cytokines and lipid profiles in hypogonadal men. *J Clin Endocrinol Metab.* Jul 2004;89(7):3313-3318.

18. Yesilova Z, Ozata M, Kocar IH, et al. The effects of gonadotropin treatment on the immunological features of male patients with idiopathic hypogonadotropic hypogonadism. *J Clin Endocrinol Metab.* Jan 2000;85(1):66-70.

19. Lieberman SA, Oberoi AL, Gilkison CR, Masel BE, Urban RJ. Prevalence of neuroendocrine dysfunction in patients recovering from traumatic brain injury. *J Clin Endocrinol Metab.* Jun 2001;86 (6):2752-2756.

20. Strollo F, Riondino G, Harris B, et al. The effect of microgravity on testicular androgen secretion. *Aviat Space Environ Med.* Feb 1998;69(2):133-136.

21. Amann RP, Deaver DR, Zirkin BR, et al. Effects of microgravity or simulated launch on testicular function in rats. *J Appl Physiol.* Aug 1992;73(2 Suppl):174S-185S.

22. Popova IA, Afonin BV, Vetrova EG, Drozdova TE, Zagorskaia EA. [Homeostatic reactions of the blood of experimental rats after "Kosmos-1667" flight]. *Kosm Biol Aviakosm Med.* Sep-Oct 1988;22(5):39-42.

23. Wimalawansa SM, Wimalawansa SJ. Simulated weightlessness-induced attenuation of testosterone production may be responsible for bone loss. *Endocrine.* Jun 1999;10(3):253-260.
24. Wimalawansa SM, Wimalawansa SJ. A novel pharmacological approach of musculoskeletal losses associated with simulated microgravity. *J Musculoskelet Neuronal Interact.* Sep 2000;1(1):35-41.
25. Zachwieja JJ, Smith SR, Lovejoy JC, Rood JC, Windhauser MM, Bray GA. Testosterone administration preserves protein balance but not muscle strength during 28 days of bed rest. *J Clin Endocrinol Metab.* Jan 1999;84(1):207-212.
26. Ferrando AA, Sheffield-Moore M, Yeckel CW, et al. Testosterone administration to older men improves muscle function: molecular and physiological mechanisms. *Am J Physiol Endocrinol Metab.* Mar 2002;282(3):E601-607.
27. Ferrando AA, Sheffield-Moore M, Wolf SE, Herndon DN, Wolfe RR. Testosterone administration in severe burns ameliorates muscle catabolism. *Crit Care Med.* Oct 2001;29(10):1936-1942.
28. Sheffield-Moore M, Paddon-Jones D, Casperson SL, et al. Androgen therapy induces muscle protein anabolism in older women. *J Clin Endocrinol Metab.* Oct 2006;91(10):3844-3849.
29. Jockenhovel F, Minnemann T, Schubert M, et al. Timetable of effects of testosterone administration to hypogonadal men on variables of sex and mood. *Aging Male.* Dec 2009;12(4):113-118.
30. Bain J. Testosterone and the aging male: to treat or not to treat? *Maturitas.* May 2010;66 (1):16-22.
31. Daly RC, Su TP, Schmidt PJ, Pagliaro M, Pickar D, Rubinow DR. Neuroendocrine and behavioral effects of high-dose anabolic steroid administration in male normal volunteers. *Psychoneuroendocrinology.* Apr 2003;28(3):317-331.
32. Seaton KA, Bowie KE, Sipes WA. Behavioral and psychological issues in long-duration head-down bed rest. *Aviat Space Environ Med.* May 2009;80(5 Suppl):A55-61.
33. Stein KD, Martin SC, Hann DM, Jacobsen PB. A multidimensional measure of fatigue for use with cancer patients. *Cancer Pract.* May-Jun 1998;6(3):143-152.
34. Mendoza TR, Wang XS, Cleeland CS, et al. The rapid assessment of fatigue severity in cancer patients: use of the Brief Fatigue Inventory. *Cancer.* Mar 1 1999;85(5):1186-1196.
35. Bergstrom J, Furst P, Nore LO, Vinnars E. Intracellular free amino acid concentration in human muscle tissue. *J App Physiol.* 1974;36:693-697.
36. Sheffield-Moore M, Urban RJ, Wolf SE, et al. Short-term oxandrolone administration stimulates net muscle protein synthesis in young men. *J Clin Endocrinol Metab.* Aug 1999;84 (8):2705-2711.
37. Paddon-Jones D, Sheffield-Moore M, Urban RJ, et al. Essential amino acid and carbohydrate supplementation ameliorates muscle protein loss in humans during 28 days bedrest. *J Clin Endocrinol Metab.* Sep 2004;89(9):4351-4358.
38. Dillon EL, Sheffield-Moore M, Paddon-Jones D, et al. Amino acid supplementation increases lean body mass, basal muscle protein synthesis, and insulin-like growth factor-I expression in older women. *J Clin Endocrinol Metab.* May 2009;94(5):1630-1637.
39. Dreyer HC, Glynn EL, Lujan HL, Fry CS, Dicarlo SE, Rasmussen BB. Chronic paraplegia-induced muscle atrophy downregulates the mTOR/S6K1 signaling pathway. *J Appl Physiol.* Sep 20 2007.
40. Dreyer HC, Fujita S, Cadenas JG, Chinkes DL, Volpi E, Rasmussen BB. Resistance exercise increases AMPK activity and reduces 4E-BP1 phosphorylation and protein synthesis in human skeletal muscle. *J Physiol.* Oct 15 2006;576(Pt 2):613-624.
41. Fujita S, Dreyer HC, Drummond MJ, et al. Nutrient Signalling in the Regulation of Human Muscle Protein Synthesis. *J Physiol.* May 3 2007.
42. Kawada T, Muto K, Kanai T, Kuratomi Y. Prostate-specific antigen screening of workers under the age of 40 in Japan. *Cancer Epidemiol.* Oct 2009;33(3-4):309-310.
43. Lim LS, Sherin K. Screening for prostate cancer in U.S. men ACPM position statement on preventive practice. *Am J Prev Med.* Feb 2008;34(2):164-170.
44. Farley SJ. Prostate cancer: Risk stratification of PSA-based screening. *Nat Rev Urol.* Dec 2010;7(12):643.
45. Svetec DA, Canby ED, Thompson IM, Sabanegh ES, Jr. The effect of parenteral testosterone replacement on prostate specific antigen in hypogonadal men with erectile dysfunction. *J Urol.* Nov 1997;158(5):1775-1777.
46. Pope HG, Jr., Katz DL. Psychiatric and medical effects of anabolic-androgenic steroid use. A

controlled study of 160 athletes. *Arch Gen Psychiatry.* May 1994;51(5):375-382.
47. van Amsterdam J, Opperhuizen A, Hartgens F. Adverse health effects of anabolic-androgenic steroids. *Regul Toxicol Pharmacol.* Jun 2010;57(1):117-123.
48. Highstead RG, Tipton KD, Creson DL, Wolfe RR & Ferrando AA. Incidence of associated events during the performance of invasive procedures in healthy human volunteers. J Appl Physiol 2005;98(4):1202-1206.
49. Wang Y, DeLuca HF. Is the vitamin d receptor found in muscle? Endocrinology 2010;152:354-63.
50. Wehr E, Pilz S, Boehm BO, Marz W, Obermayer-Pietsch B. Association of vitamin D status with serum androgen levels in men. Clin Endocrinol (Oxf) 2010;73:243-8.
51. Ramlau-Hansen CH, Moeller UK, Bonde JP, Olsen J, Thulstrup AM. Are serum levels of vitamin D associated with semen quality? Results from a cross-sectional study in young healthy men. Fertil Steril 2010;95:1000-4.
52. Pilz S, Frisch S, Koertke H, et al. Effect of vitamin D supplementation on testosterone levels in men. Horm Metab Res 2010;43:223-5.

ID: Pro0028

View: 8.1 - Miscellaneous Documents

8.1 Miscellaneous Documents

1.0 Please upload any miscellaneous documents you feel might assist the board in their deliberations.

	Description
View	DSMP

ID: Pro0028

View: SF - Final Page

Final Page

You have completed your application!

Please check hide/show errors prior to selecting "Finish" and correct any listed errors you will not be able to submit a study for review with out resolving all errors.

Please hit "Finish" to finalize and exit the application. Doing so will NOT submit the application for review.

Please note that a submission may only be forwarded to the IRB by the Principal Investigator. To do this, the Principal Investigator must press the "SUBMIT STUDY" button in My Activities for this Study ID:Pro0028.

You can track the ongoing status of your submission by logging into the study workspace.

Please feel free to contact the IRB with any questions or concerns.

ID: Pro0028 View: Print View

*** Institution Name:**
The University of Texas Medical Branch

*** Country:**
USA

State:
TX

*** City:**
Galveston

*** IRB Approval Status:**
Approved

Description:

Approval letter:
UTMB IRB Approval Letter(0.01)
